# Rationally Designed Phenyl- and Phenanthryl-Based Imidazole Hybrids for the Treatment of Breast Cancer—A Structure–Activity Relationship Study

**DOI:** 10.3390/molecules31203722

**Published:** 2026-10-16

**Authors:** Bharathi Hassan Ganesh, Preethi G. Anantharaju, Amritha Sukumaran, Carmelo Drago, Sarthak Mohanty, Himabindu Padinjarathil, SubbaRao V. Madhunapantula, Prasanna Ramani

**Affiliations:** 1 Dhanvanthri Laboratory, Department of Chemistry, Amrita School of Physical Sciences, Amrita Vishwa Vidyapeetham, Coimbatore 641112, India; hg_bharathi@cb.students.amrita.edu (B.H.G.); s_amritha@cb.students.amrita.edu (A.S.); sarthakmohanty997@gmail.com (S.M.); p_himabindu@cb.students.amrita.edu (H.P.); 2 Center of Excellence in Advanced Materials and Green Technologies (CoE–AMGT), Amrita School of Engineering, Amrita Vishwa Vidyapeetham, Coimbatore 641112, India; 3 Center of Excellence in Molecular Biology and Regenerative Medicine (CEMR), Department of Biochemistry, JSS Medical College, JSS Academy of Higher Education & Research, Mysore 570015, India; preethijinesh@gmail.com; 4 Center of Excellence in Cell Biology & Proteomics, College of Pharmaceutical Sciences, School of Health Science, Dayananda Sagar University, Bangalore 562112, India; 5 Institute of Biomolecular Chemistry, CNR, Via Paolo Gaifami 18, 95126 Catania, Italy; carmelo.drago@cnr.it

**Keywords:** imidazole, one-pot synthesis, *N*-alkylation reaction, in vitro antiproliferation, cell cycle arrest, reactive oxygen species, in silico studies

## Abstract

A series of 11 compounds of phenyl–imidazole–pyrrole and 9 compounds of phenanthryl–imidazole–pyrrole were synthesized and evaluated against breast cancer cell lines differing in the expression of estrogen receptor (ER), progesterone receptor (PR) and human epidermal growth factor receptor (HER)-2, viz., BT-474 (ER+, PR+, and HER2+), T-47D and MCF-7 (ER+, PR+, and HER2−), and MDA-MB-468 and MDA-MB-231 (ER−, PR−, and HER2−). The analysis of the results indicated that the most active compounds were **5d** and **5k** from the phenyl–imidazole–pyrrole series and **6b** and **6i** from the phenanthryl–imidazole–pyrrole series (IC_50_ < 50 µM). Molecular docking (MD) and molecular dynamics simulation (MDS) studies demonstrated that the active compounds exhibited high binding affinity to the 7NH5 (a co-crystal structure of Akt1 and its specific inhibitor) protein, which is overexpressed in many breast cancer types. The active compounds **6b** and **6i** reduced the viability of MDA-MB-231 and MCF-7 cells by inducing cell death mechanisms. Both compounds halted the proliferation by arresting cells in the G0/G1 phase of the cell cycle. The results are similar to those of traditional Cisplatin. Intraperitoneal administration of **6i** retarded the development of EAC tumors and increased the survival of mice compared with tumor-bearing control and 5-fluorouracil (5FU)-treated positive control mice.

## 1. Introduction

Imidazole is a validated pharmacophore for targeting the PI3K/AKT/mTOR axis in breast cancer, as tri-substituted imidazoles have been shown to induce apoptosis in breast cancer cells by downregulating Akt/PDK/mTOR phosphorylation. Docking studies have shown the strong binding of tri-substituted imidazoles to Akt2. Since Akt drives the proliferation of cancer cells via the activation of cyclin D1 while suppressing the expression of p21/p27, and p53, rationally targeting this survival kinase with these molecules is a viable strategy, and the tri-substituted imidazole scaffold is a preferred choice for drug design and development [1,2]. Hence, in recent years, tri- and tetra-substituted imidazole derivatives have emerged as highly versatile scaffolds in medicinal chemistry. Their structural diversity, often achieved through efficient multicomponent reactions such as the Radziszewski synthesis, allows for the fine-tuning of physicochemical and pharmacological properties [3,4,5,6]. Docking and pharmacokinetic studies further confirmed their favorable target interactions. Both tri- and tetra-substituted analogs have also demonstrated promising anticancer properties due to their ability to inhibit proliferation of various cell lines, such as MDA-MB-231 (breast) [7], MIA-PaCa-2 (pancreatic) and HeLa (cervical). Additionally, selected derivatives have exhibited ancillary antibacterial and antifungal activities [8], highlighting their pleiotropic potential to retard the growth of diverse organisms. Collectively, the broad pharmacological profile of these compounds, which extends to anti-inflammatory [9], anticancer [10,11], antimicrobial [12], enzyme inhibitory [13], and antiviral actions, underscores their value as lead structures in drug discovery. Their ease of synthesis, compatibility with diverse substitution patterns, and strong structure–activity relationships (SARs) make tri- and tetra-substituted imidazoles the compounds of choice for the development of next-generation therapeutics.

Based on updated projections from the International Agency for Research on Cancer (IARC), the most common types of cancer are breast, lung, colorectal, prostate, hematological malignancies and ovarian cancer [14]. Breast cancer (BC) is a major global health issue, with the World Health Organization (WHO) reporting 7.8 million women being diagnosed in the past three years, making it the most prevalent cancer worldwide [15]. BC accounts for 4–5.5% of all new cancer cases annually, significantly contributing to morbidity rates [16]. Women are more susceptible to BC due to non-modifiable risk variables such as age, genetic mutations, heredity, early menstruation and late onset of menopause [17]. Chemotherapy is still the cornerstone of cancer treatment, and research efforts continue to increase the effectiveness while minimizing the side effects [18]. Estrogen receptor (ER), progesterone receptor (PR) and human epidermal growth factor receptor 2 (HER2) are critical molecular markers in breast cancer, playing significant roles in prognosis and treatment decisions. ER and PR are hormone receptors that when positive, indicate a better prognosis and responsiveness to hormone therapies, while HER2 positivity is associated with more aggressive disease but can be targeted with specific therapies [19]. The expression of these markers varies between primary tumors and metastatic sites, which can impact treatment strategies. HER2-positive breast cancers, which account for 15–30% of cases, often have a worse prognosis but benefit from HER2-targeted treatments [20]. The assessment of these markers is essential to personalized treatment plans, and their expression can be influenced by various factors, including age, menopausal status, and tumor characteristics [18]. Understanding the interplay among ER, PR, and HER2 is crucial to optimizing breast cancer management and improving patient outcomes. In the absence of ER, PR and HER2 expression, triple-negative breast cancer (TNBC) cells are primarily targeted using compounds that elevate intracellular ROS levels, leading to mitochondrial dysfunction and apoptotic cell death [21]. Common chemotherapy drugs based on the imidazole scaffold are Dacarbazine, Zoledronic acid, Nilotinib and Asciminib. Although imidazole scaffolds have demonstrated significant anticancer potential, particularly in tri- and tetra-substituted derivatives, none of these has received FDA approval for breast cancer therapy to date.

In order to modify lipophilicity and membrane permeability for optimal cellular uptake and to investigate accessory hydrophobic contacts close to the kinase’s solvent-exposed region, the N1-position was varied with lipophilic alkyl chains and aryl groups (tolyl, *m*-phenol, *p*-fluorophenyl, etc.). This approach is consistent with reports that N1-substituted imidazole hybrids exhibit strong antiproliferative activity against MCF-7 cells with substituent-dependent potency, supporting *N*1-decoration as a tunable handle for activity optimization. The second position of imidazole was decorated with an *N*-methylpyrrole unit since pyrrole derivatives have been docked against AKT among key breast cancer targets, and AKT1 has been co-crystallized with a pyrrolopyrimidine inhibitor, confirming that pyrrole-fused heterocycles are well accommodated in its ATP-binding pocket; *N*-methylation removes the pyrrole N–H to avoid a labile, tautomerizable site while retaining planarity for kinase engagement. Phenyl and phenanthryl groups were placed at the fourth and fifth positions as a matched pair to probe how aromatic surface area affects AKT1 binding, since extended polyaromatic AKT1 inhibitor candidates have shown markedly stronger binding affinity than the clinical inhibitor ipatasertib by occupying allosteric subpockets more fully than smaller aromatics, with phenyl serving as the compact reference and phenanthryl testing whether greater π-surface improves potency against breast cancer cell lines [22,23].

The objective of this study is to synthesize a series of tri- and tetra-substituted imidazole derivatives as effective inhibitors of breast cancer cell proliferation. The electronic properties of the imidazole scaffold were modulated through variation in the R substituent at position 1, introduced either from selected primary amines or via alkyl halides. Position 2 of the imidazole ring was functionalized with a pyrrole unit, given the extensive investigation of pyrrole derivatives for their anti-breast cancer potential, particularly in MCF-7 cell models. Positions 4 and 5 were initially substituted with phenyl (Ph) groups. To further explore how molecular planarity influences biological activity, the phenyl rings were replaced with a phenanthryl moiety in the subsequent series of compounds. Despite the fact that the 9,10-phenanthrenequinone (PQ) compound is known to form complexes with transition elements, these compounds still remain minimally investigated. These compounds have been shown to exhibit mutagenesis by acting as a substrate for the P-450 reductase enzyme and eventually leading to the production of ROS. Therefore, in this research study, new phenyl–imidazole–pyrrole and phenanthryl–imidazole–pyrrole compounds were synthesized, and their antiproliferative activity and impact on cell cycle analysis were evaluated in vitro in various breast cancer cell lines. Tumor reduction potential was evaluated using the Ehrlich Ascites Carcinoma (EAC) solid tumor model in mice. In addition, computational analyses that include molecular docking, energy calculation and molecular dynamics simulation of the synthesized compounds were executed.

## 2. Results

### 2.1. Synthesis

To conduct a comparative analysis with different substitutions on the imidazole nitrogen, a series of 2,4,5-tri-substituted imidazoles and 1,2,4,5-tetra-substituted imidazoles were synthesized. These substitutions included various aromatic groups as well as aliphatic chains with an increase in the number of carbon atoms. The functionalities found in the imidazole’s fourth and fifth positions influenced the electronic effects on imidazole scaffolds. The phenyl and phenanthryl units occupied positions four and five of the imidazole scaffold respectively, while the *N*-methyl pyrrole unit was positioned at the second position. To achieve this goal, the synthesis of an imidazole–pyrrole series of compounds using a one-pot reaction and a *N*-alkylation reaction was performed, and the synthesized compounds were purified with silica gel column chromatography and characterized using various spectroscopic techniques (^1^H- NMR, ^13^C-NMR, IR, and mass analysis). In total, fourteen novel imidazole–pyrrole compounds were synthesized and characterized (Supporting Information).

#### 2.1.1. Synthesis of 2,4,5-Tri-Substituted Imidazole Hybrid Compounds

**Scheme 1:**
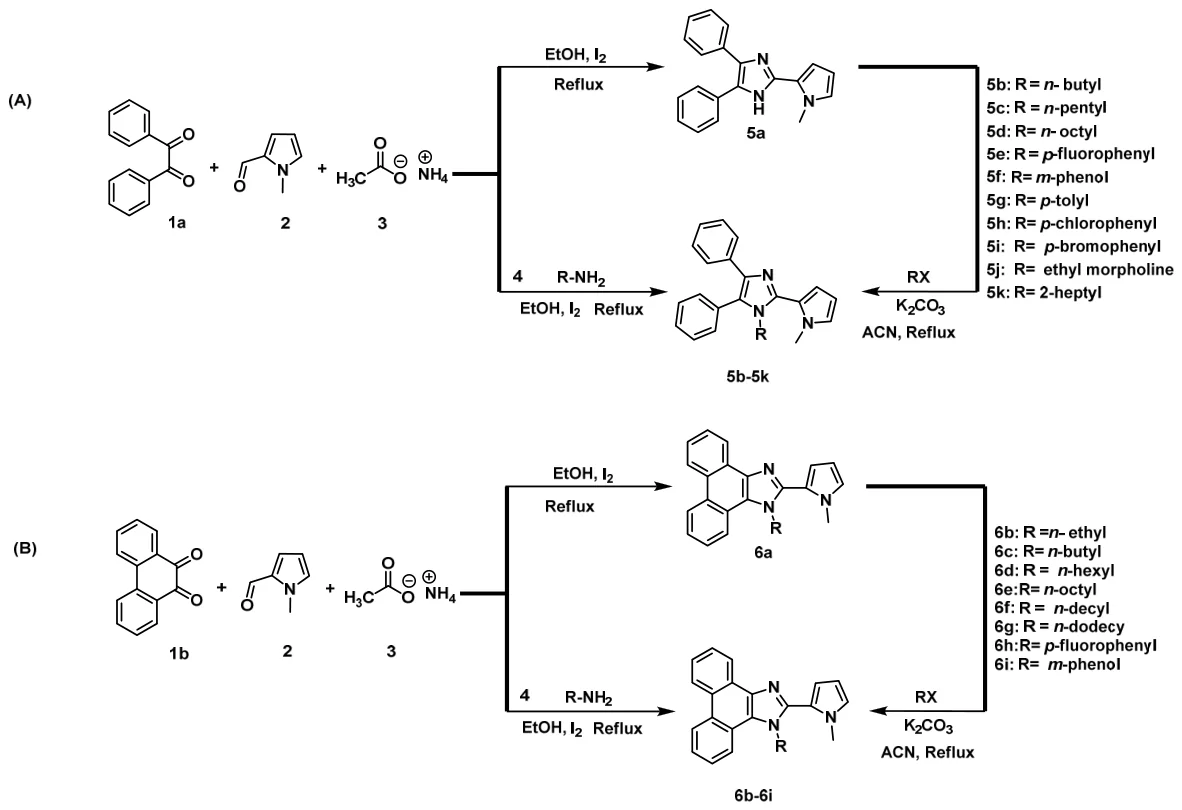
Synthesis of (**A**) phenyl imidazole- *N*-methyl pyrrole compounds and (**B**) phenanthryl–imidazole-*N*-methyl pyrrole compounds.

The synthesis of 2,4,5-tri-substituted imidazole derivatives was efficiently accomplished through a one-pot, multicomponent reaction. Benzil (1 eq) (1a), *N*-methyl pyrrole-2-carboxaldehyde (1 eq) (2), ammonium acetate (3 eq) (3), and a catalytic amount of iodine were dissolved in ethanol, and the resulting reaction mixture was stirred at 75–80 °C for 16 h. The same protocol was followed by using 9,10-phenanthraquinone (1b) instead of benzil (Scheme 1). The progress of the reactions was monitored through thin-layer chromatography (TLC) with visualization under UV light. The DNP staining reagent confirmed the completion of aldehyde. After complete consumption of the starting material, the mixture was cooled to room temperature, diluted with water (10 mL), and extracted with ethyl acetate (2 × 10 mL); the solvent was evaporated to obtain **5a** and **6a**. The crude products were purified using silica gel column chromatography (using ethyl acetate and petroleum ether (1:9)), which yielded pure products (62% and 60%, respectively). A deeper insight into the reaction mechanism revealed that strong Lewis acids create stable complexes with carbonyl oxygen, decreasing the activation energy for imine formation. FeCl_3_ and ZrOCl_2_ are strong Lewis acids, and despite being a weaker Lewis acid, iodine can also polarize the carbonyl group by transitory charge-transfer complexes or halogen bonding, particularly in mild or solvent-free environments [24,25]. Hence, the reaction was carried out with these catalysts in order to compare the rate and yield. All three catalysts accelerated the creation of C=N bonds via these activation routes (Table 1). Among all the catalysts, the usage of iodine yielded maximum product. Similar such observations were reported in the literature [26]. The iodine-catalyzed reaction mechanism is illustrated in Scheme S1 for both three-component and four-component reactions. The ^1^H NMR spectrum of compound **6a** represented all the desired signals. The –CH_3_ hydrogen attached to the pyrrole ring was observed as a singlet at 4.25 ppm. The pyrrole hydrogen peaks were observed at 6.50–6.26 ppm, 6.71 ppm, and 6.84 ppm, while the phenanthryl ring protons were observed at 7.60–7.64, 8.73 ppm and 9.76 ppm. Similarly, the results of ^13^C-NMR showed the required signals of the molecule. The FT-IR spectrum of **6a** represented most of the vibrational signals. The molecular weight of compound **6a** is 297.13, the observed mass is 298.33 [M+H]^+^, and LC purity was found to be 98%.

#### 2.1.2. Synthesis of 1,2,4,5-Tetra-Substituted Imidazole–Pyrrole Hybrid Compounds

Tetra-substituted imidazoles were synthesized by employing various primary amines (4), in addition to the process illustrated in Scheme 1. From primary amines 4 (1 eq), as another starting material, compounds **5b**, **5d**–**5k**, **6e**, **6h** and **6i** were synthesized by adopting similar processes as mentioned earlier. All the compounds were purified and structures confirmed by various spectroscopic techniques.

#### 2.1.3. *N*-Alkylation Reaction

A set of compounds were synthesized via the *N*-alkylation method as an alternative to using primary amines. Compounds **5c**, **6b**–**6d**, **6f**, and **6g** were synthesized by *N*-alkylating **5a** and **6a** (1 eq) with alkyl halides (1 eq) (4) as mentioned in Scheme 1. The reaction was performed using potassium carbonate (3 eq) as the base and acetonitrile as the solvent. The reaction mixture was stirred at 85–90 °C for 16 h. The reaction progress was monitored by using TLC, and upon the consumption of the starting material, the mixture was cooled to room temperature, diluted with water (10 mL), and extracted with ethyl acetate (2 × 10 mL). The combined organic layers were dried over anhydrous Na_2_SO_4_, filtered, and concentrated under reduced pressure to afford the crude product. Purification by silica gel column chromatography (60–120 mesh) using 0–5% ethyl acetate in petroleum ether as the eluent yielded the pure compound. The synthesis and characterization details of compound **5c** were reported in our previous work [11]. By using this scheme a total of five phenanthryl–imidazole–pyrrole compounds were synthesized. The physical parameters, including molecular weight, yield, and melting point, of all synthesized compounds are tabulated in Table S1.

### 2.2. In Vitro Studies

The cytotoxicity of each compound was examined against human breast cancer cell lines that varied in their ER, PR and HER2 expression. BT-474 (ER+, PR+ and HER2+), T-47D and MCF-7 (ER+, PR+ and HER2−), and MDA-MB-468 and MDA-MB-231 (ER−, PR− and HER2−) were used in this study to determine the effect of the synthesized compounds.

#### 2.2.1. Evaluation of the Antiproliferative Potential of Phenyl–Imidazole–Pyrrole (**5a**–**5k**) in Breast Cancer Cell Lines

**Figure 1:**
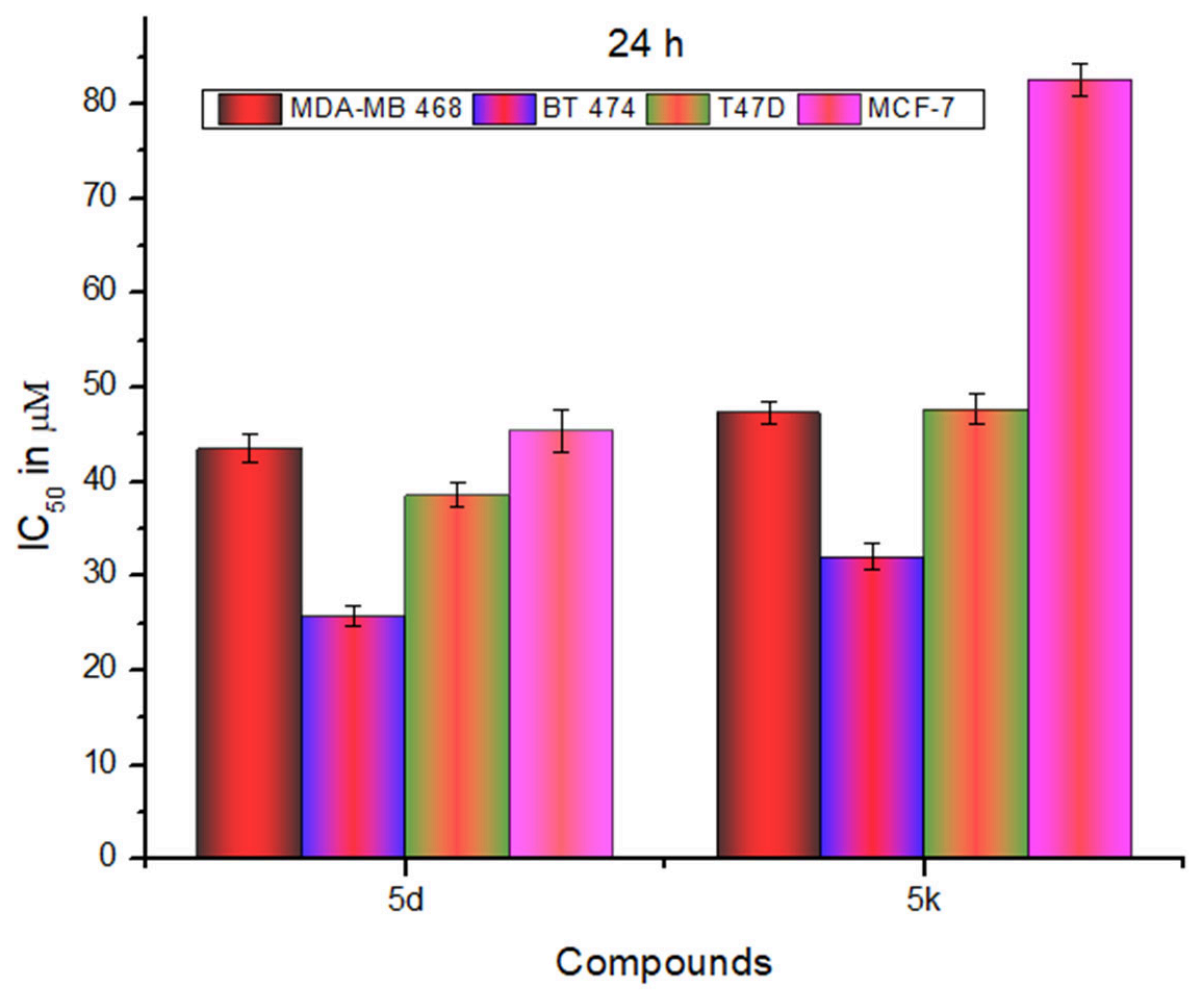
The cytotoxic activity of the compounds was evaluated in different breast cancer cell lines, and the IC_50_ values are presented as means ± SDs from independent experiments.

Compounds **5d** and **5k** exhibited better cytotoxicity against the BT-474 cell line compared with all other cell lines in this study. In particular, the cytotoxic activity against the BT-474 cell line was observed to be IC_50_ = 32.08 ± 1.45 at 24 h for **5d** and IC_50_ = 25.69 ± 1.09 at 24 h for **5k**. On the other hand, **5b** (IC_50_ = 55.2 ± 2.31 μM at 24 h) exhibited moderate activity against the MDA-MB-468 cell line. But, when tested against the triple-positive breast cancer cell line BT-474, **5b** (IC_50_ = 72.6 ± 1.69 at 24 h) exhibited very minimal activity (Figure 1). Compound **5c** showed the lowest activity against the MDA-MB-468 and BT-474 cell lines. The IC_50_ values were found to be 74.18 μM ± 1.29 and 68.58 ± 1.25 μM at 24 h, respectively for the MDA-MB-468 and BT-474 cell lines. The IC_50_ value of compound **5a** (IC_50_ = 96.18 ± 1.46 μM at 24 h), without alkyl substitution on the imidazole nitrogen ring, is high compared with that of the alkyl-substituted compounds **5d** and **5k**. Based on these results, we summarize that there is a favorable reduction in IC_50_ value with an increase in the alkyl chain on the nitrogen of the imidazole ring. Other than the aliphatic substitution, the tolyl, fluorobenzyl, m-phenyl, chlorobenzyl, bromobenzyl and heterocyclic ethyl morpholine substitutions enhanced cytotoxic activity towards the selected cell lines of breast cancer.

The cytotoxicity of the compounds was tested against the normal macrophage cell line, and the results are shown in Table 2. All the active compounds exhibited varying levels of cytotoxic activity against the L929 normal cell line, with IC_50_ values in the range of 36.4 ± 1.01, 30.5 ± 1.31, and 32.29 ± 1.05 μM indicating that the active compounds **5d** and **5k** were destructing the normal cells along with the cancerous cells. As seen in Table 2, both compounds **5a** and **5b** were cytotoxic on normal cells, more than they were antiproliferative on the breast cancer cell lines tested. This has led to a low therapeutic index (poor selectivity to cancer cells). Hence, **5a** and **5b** (8.59 ± 1.69, 6.35 ± 1.22 μM) were not considered good lead candidates for further development. Conversely, compounds with better selectivity towards cancer cells and less toxicity to normal cells were found as more active candidates for further studies. The substituted aromatic and heterocyclic compounds exhibited IC_50_ > 100 μM against the MDA-MB-468, BT-474, T47D, and MCF-7 cell lines. In summary, among all the tested compounds, the compounds with imidazole nitrogen with the longest aliphatic chain were active against the BT474 cell line compared with the unsubstituted imidazole nitrogen and also with the other aromatic substitutions. From Table 2, it can be observed that compounds **5d** and **5k** exhibited effective inhibitory activity against all target cell lines.

#### 2.2.2. Evaluation of the Antiproliferative Potential of Phenanthryl–Imidazole–Pyrrole (**6a**–**6i**) Derivatives in Breast Cancer

**Figure 2:**
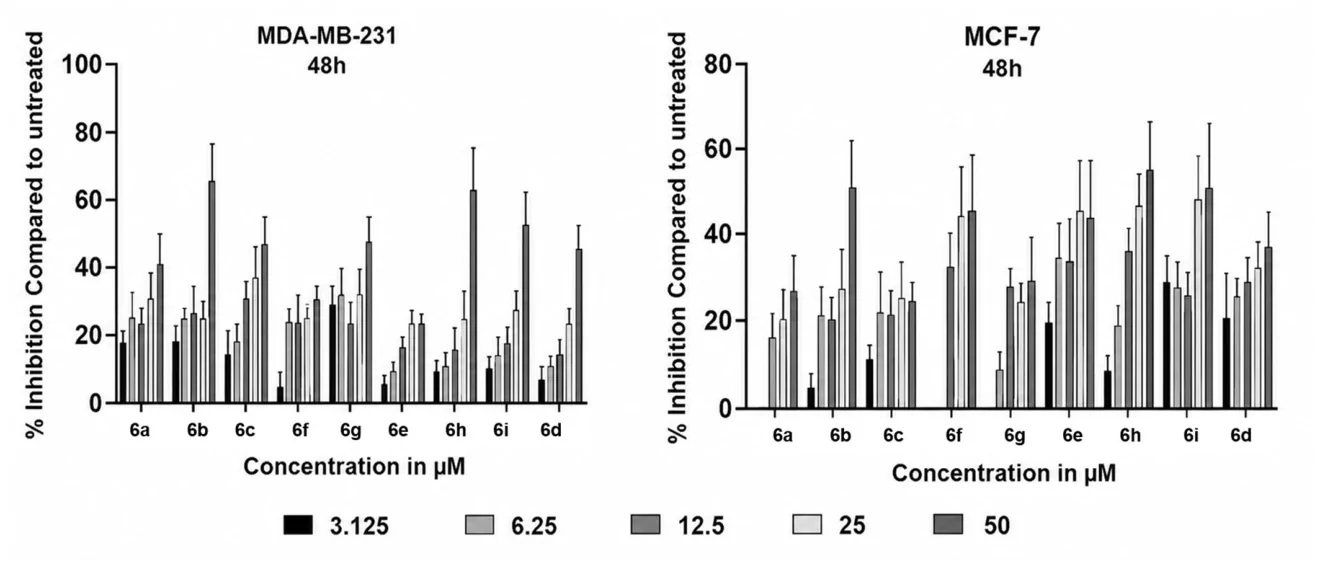
Cytotoxicity of **6a**–**6i** against breast cancer cell lines at 48 h. Compounds **6b** and **6i** more effectively reduced the viability of breast cancer cell lines compared with all other molecules tested.

The second series of compounds were tested against the breast cancer cell lines MDA-MB-231 and MCF-7 (Figure 2). The synthesized compounds exhibited a dose-dependent response in both cell lines. Among the various derivates tested, compound **6b** exhibited better cytotoxicity against MDA-MB-231 cells with 65.64% inhibition at 50 μM, whereas compound **6i** demonstrated higher cytotoxicity against MCF-7 cells, as shown by 54.17% inhibition compared with untreated cells at 50 μM. The variations in efficacy could be attributed to the substitutions and their influence on the receptors. Based on these results, we conclude that **6b** and **6i** have better cytotoxic effects compared with other compounds (Table 3). These findings indicated that structural modifications can influence selectivity and potency against different breast cancer subtypes.

**Figure 3:**
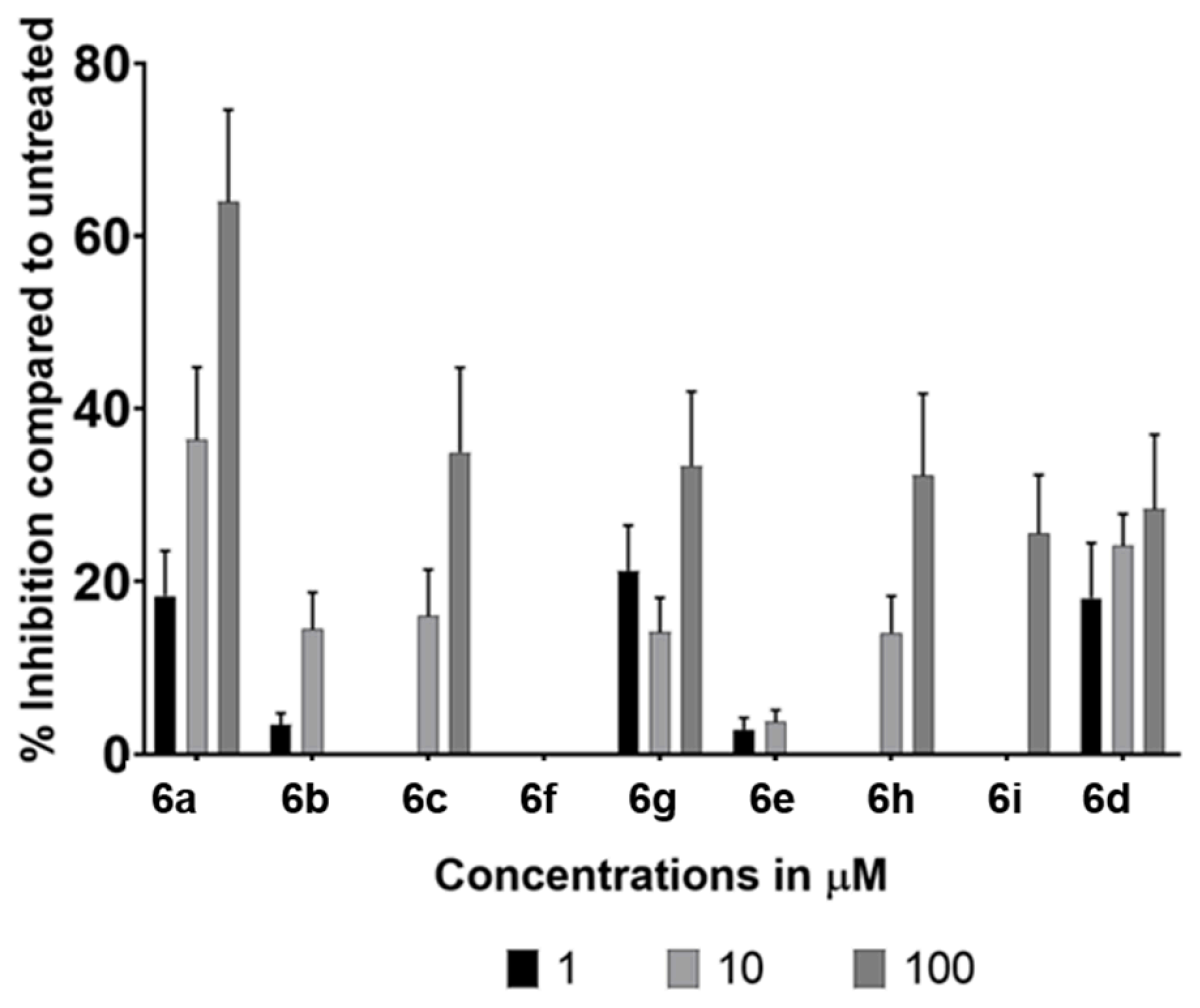
The cytotoxic potential of molecules **6a**–**6i** was tested against the normal human keratinocyte cell line HaCaT using the SRB assay.

To evaluate the selectivity of phenanthryl–imidazole–pyrrole derivatives, the compounds were tested against another normal cell line, i.e., the human keratinocyte cell line HaCaT (Figure 3). Analysis of the cytotoxicity data revealed that compounds **6a**, **6c**, **6g** and **6d** exhibited cytotoxicity even toward HaCaT cells. Compound **6i** was found to be the least toxic among all compounds. Compounds **6b** and **6e** also exhibited lower cytotoxicity compared with the other compounds. At 100 µM concentration, **6b**, **6c**, **6e**, and **6h** precipitated in the media and thus did not show much toxicity against the normal cell line. These findings identified **6b** and **6i** as compounds of interest for further investigation based on their comparatively higher activity against the tested breast cancer cell lines.

#### 2.2.3. Compounds **6b** and **6i** Induced Cancer Cell Death Through G0/G1 Phase Cell Cycle Arrest

**Figure 4:**
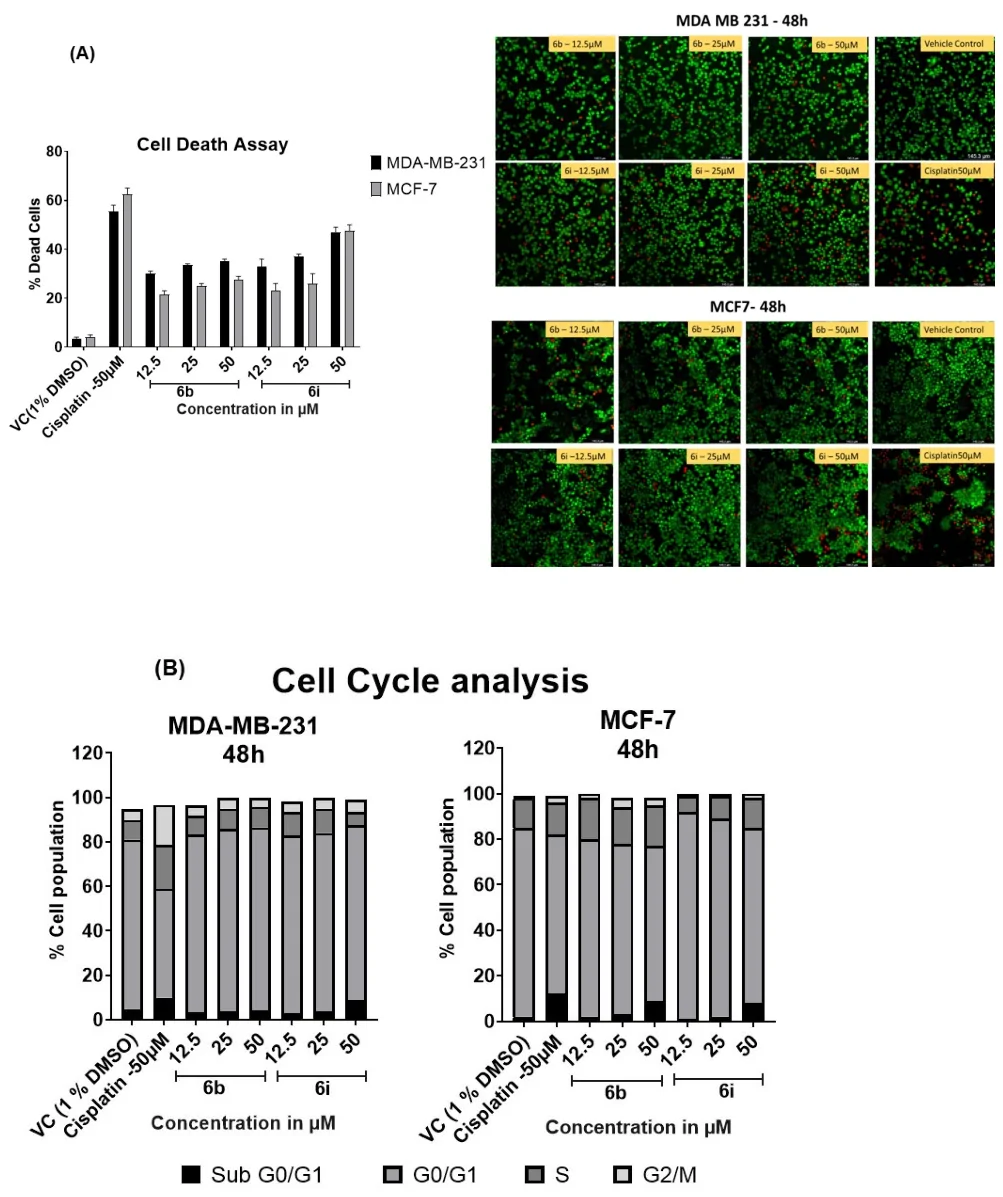
Cell cycle analysis was performed to confirm the cell death induction data. (**A**) Cell death assay (**B**) Cell cycle analysis.

To further determine the mechanistic basis of cytotoxicity due to treatment with **6b** and **6i**, a well-established assay measuring cell death (acridine orange/ethidium bromide (AO/EtBr, staining) was performed on MDA-MB-231 and MCF-7 cells. The cells were treated with increasing concentrations (12.5, 25 and 50 µM) of compounds **6b** and **6i**, and processed for measuring the cell death as detailed in the Methods Section. Analysis of the photomicrographs showed a dose-dependent increase in the number of cells stained red indicating dead cells, compared with green-stained viable cells in the control. Among the two cell lines, MDA-MB-231 exhibited greater sensitivity, as evidenced by an increased number of dead cells compared with MCF-7 (Figure 4A).

Additionally, cell cycle analysis was performed to confirm cell death induction by compounds **6b** and **6i**. Experimentally, the cells were stained with DAPI and the percentages of stained cells were counted to group the cells in the sub-G0-G1, G0/G1, S and G2/M phases. The acquired data show that both **6b** and **6i** could induce cell death, as evidenced by an increase in the sub-G0/G1 population with the increase in concentration of compounds in both cell lines. Furthermore, compounds **6b** and **6i** caused significant cell cycle arrest at the G0/G1 phase, suggesting inhibition of cancer cell proliferation through modulation of cell cycle progression (Figure 4B).

#### 2.2.4. Compounds **6b** and **6i** Induced Cell Death by Increasing Cellular ROS

**Figure 5:**
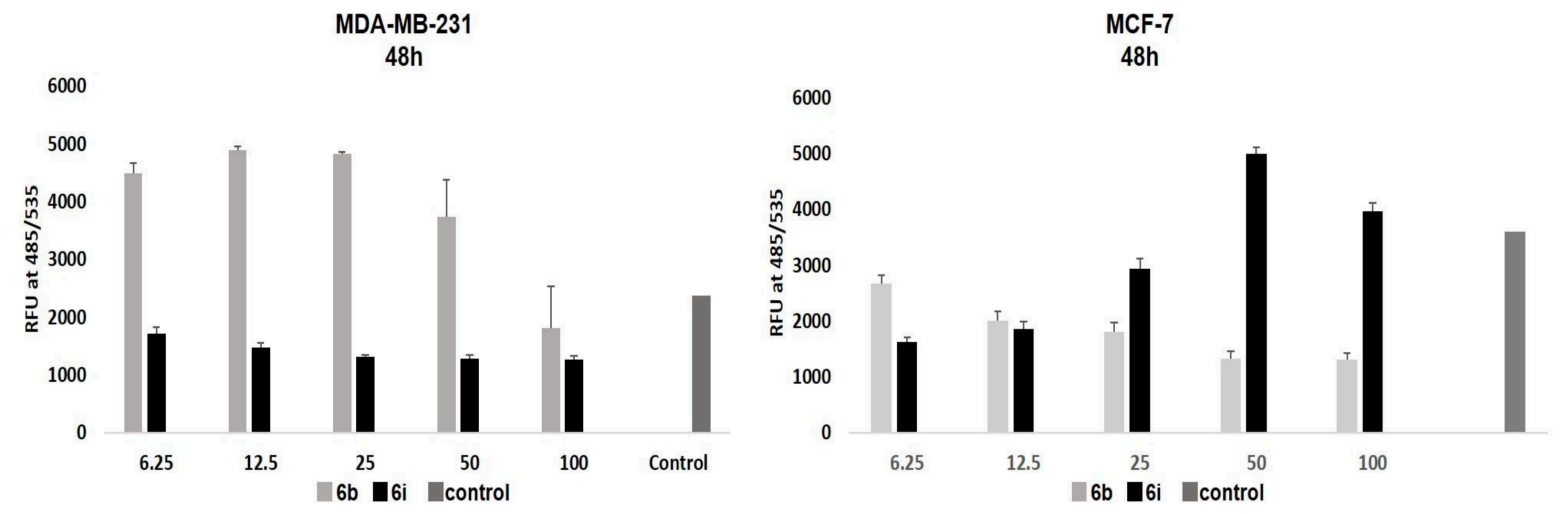
Compounds **6b** and **6i** induced reactive oxygen species (ROS) generation in breast cancer cells. Increased fluorescence intensity indicates elevated ROS production compared with the untreated control.

Induction of ROS is another mechanism by which anticancer agents exhibit cytotoxicity. Pharmacological agents induce intracellular ROS by multiple mechanisms, including the inhibition of enzymes such as Superoxide Dismutase (SOD), Catalase (CAT), Glutathione S-transferases (GSTs) and Peroxidase to promote ROS-generating reactions that include incomplete oxidation of unsaturated fatty acids, glucose, etc. As a result, free radicals accumulate in cells, which ultimately damages biomolecules such as membrane lipids, DNA and proteins and causes massive cell death. Hence, it is important to measure whether the anticancer agents identified in this study also induce ROS in breast cancer cells. The breast cancer cell lines MDA-MB-231 and MCF-7 were treated with active compounds **6b** and **6i** for 48 h, and intracellular ROS were measured by the H2DCFDA protocol as detailed in Materials and Methods Section. Analysis of the data showed that compound **6b** is effective in inducing ROS in the MDA-MB-231 cell line compared with **6i** (Figure 5). In contrast, compound **6i** exhibited better ROS induction in the MCF-7 cell line. Reasons for this differential behavior of **6b** and **6i** are yet to be elucidated. A visible decrease in ROS was observed at 50 µM and 100 µM of **6b** in the MDA-MB-231 cell line, which could be due to extensive cell death at these concentrations. Similarly, a noticeable decrease in ROS was observed with **6i** treatment in the MCF-7 cell line.

### 2.3. In Vivo Studies

Compound **6i** demonstrated significant anticancer activity against breast cancer cells in in vitro studies while exhibiting minimal inhibitory effects on normal cell lines at lower concentrations, indicating favorable selectivity toward cancer cells. Based on these promising results, compound **6i** was selected for further evaluation of its antitumor potential through in vivo studies using an experimental tumor model (Figure S1).

#### 2.3.1. Tumor Reduction Studies

**Figure 6:**
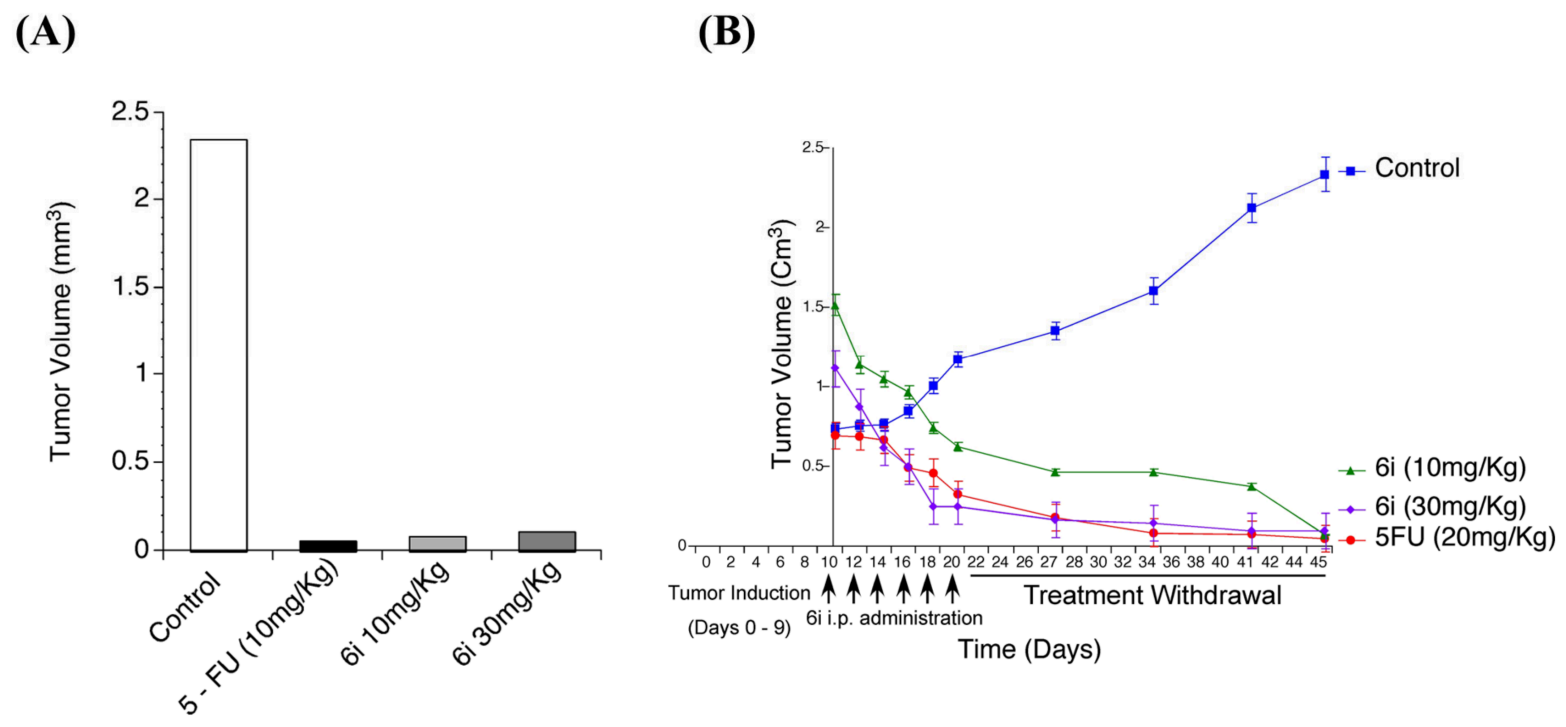
(**A**) Tumor volume on the 32nd day and (**B**) tumor volume kinetics during the treatment period.

Tumor reduction and survival studies were carried out to understand the impact of compound **6i** compared with the standard chemotherapeutic agent 5FU. Intraperitoneal administration of 10 mg/kg and 30 mg/kg of **6i** could effectively retard the growth of EAC tumors in BALB/c mice. Significant tumor reduction was observed even with the standard drug 5FU compared with control mice (Figure 6). The tumor volume is shown in Figure 6B as the mean ± SD of six mice in each group. Slight differences in the starting volume of the tumors between the groups were caused by biological variability at the time of randomization. After the establishment of tumors, mice were randomly distributed to the treatment groups to guarantee similar baseline parameters. Thus, the apparent difference in the starting tumor volume represents the mean of the group and does not influence overall interpretation of antitumor activity.

**Figure 7:**
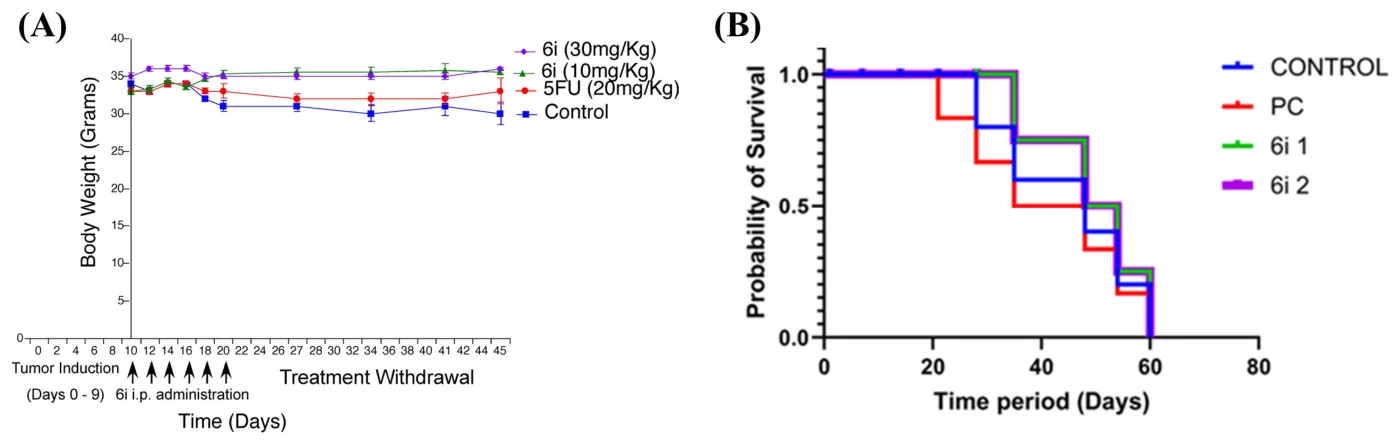
(**A**) Body weight profile of EAC solid tumor-bearing BALB/c mice following treatment and (**B**) Kaplan–Meier survival analysis of EAC solid tumor-bearing BALB/c mice showing the effect of treatment on overall survival.

The Kaplan–Meier curve analysis showed improved survival benefit of compound **6i** and 5FU. The shortest survival was detected in the control and 5FU-administered groups. The lowest concentration of **6i** evidenced to be non-toxic in toxicity assessment. The tumor volume was significantly reduced (*p* < 0.005) in both **6i**-treated groups on the 26th day of the experiment compared with the control. Kaplan–Meier survival analysis established the efficiency of **6i** for survival existence of treated mice when compared with positive control and untreated control tumor-bearing mice. Mice body weight variations were only perceived in the **6i**-administered group compared with 5FU and control (Figure 7).

#### 2.3.2. Histopathological Analysis

**Figure 8:**
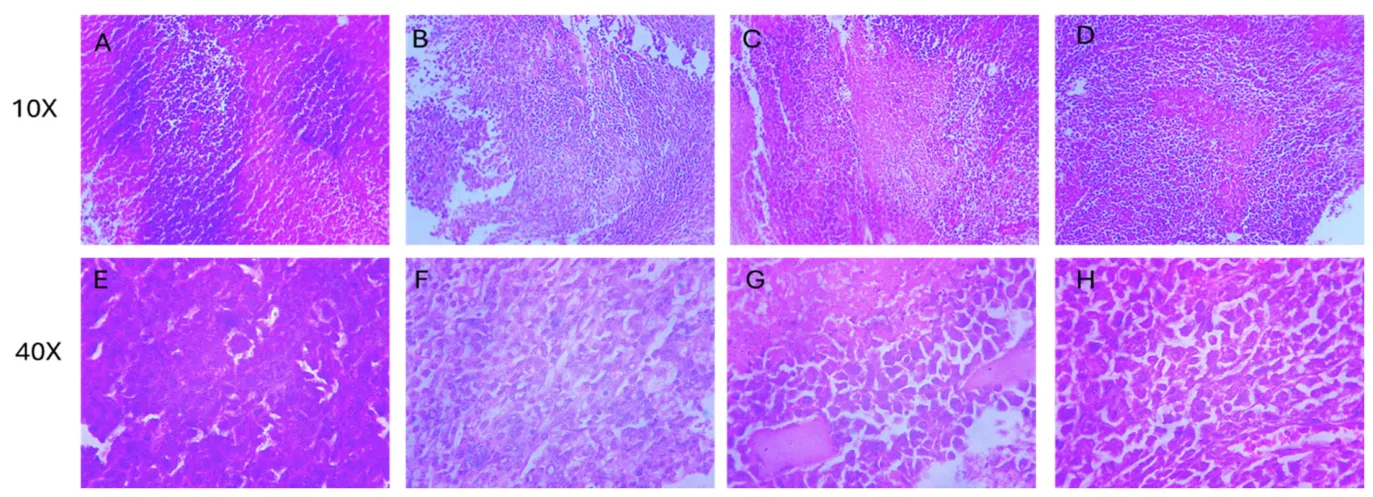
Histopathological examination revealed treatment-associated changes in tumor morphology. H&E staining of tumor in control (**A**,**E**), PC (**B**,**F**), **6i**1-treated (**C**,**G**) and 6i2-treated (**D**,**H**) mice.

**Figure 9:**
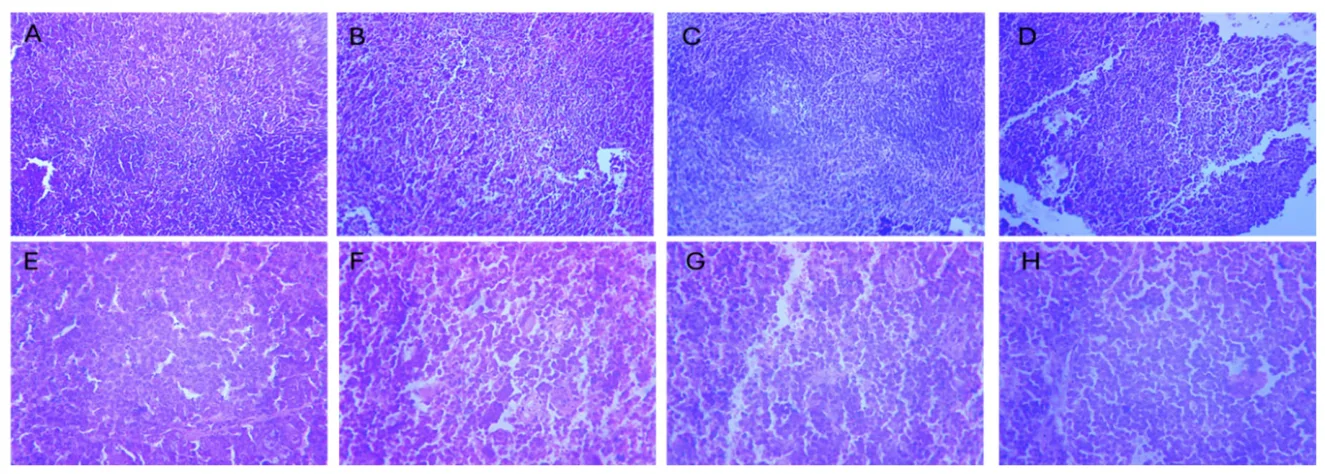
Histopathological examination was performed to assess treatment-related morphological changes in liver tissue. H&E staining of liver in control (**A**,**E**), PC (**B**,**F**), 6i1-treated (**C**,**G**) and 6i2-treated (**D**,**H**) mice. Top row is 10×, and bottom row is 40 × magnification.

Vital organs and tumors were sectioned and examined histopathologically to confirm the safety and antitumor ability of **6i** at the tissue level (Figure S2). The H&E staining of tumor tissue showed the presence of diminished infiltrating neoplasm composed of large polygonal cells with mitotic activity in both the control and 5FU groups along with necrosis (Figure 8 and Figure 9). Mice administered a low dose, i.e., 6i1, showed a response to therapy, as evidenced by necrosis admixed with malignant tumor with large polygonal neoplastic cells with vesicular nucleoli with marked pleomorphism. In the case of the high dose, i.e., the 6i2-administered group, the tumor tissue showed malignant neoplasm composed of sheets and lobular arrangements of large polygonal cells with abundant eosinophilic cytoplasm and vesicular nucleus with prominent nucleoli. Areas of necrosis in the peripheral area of liver tissue in control and 5FU-treated mice were observed. Accumulation of mononuclear inflammatory cells diffusely in the lobules and more predominantly in the periportal areas was also evident in control and 5FU-treated mice.

The sections also showed sinusoids and dilated central vein. Minimal lymphocytic infiltration of tumor cells was also observed. Compound **6i**-administered groups witnessed reduced infiltration, along with normal lobular architecture, and the hepatocytes arranged in cords with cytoplasmic vacuolations. Minimal lymphocytic infiltration was seen around the periportal area in the liver, with no sign of hepatitis at lower concentration but a notable severe-hepatitis condition at higher concentration. Histopathology of the spleen showed normal white pulp and red pulp. White pulp showed lymphoid aggregates forming a germinal center. The penicillar artery showed normal morphology. There was no evidence of malignancy in any group. Analysis of the hematological parameters showed that the RBC count was reduced in PC when compared with the other groups. Total counts (TCs) were high in the control and 6i2-treated groups, indicating an inflammation response. Hemoglobin (Hb) and platelets were found to be normal in 6i1- and 6i2-treated mice compared with the control. The kidney function indicators urea and creatinine tested were also found to be normal in all the groups. But the liver function indicators serum glutaric oxaloacetic transaminase (SGOT) and serum glutamic pyruvate transaminase (SGPT) were altered in the control and 6i2-treated groups, indicating necrosis of the liver, which was also confirmed by the histopathological examination of the liver (Tables S2 and S3).

### 2.4. In Silico Studies

#### 2.4.1. Molecular Docking (**5a**–**6i**) of Selected Agents Targeting Breast Cancer Proteins

A docking study of a given drug–receptor pair provides insights into how strongly the drug binds to its target by calculating the binding affinity, which reflects the stability and favorability of the predicted interaction. A higher negative value of this parameter indicates stronger binding [27]. Docking not only provides key information about the conformation adopted by a ligand within the receptor’s active site but also yields detailed insights into the non-covalent interactions formed between the ligand and the surrounding amino acid residues of the receptor. In this study, the active compound **5d** was docked against the target proteins with PDB IDs 4KZN (human VEGF-A receptor binding domain), 5GPG (co-crystal structure of human FKBP25 (a protein that binds to FK506, the drug rapamycin and the FRB domain of the mTOR kinase), 7NH5 (a co-crystal structure of Akt1 binding to allosteric inhibitors) and 5W9C (human estrogen receptor alpha). Active sites of the proteins were identified through a co-crystalized ligand; their docking scores are given in Table S4. The proteins 5GPG and 7NH5 were found to have the highest docking score. Consequently, the complete ligand series (**5a**–**6i**) was docked against Akt1 (PDB ID: 7NH5), which represents the full-length inactive PH-in conformation of Akt1 containing the experimentally characterized PH–kinase interdomain allosteric binding pocket occupied by the covalent-allosteric inhibitor UC8 [28,29,30]. Details of docking scores tested against the 7NH5 protein are presented in Table 4. The 2D and 3D interactions of the active compounds **5d**, **5k**, **6b** and **6i** are represented in Figure S3.

Among the synthesized derivatives, compounds **6h** and **6i** exhibited the most favorable docking scores (−13.8 and −13.7 kcal/mol, respectively). Analysis of the predicted binding modes revealed that both ligands adopt very similar orientations within the PH–kinase interdomain allosteric pocket of Akt1 and engage residues previously identified as key determinants of allosteric inhibition, including Trp80, Val270, Leu264, Lys268, Tyr272 and Asp292 [28,29]. The extended phenanthrene-containing aromatic scaffold was predicted to form extensive π–π stacking interactions with Trp80 together with multiple hydrophobic contacts involving Val270, Leu264 and neighboring residues, while additional polar interactions with Asp292 further stabilize the predicted binding mode (Figure S3). These interaction patterns are consistent with those reported for structurally diverse Akt1 allosteric inhibitors [28,29,30].

The PH–kinase interdomain pocket of Akt1 has been described as an elongated hydrophobic cleft enriched in aromatic residues that favors extensive van der Waals and π-stacking interactions. Consequently, the large rigid aromatic surface presented by compounds **6h** and **6i** is expected to complement the physicochemical characteristics of this pocket and provide a structural rationale for favorable docking scores. The docking scores reported for compounds **6h** and **6i** lie at the upper end of the range commonly reported for Akt1 allosteric inhibitors, which is consistent with the large, rigid, phenanthrene-containing aromatic scaffold of these compounds and the hydrophobic, aromatic nature of the Akt1 PH–kinase interdomain pocket [31,32,33].

Autodock Vina employs an empirical scoring function based on steric, hydrophobic and hydrogen-bonding interaction terms. Thus, ligands capable of maximizing shape complementarity and extensive van der Waals contacts within such binding pockets may receive particularly favorable docking scores. Accordingly, the docking scores reported here are interpreted comparatively within the present ligand series rather than as quantitative estimates of experimental binding affinity [34,35].

#### 2.4.2. Density Functional Theory

Density Functional Theory (DFT)-based analysis of the drug compounds facilitates the assessment of their electronic stability by calculating key parameters such as the energies of the highest occupied molecular orbital (HOMO), the lowest unoccupied molecular orbital (LUMO), and the HOMO–LUMO energy gap. The energy difference between the HOMO and the LUMO of a compound is directly related to its stability. In this study, the total energy, HOMO and LUMO energy, the HOMO-LUMO energy gap and the dipole moments of compounds **5a**, **5d**, **5k**, **6b**, **6e**, **6f** and **6i** were calculated and are represented in Table S5. The HOMO-LUMO gap of the most active compound, **6i**, was found to be 0.1443 Ha.

#### 2.4.3. Molecular Dynamics Simulation of **6i**

**Figure 10:**
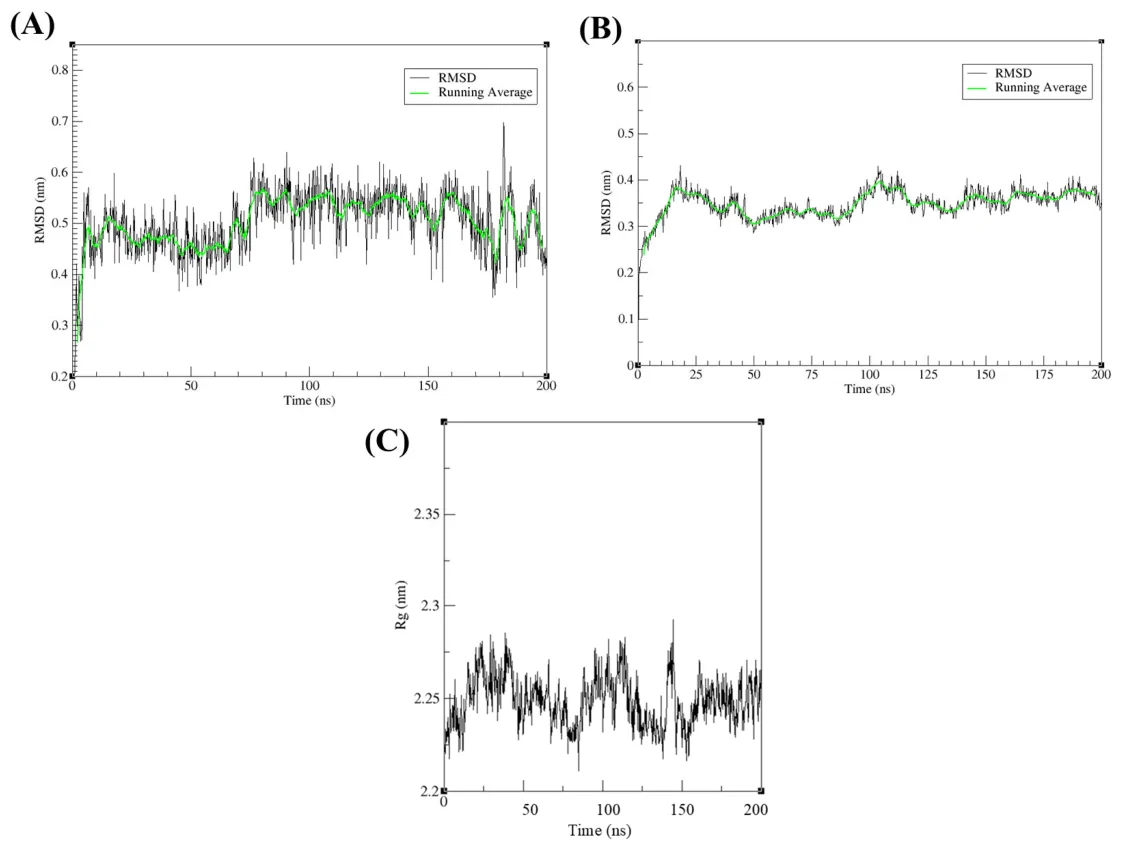
Molecular dynamics simulation analysis of the 7NH5–6i complex. (**A**) Variation in RMSD of **6i** with least-squares fit to the protein. (**B**) Variation in RMSD of the protein backbone. (**C**) Variation in the radius of gyration of the backbone of 7NH5.

Molecular dynamics simulation (MDS) provides dynamic structural information about biomacromolecules, which is essential to understanding the structure–function relationship of the target protein and the nature of protein–ligand interaction. In the present study, the protein–ligand system consisting of compound **6i** and the protein 7NH5 was simulated for 200 ns. The RMSD of the ligand (with least-squares fit to the protein) appears to have quite low values, indicating that the ligand is generally stable within the protein’s active site. Initially, the RMSD increased to about 0.5 nm within the first 10 ns, indicating a rapid adjustment of the ligand within the binding site. After this adjustment, the RMSD stabilized and fluctuated between 0.4 nm and 0.6 nm, with occasional spikes exceeding 0.7 nm, suggesting small intermittent movements of the ligand. The running average depicted in green highlights a gradual stabilization trend, with a consistent average RMSD of around 0.5 nm toward the latter half of the simulation. This RMSD behavior suggests that the ligand initially undergoes conformational adjustments but achieves a relatively stable binding mode thereafter. The spikes in RMSD could indicate transient binding rearrangements or interactions with the protein binding pocket. Overall, the ligand appears to retain its binding position with moderate flexibility, resulting in a stable system. The RMSD plot for the protein backbone shows a stable trend over the course of the simulation. The RMSD increases from approximately 0.2 nm to 0.4 nm during the first 25 ns, representing the initial equilibration of the protein structure (Figure 10A,B). Following this phase, the RMSD fluctuates narrowly around 0.35 nm for the remaining duration of the simulation, which suggests that the protein undergoes minimal structural deviations during the simulation, indicating a high degree of conformational stability. The ratio of a protein’s accessible surface area to the surface area of an ideal sphere with the same volume is known as the protein’s compactness, and the radius of gyration (Rg) of a protein has been suggested to measure its compactness (Figure 10C) [36]. As a protein folds and unfolds throughout a simulation, its radius of gyration changes. A lower Rg indicates a more compact structure, while a higher Rg suggests a more extended or unfolded conformation. The fluctuations observed in Rg are of the order of 0.05 nm, which indicates that the protein is relatively stable throughout the simulation, exhibiting minor structural changes due to a degree of conformational flexibility which it retains. This is consistent with the minor fluctuations around the plateau observed in the RMSD of the protein’s backbone. Throughout the simulation, the NE2 atom of the HIS207 residue was observed to form the most stable hydrogen bond with the N1 atom of the ligand. This hydrogen bond was present for 55.39% of the simulation.

## 3. Discussion

The imidazole–pyrrole compounds’ biological activity was increased by substituting the phenanthryl unit with the phenyl ring. We expected that the cell wall would have been more easily penetrated because the compounds were on the same plane. The results show that distinct aliphatic and aromatic substitutions in an imidazole–pyrrole scaffold produced different impacts on the cytotoxic activity in in vitro analysis. The phenyl–imidazole–pyrrole derivatives were tested against the breast cancer cell lines MDA-MB-468, MDA-MB-231, BT-474, T-47D and MCF-7. Compound **5k**, bearing a heptyl chain on the imidazole nitrogen, showed better inhibition of the breast cancer cell lines. The IC_50_ values were 43.46 ± 1.52, 25.69 ± 1.09, 38.57 ± 1.25, and 45.39 ± 2.34 respectively. When the carbon chain increased from heptyl to octyl on the imidazole nitrogen, compound **5d** showed reasonable inhibition of cancer cell lines. The IC_50_ values were 47.3 ± 1.20, 32.08 ± 1.45, 47.63 ± 1.62, and 82.56 ± 1.63 respectively. In our previous study, imidazole derivatives in which the second position of the imidazole ring was substituted with a pyridine moiety, the fourth and 6i^h^ positions were occupied by phenyl groups, and the N-1 position was functionalized with an octyl chain were synthesized. These compounds were evaluated for their cytotoxic activity against the breast cancer cell lines MDA-MB-468, BT-474, T-47D, and MCF-7. The IC_50_ values obtained were 67.04 ± 1.43, 29.16 ± 2.10 µM, 65.75 ± 1.53 and 82.56 ± 1.21 µM. Based on these results, it was evident that the introduction of an *N*-methyl pyrrole moiety enhances the anticancer activity of the imidazole scaffold. The rest of the compounds in this series showed low activity compared with **5d** and **5k**. Hence, the phenyl group was replaced with phenanthryl to assess the cytotoxicity on the breast cancer lines MDA-MB-231 and MCF-7. The three-component compound **6a** showed an IC_50_ value above 50 µM. The lipophilicity of the compound increased by introducing an ethyl group on the imidazole nitrogen. Compound **6b** showed a better result in inhibiting cancer proliferation, with an IC_50_ value of 41.19 µM. But a similar trend was not observed by increasing the lipophilicity by the increasing carbon chains on the imidazole nitrogen. Compounds **6c**, **6d**, **6e**, and **6g** showed an IC_50_ value above 50 µM. When the substitution on the imidazole nitrogen was performed with aromatic units instead of carbon chains, there was increased inhibition of proliferation of breast cancer cells. Compound **6i**, which had a phenolic group on the imidazole nitrogen, showed an IC_50_ value of 40.37 µM. This compound showed a better activity when compared with all the phenyl–imidazole–pyrrole and phenanthryl–imidazole–pyrrole hybrid compounds. In our previous research work, it was also evident that substituting phenyl with a tolyl group was promising against breast cancer [10]. Compared with previously reported triazole-based imidazolium salts targeting ILK, where the most active compounds exhibited IC_50_ values of 170.58 and 200.84 µg/mL against MCF-7 cells, our synthesized molecules demonstrated comparatively enhanced potency and improved activity profiles. This suggests that the structural modifications introduced in our series significantly contribute to better target interaction and cytotoxic efficacy.

Cytotoxicity was also determined with normal cell lines to determine the therapeutic selectivity of the synthesized compounds. Compounds **5a** and **5b** in the phenyl–imidazole–pyrrole series were highly toxic to L929 mouse fibroblast cells, while **5c**, **5d** and **5k** were moderately toxic to the cells. **5d** and **5k** were strongly antiproliferative against breast cancer cells but also had significant antiproliferative effects against normal L929 cells, which suggests that these compounds exhibited a low level of selectivity between cancerous and normal cells, giving them low therapeutic selectivity. The observation implies that these compounds can be taken up or distributed in a non-specific manner by cells, leading to similar exposure of cancer cells and normal cells. The phenanthryl–imidazole–pyrrole series, however, showed improved safety profiles. HaCaT human keratinocytes were found to be vulnerable to compounds **6a**, **6c**, **6d** and **6g**, with compounds **6b** and **6e** being relatively low in toxicity. Importantly, compounds **6b** and **6i** exhibited comparatively higher antiproliferative activity against the tested breast cancer cell lines while showing lower cytotoxicity toward HaCaT cells at the tested concentrations. These findings warrant further investigation of their cancer cell growth-inhibitory properties.

The predicted ADME properties and drug-likeness profile provide additional computational information regarding the physicochemical and pharmacokinetic characteristics of the compounds (Table S6). The combination of balanced lipophilicity may make the molecule permeable through membranes efficiently, without the risk of high accumulation levels in normal tissues and suitable metabolic stability may minimize the risk of the generation of toxic metabolites. In addition, effective removal is anticipated to reduce the potential for exposure of healthy cells for extended periods of time to reduce the risk of toxicity to normal cells. These results suggest that **6i** and **6b** are the best lead compounds for subsequent preclinical development.

**Figure 11:**
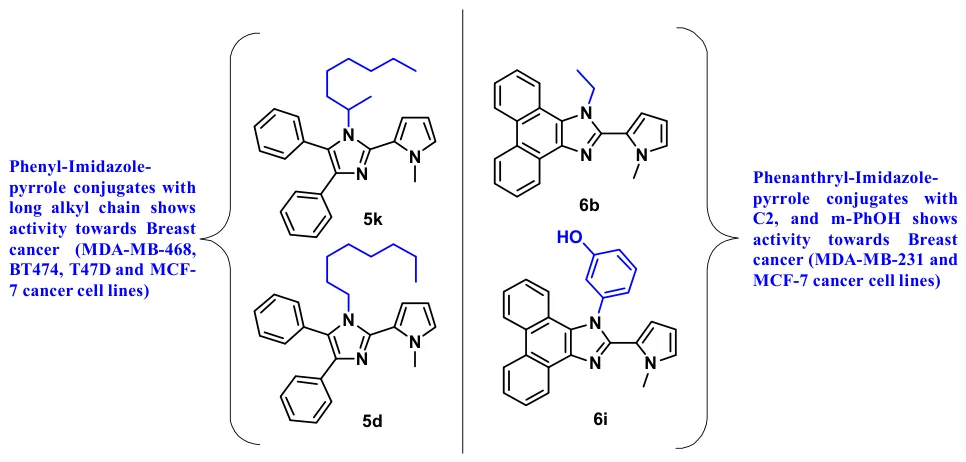
The influence of various structural modifications and substituent patterns on the antiproliferative activity of the imidazole hybrids.

The molecular docking studies revealed that the synthesized imidazole derivatives were able to interact with the ATP-binding pocket of Akt1 by hydrogen bonding, π–π stacking, hydrophobic interaction and van der Waals forces. These interactions resulted in stable protein–ligand complexes and may indicate Akt1 kinase inhibition. Akt1 is a key effector in the PI3K/Akt signaling pathway involved in breast cancer cell growth, survival and apoptosis resistance; therefore, Akt1 inhibition is a plausible molecular explanation for the antiproliferative activity. Most significantly, the phenanthryl–imidazole–pyrrole derivatives showed good binding affinity for Akt1 and were highly cytotoxic to the breast cancer cells, especially **6b** and **6i**. The compounds also showed comparatively lower cytotoxicity toward HaCaT cells at the tested concentrations. Taken together, the docking results and biological evaluation suggest that **6b** and **6i** may interact with the Akt1 target and provide a possible molecular basis for their observed antiproliferative activity. Thus, compounds **6b** and **6i** underwent more advanced in vitro tests, specifically those on cell cycle arrest and apoptosis. Both compounds arrested cells in the G0/G1 stage, thereby preventing the cells from progressing to the M phase. The observed effects of **6b** and **6i** were consistent with their ability to induce G0/G1 cell-cycle arrest and apoptosis, although the magnitude of activity differed from that observed with the standard Cisplatin. The MDA-MB-231 cell line’s apoptosis investigation showed both living and dead cells at varying concentrations of compounds **6b** and **6i** after 48 h. At a higher concentration of 50 µM, both compounds exhibited a higher number of dead cells. Even at larger concentrations, we were unable to observe many dead cells in the MCF-7 cell line. Also, compound **6b** was effective in inducing ROS in the MDA-MB-231 cell line compared with **6i**. In contrast, compound **6i** exhibited better ROS induction in the MCF-7 cell line. These findings suggest a clear structure–activity relationship, with the phenolic group in **6i** and the shorter aliphatic substituents in **6b** appearing to be essential to the strong antiproliferative activity (Figure 11).

Replacing the shorter aliphatic chain with the longest aliphatic chain and other aromatic substituents increased the inhibitory activity (>100 µM). However, among these two compounds, **6i** exhibited favorable biological selectivity by showing lower inhibitory activity toward the normal keratinocyte cell line HaCaT compared with compound **6b**, suggesting reduced cytotoxicity to normal cells. Consistent with this observation, in vivo evaluation of **6i** using the EAC solid tumor model demonstrated significant reduction and improved survival in the 6i-treated groups compared with the standard drug 5FU. Histopathological tumor findings further confirmed therapy-induced necrosis and decreased neoplastic infiltration, particularly at the lower dose, indicating effective antitumor activity with minimal toxicity.

In previously reported triazole-based imidazolium salts targeting ILK, the most active compounds exhibited IC_50_ values of 170.58 and 200.84 µg/mL against MCF-7 cells. Dao et al. evaluated twenty-six synthesized imidazole compounds and found that the most potent molecule in the series showed an IC_50_ of 50 µM against cancer cells, including MDA-MB-231 breast cancer cells. Similarly, Imine-/amine-bearing imidazo [1,2-a]pyrimidine derivatives showed IC_50_ values of 43.4 µM against MCF-7 cells. These comparisons indicate that the present tetra-substituted imidazole derivatives exhibit a comparatively improved potency profile against breast cancer cells relative to several recently reported imidazole-based scaffolds [37,38,39].

## 4. Materials and Methods

### 4.1. Synthesis

We employed the chemicals benzil (Avra Synthesis Pvt. Ltd., Hyderabad, India); 9,10-Phenanthraquinone (Fluka, Buchs, Switzerland); *N*-methyl pyrrole-2-carboxaldehyde (Sigma-Aldrich, St. Louis, MO, USA); ammonium acetate (Sigma-Aldrich, St. Louis, MO, USA); and iodine (Sigma-Aldrich, St. Louis, MO, USA). Without going through any further purification, these substances were used straight out of their sources. The NMR spectral data were obtained using a Bruker Advance TM 400 spectrophotometer (Ettlingen, Germany). Proton (^1^H) and carbon (^13^C) were measured at 400 MHz and 100 MHz, respectively, for NMR spectra. The NMR measurements were conducted using deuterated solvents. For mass spectrometry, a Waters Aquity LCMS instrument with Mass Lynx software 4.2 was employed. Accurate mass measurements were obtained and verified. Infrared (IR) spectra were recorded using a Bruker ALPHA II FT-IR spectrophotometer (Ettlingen, Germany). Using the necessary solvent, column chromatography was performed to purify the chemicals on silica gel (60–120 and 230–400 mesh size).

2-(1-Methyl-1*H*-pyrrol-2-yl)-4,5-diphenyl-1-(p-tolyl)-1*H*-imidazole (**5g**).


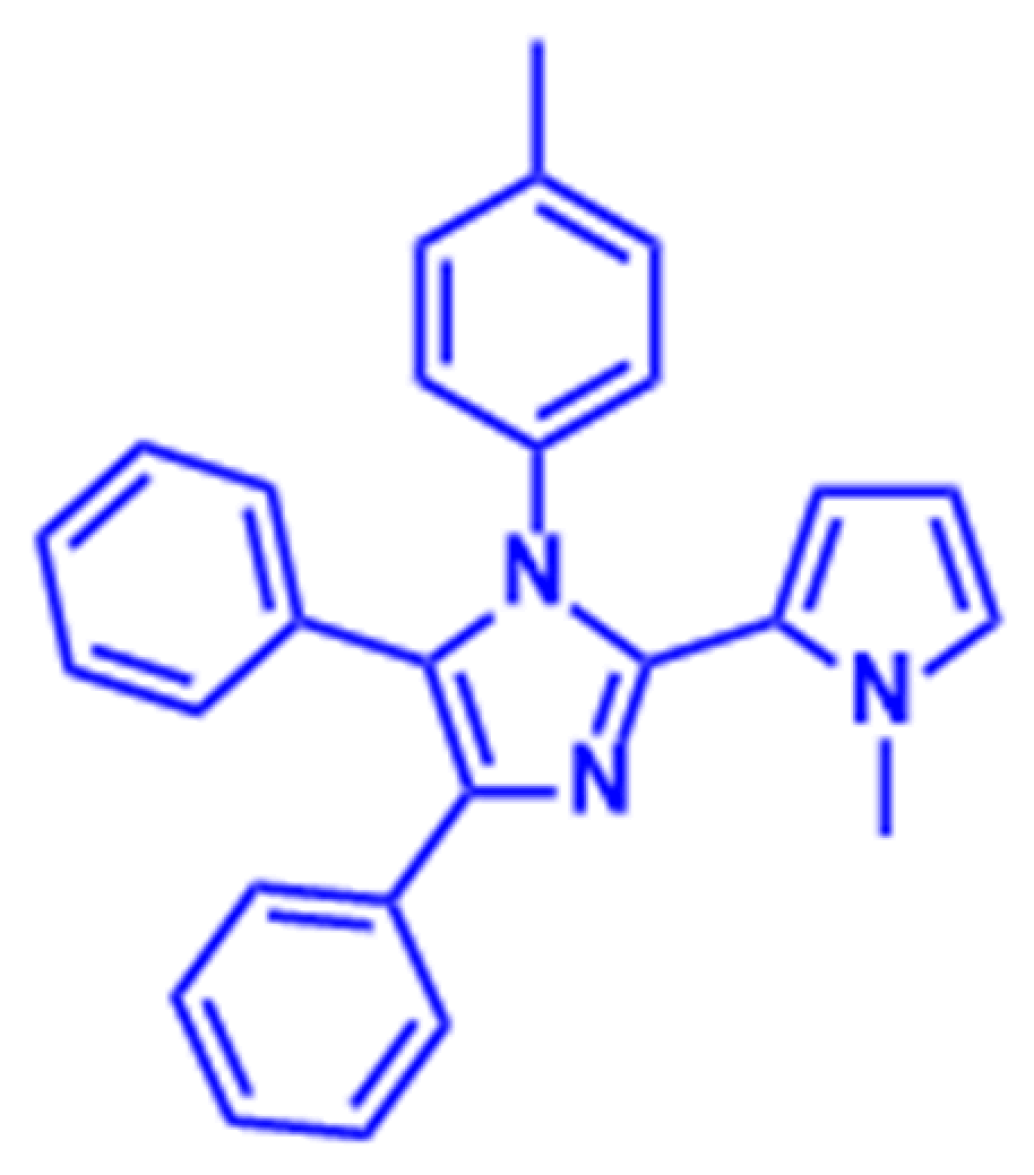


Cream solid; yield: 81%; R_f_ = 0.26; Ethyl acetate: Petroleum ether (1:9). ^1^H NMR (400 MHz, CDCl_3_) δ: 2.23 (s, 3H), 3.99 (s, 3H), 5.55–5.56 (m, 1H), 5.92–5.93 (m, 1H), 6.64–6.65 (t, *J* = 4.0 Hz, 1H), 6.94–6.96 (d, *J* = 8.0 Hz, 2H), 7.05–7.07 (d, *J* = 8.0 Hz, 2H), 7.22–7.24 (m, 8H), 7.59–7.61 (d, *J* = 8.0 Hz, 2H); ^13^C NMR (100 MHz, CDCl_3_) δ; 21.25, 36.44, 94.70, 107.18, 111.51, 124.36 (2C, Ar-m, p-tolyl), 126.07 (2C, Ar-*o*, *p*-tolyl), 126.78 (4C, *Ar-m*, *2 X Ph*),), 128.48, 129.48 (4C, *Ar-o*, *2 X Ph*), 131.15, 135.05, 137.28; FTIR (cm^−1^): 3354, 1593, 1266, 753; LC-MS (ESI + ve): 390.45 (M+H)^+^.

1-(4-Chlorophenyl)-2-(1-methyl-1*H*-pyrrol-2-yl)-4,5-diphenyl-1*H*-imidazole (**5h**).


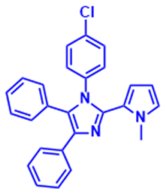


Pale green solid; yield: 62%; R_f_ = 0.26; Ethyl acetate: Petroleum ether (1:9). ^1^H NMR (400 MHz, CDCl_3_) δ: 3.99 (s, 3H), 5.51–5.56 (m, 1H), 5.91–5.95 (m, 1H), 6.66–6.98 (m, 1H), 7.10 (m, 1H), 7.10–7.18 (m, 2H), 7.18–7.27 (m, 2H), 7.35–7.47 (m, 7H), 7.59–7.60 (m, 2H); ^13^C NMR (100 MHz, CDCl_3_) δ: 36.53, 107.88, 108.29, 123.62 (2C, Ar-p, 2 X Ph), 125.39 (4C, Ar-m, 2 X Ph), 127.42 (4C, Ar-o, 2 X Ph), 128.00, 129.01, 132.79, 141.14; FTIR (cm^−1^): 1593, 1268, 756, 668; LC-MS (ESI + ve): 410.44 (M+H)^+^.

1-(4-Bromophenyl)-2-(1-methyl-1*H*-pyrrol-2-yl)-4,5-diphenyl-1*H*-imidazole (**5i**).


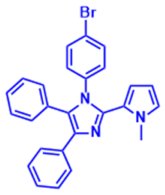


Pale green solid; yield: 65%; R_f_ = 0.26 Ethyl acetate: Petroleum ether (1:9). ^1^H NMR (400 MHz, DMSO-d6) δ: 3.94 (s, 3H), 5.42–5.44 (m, 1H), 5.87–5.89 (m, 1H), 6.84–6.86 (t, *J* = 4.0 Hz, 1H), 7.15–7.19 (m, 1H), 7.23–7.27 (m, 6H), 7.32–7.33 (m, 3H), 7.48–7.50 (m, 2H), 7.53–7.55 (d, *J* = 8.8 Hz, 2H); ^13^C NMR (100 MHz, CDCl_3_) δ: 36.6, 93.5, 107.4, 111.9, 116.0, 116.2, 124.8, 126.7, 127.2 (4C, Ar-m, 2 X Ph), 128.3, 128.6 (4C, Ar-o, 2 X Ph), 129.9 (2C, Ar-m), 130.4, 130.5 (2C, Ar-o), 130.9, 131.2, 131.3; FTIR (cm^−1^): 1594, 1269, 752, 674; LC-MS (ESI + ve): 456.23 (M+2H)^+^.

4-(2-(2-(1-Methyl-*1H*-pyrrol-2-yl)-4,5-diphenyl-1*H*-imidazol-1-yl)ethyl)morpholine (**5j**).


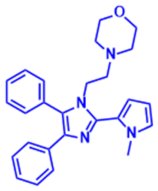


Orange solid; yield: 59%; R_f_ = 0.1; Ethyl acetate: Petroleum ether (1.5:8.5). ^1^H NMR (400 MHz, CDCl_3_) δ: 2.08–2.09 (t, *J* = 4.0 Hz, 3H), 2.23–2.26 (t, *J* = 8.0 Hz, 2H), 3.49–3.51 (t, *J* = 8.0 Hz, 4H), 3.87 (s, 3H), 4.04–4.06 (t, *J* = 8.0 Hz, 2H), 6.19–6.20 (t, *J* = 4.0 Hz, 1H), 6.41–6.42 (m, 1H), 6.77 (m, 1H), 7.11–7.14 (m, 1H), 7.18–7.19 (t, 2H), 7.42–7.52 (m, 7H); ^13^C NMR (100 MHz, CDCl_3_) δ: 35.55, 41.64, 53.65, 58.71, 66.73, 107.57, 110.62, 124.35, 126.13, 126.37 (2C, Ar-p), 127.88 (4C, Ar-m, 2 X Ph), 128.76 (4C, Ar-o, 2 X Ph), 131.18, 134.68, 137.48, 140.98; FTIR (cm^−1^): 1594, 1269, 753; LC-MS (ESI + ve): 413.38 (M+H)^+^.

1-(Heptan-2-yl)-2-(1-methyl-*1H*-pyrrol-2-yl)-4,5-diphenyl-1*H*-imidazole (**5k**).


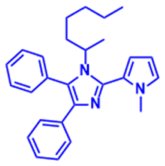


Sticky brown liquid; yield: 60%; R_f_ = 0.17; Ethyl acetate: Petroleum ether (1.5:8.5); ^1^H NMR (400 MHz, CDCl_3_) δ: 0.80–0.84 (t, *J* = 8.0 Hz, 3H), 1.25–1.29 (m, 3H), 1.38–1.43 (m, 8H), 2.58 (s, 3H), 6.49–6.61 (d, 1H), 6.98–7.05 (m, 1H), 7.06–7.09 (m, 1H), 7.13–7.16 (t, *J* = 4 Hz, 2H), 7.29–7.31 (m, 3H), 7.36–7.37 (d, *J* = 4.0 Hz, 2H), 7.43–7.45 (m, 3H); ^13^C NMR (100 MHz, CDCl_3_) δ: 14.04, 22.53, 26.16, 28.56, 28.78, 30.51, 31.57, 44.94, 123.55, 126.47, 126.79, 127.83, 128.13, 128.92, 129.13 (4C, Ar-m, 2 X Ph), 130.38, 130.99 (4C, Ar-o, 2 X Ph), 131.14, 134.25, 136.74, 138.45, 144.38, 149.48, 149.68; FTIR (cm^−1^): 1451, 701; LC-MS (ESI + ve): 398.55 (M+H).

2-(1-Methyl-1*H*-pyrrol-2-yl)-1*H*-phenanthro[9,10-*d*]imidazole (**6a**).


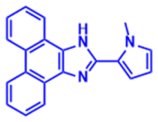


White solid; yield: 50%; R_f_ = 0.25; Ethyl acetate: Petroleum ether (1:9). ^1^H NMR (400 MHz, CDCl_3_) δ: 4.25 (s, 3H), 6.25–6.26 (m, 1H), 6.70- 6.71 (t, *J* = 4.0 Hz, 1H), 6.84 (d, 1H), 7.60–7.64 (m, 5H), 8.73 (bs, 3H), 9.76 (bs, 1H); ^13^C NMR (100 MHz, CDCl_3_) δ: 36.52, 108.04, 109.43, 120.60, 123.47, 124.98, 126.23, 126.83, 127.99, 143.79; FTIR (cm^−1^): 2923.47, 2357.36, 2051, 2015, 1984, 1373, 750, 717, 590, 498, 437; HRMS (ESI + ve): 298.33 [M+H]^+^.

1-Ethyl-2-(1-methyl-1*H*-pyrrol-2-yl)-1*H*-phenanthro[9,10-*d*]imidazole (**6b**).


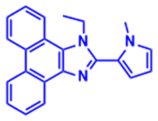


Pale yellow solid; yield: 53%; R_f_ = 0.17; Ethyl acetate: Petroleum ether (1:9). ^1^H NMR (400 MHz, CDCl_3_) δ: 1.66–1.68 (t, *J* = 7.2 Hz, 3H), 3.88 (s, 3H), 4.75- 4.77 (m, 2H), 6.31–6.32 (m, 1H), 6.57–6.58 (m, 1H), 6.90–6.91 (t, *J* = 4.0 Hz, 1H), 7.64–7.73 (m, 4H), 8.36–8.38 (d, *J* = 8.0 Hz, 1H), 8.72–8.74 (d, *J* = 8.0 Hz, 1H), 8.78 (d, *J* = 0.8. Hz, 1H), 8.80 (d,1H), 8.86- 8.88 (d, *J* = 8 Hz, 1H); ^13^C NMR (100 MHz, CDCl_3_) δ: 13.52, 32.42, 46.51, 107.88, 112.28, 120.68, 122.00, 124.63, 125.12, 125.37, 126.71, 127.16, 137.92, 145.18; FTIR (cm^−1^): 3873, 3779, 3605, 3420, 2506, 2331, 2225, 2139, 2031, 1945, 1595, 1024, 739, 500, 438; HRMS (ESI + ve): 326.15 [M+H]^+^.

1-Butyl-2-(1-methyl-1*H*-pyrrol-2-yl)-1*H*-phenanthro[9,10-*d*]imidazole (**6c**).


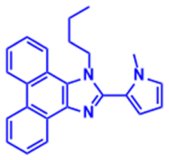


Pale yellow solid; yield: 93%; R_f_ = 0.15; Ethyl acetate: Petroleum ether (1:9); ^1^H NMR (400 MHz, CDCl_3_) δ: 0.89–0.93 (t, *J* = 16.0 Hz, 3H), 1.31–1.39 (m, 2H), 1.93–2.00 (m, 2H), 3.87 (s, 3H), 4.67–4.71 (t, *J* = 16.0 Hz, 2H), 6.30–6.31 (m, 1H), 6.53 (s, 1H), 6.88 (s, 1H), 7.61–7.71 (m, 4H), 8.28- 8.30 (d, *J* = 8.0 Hz, 1H), 8.70- 8.72 (d, *J* = 8.0 Hz, 1H), 8.78–8.80 (d, *J* = 8.0 Hz, 1H), 8.84–8.86 (d, *J* = 8.0 Hz, 1H); ^13^C NMR (100 MHz, CDCl_3_) δ: 13.52, 19.58, 32.42, 35.36, 46.51, 107.88, 112.28, 120.68, 122.00, 124.63, 125.12, 125.37, 126.74, 127.16, 137.92, 145.18; FTIR (cm^−1^): 3769, 3598, 3356, 2926, 2326, 2164, 2043, 1937, 1603, 1452, 1029, 727, 476; HRMS (ESI + ve): 354.48 [M+H]^+^.

1-Hexyl-2-(1-methyl-1*H*-pyrrol-2-yl)-1*H*-phenanthro[9,10-*d*]imidazole (**6d**).


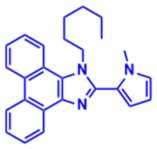


Yellow solid; yield: 52%; R_f_ = 0.13; Ethyl acetate: Petroleum ether (1:9); 1H NMR (400 MHz, CDCl_3_) δ: 0.85–0.89 (t, J = 8.0 Hz, 3H), 1.27–1.29 (m, 4H), 1.95- 2.02 (m, 2H), 3.87 (s, 2H), 4.69 (t, J = 8.0 Hz, 2H), 6.30–6.32 (m, 1H), 6.52–6.53 (m, 1H), 6.88–6.89 (m, 1H), 7.62–7.73 (m, 4H), 8.29 (d, J = 8.0 Hz, 1H), 8.72 (d, J = 8.0 Hz, 1H), 8.77–8.80 (m, 1H), 8.86 (d, J = 8.0 Hz, 1H); ^13^C NMR (100 MHz, CDCl_3_) δ: 13.8, 22.43, 25.99, 30.28, 31.07, 35.30, 44.68, 107.80, 112.17, 120.60, 122.44, 122.94, 124.33, 124.57, 125.05, 127.0*, 137.81, 145.04; FTIR (cm^−1^): 2918, 2160, 1602, 1453, 1358, 1034, 725, 470, 426; HRMS (ESI + ve): 382.21 [M+H]^+^.

2-(1-Methyl-1*H*-pyrrol-2-yl)-1-octyl-1*H*-phenanthro[9,10-*d*]imidazole (**6e**).


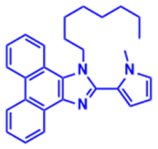


Brown solid; yield: 81%; R_f_ = 0.15; Ethyl acetate: Petroleum ether (1:9). ^1^H NMR (400 MHz, CDCl_3_) δ: 0.86–0.89 (t, *J* = 8.0 Hz, 3H), 1.25–1.28 (m, 9H), 1.95–2.03 (m, 2H), 3.88 (s, 3H), 4.67- 4.71 (t, *J* = 8.0 Hz, 2H), 6.30–6.32 (m, 1H), 6.52–6.53 (m, 1H), 6.88–6.89 (m, 1H), 7.64–7.71 (m, 4H), 8.28- 8.30 (d, *J* = 8.0 Hz, 1H), 8.71–8.73 (d, *J* = 8.0 Hz, 1H), 8.78–8.80 (m, 1H), 8.85–8.87 (m, 1H); ^13^C NMR (100 MHz, CDCl_3_) δ: 13.99, 22.51, 26.28, 28.86, 29.02, 30.28, 31.61, 35.31, 46.68, 107.80, 112.17, 120.61, 122.93, 124.56, 125.05, 125.28, 126.67, 127.08, 137.83, 145.06; FTIR (cm^−1^): 2923, 2853, 1454. 1361, 1053, 751, 720; HRMS (ESI + ve): 410.24 [M+H]^+^.

1-Decyl-2-(1-methyl-1*H*-pyrrol-2-yl)-1*H*-phenanthro[9,10-*d*]imidazole (**6f**).


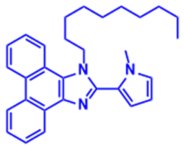


Brown liquid; yield: 88%; R_f_ = 0.16; Ethyl acetate: Petroleum ether (1:9); ^1^H NMR (400 MHz, CDCl_3_) δ: 0.86- 0.90 (t, *J* = 8.0 Hz, 3H), 1.23–1.29 (m, 14H), 1.94–2.01 (m, 2H), 3.87 (s, 3H), 4.65- 4.69 (t, *J* = 8.0 Hz, 2H), 6.29–6.30 (m, 1H), 6.51–6.52 (m, 1H), 6.87–6.88 (m, 1H), 7.61–7.72 (m, 4H), 8.27–8.30 (d, *J* = 12.0 Hz, 1H), 8.70- 8.72 (d, *J* = 8.0 Hz, 1H), 8.77–8.79 (m, 1H), 8.84- 8.86 (d, *J* = 8.0 Hz, 1H); ^13^C NMR (100 MHz, CDCl_3_) δ: 14.01, 22.55, 26.62, 28.89, 29.16, 29.35, 30.28, 31.76, 35.30, 46.68, 107.81, 112.17, 121.92, 123.35, 125.04, 127.36, 127.93, 128.93, 137.84, 145.06; FTIR (cm^−1^): 2921, 2851, 1453, 1359, 717; HRMS (ESI + ve): 438.27 [M+H]^+^.

1-Dodecyl-2-(1-methyl-*1H*-pyrrol-2-yl)-1*H*-phenanthro[9,10-*d*]imidazole (**6g**).


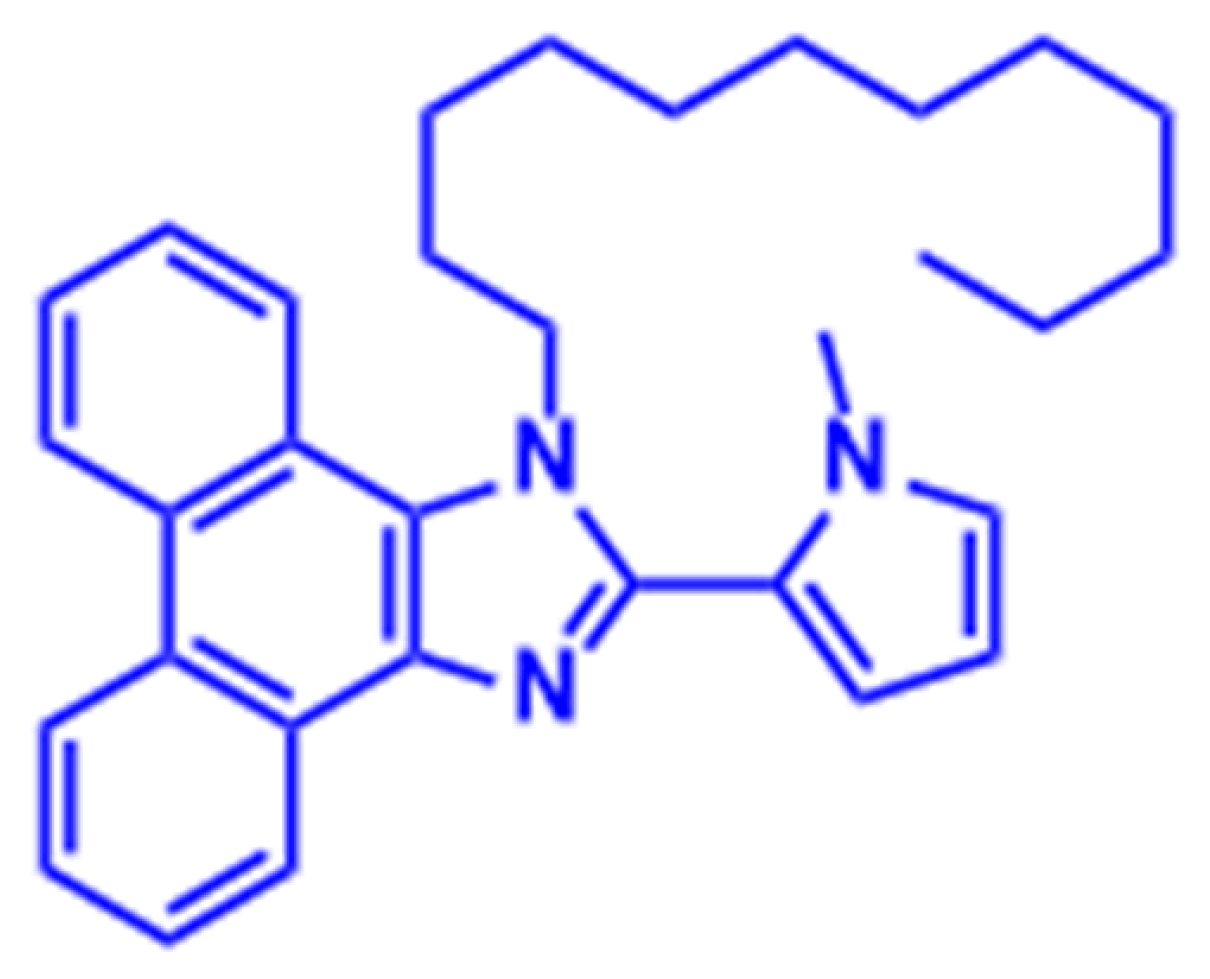


Green solid; yield: 97%; R_f_ = 0.13; Ethyl acetate: Petroleum ether (1:9); ^1^H NMR (400 MHz, CDCl_3_) δ: 0.87–0.89 (t, *J* = 8.0 Hz, 4H), 1.23–1.30 (m, 17H), 1.94–2.01 (m, 2H), 3.61–3.64 (t, *J* = 8.0 Hz, 2H) 3.87 (s, 3H), 4.65- 4.69 (t, *J* = 8.0 Hz, 2H), 6.30- 6.31 (m, 1H), 6.51–6.53 (m, 1H), 6.87–6.89 (m, 1H), 7.61–7.71 (m, 4H), 8.27–8.29 m, 1H), 8.70–8.72 (d, *J* = 8.0 Hz, 1H), 8.78–8.80 (m, 1H), 8.84–8.86 (m, 1H); ^13^C NMR (100 MHz, CDCl_3_) δ: 14.14, 26.37, 29.00, 29.33, 29.47, 29.51, 29.61, 30.38, 31.91, 46.78, 63.05, 107.91, 112.27, 122.02, 123.44, 125.61, 127.45, 128.03, 129.03, 137.93, 145.15; FTIR (cm^−1^): 2919, 2850, 1456, 713; HRMS (ESI + ve): 466.30 [M+H]^+^.

1-(4-Fluorophenyl)-2-(1-methyl-1*H*-pyrrol-2-yl)-1*H*-phenanthro[9,10-*d*]imidazole (**6h**).


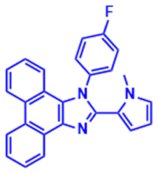


Brown solid; yield: 35%; R_f_ = 0.12; Ethyl acetate: Petroleum ether (1:9). ^1^H NMR (400 MHz, CDCl_3_) δ: 4.19 (s, 3H), 5.64–5.65 (m, 1H), 6.00–6.01 (m, 1H), 6.77–6.78 (t, *J* = 4.0 Hz, 1H), 7.17- 7.19 (d, J = 8.0 Hz, 1H), 7.28–7.38 (m, 3H), 7.51–7.56 (m, 3H), 7.66–7.69 (m, 1H), 7.70–7.78 (m, 1H), 8.72- 8.85 (m, 3H); ^13^C NMR (100 MHz, CDCl_3_) δ: 36.95, 107.38, 112.41, 117.05, 120.47, 122.08, 122.49, 122.73, 123.04, 124.070, 124.65, 125.41, 125.75, 126.26, 127.07, 127.13, 128.07, 128.91 (2C, Ar-m), 130.86 (2C, Ar-o), 134.86, 137.10, 145.08, 161.7, 164.2; FTIR (cm^−1^): 1621, 1500, 1421, 1218, 1062, 807, 729, 478; HRMS (ESI + ve): 392.14 [M+H]^+^.

3-(2-(1-Methyl-1*H*-pyrrol-2-yl)-1*H*-phenanthro[9,10-*d*]imidazol-1-yl)phenol (**6i**).


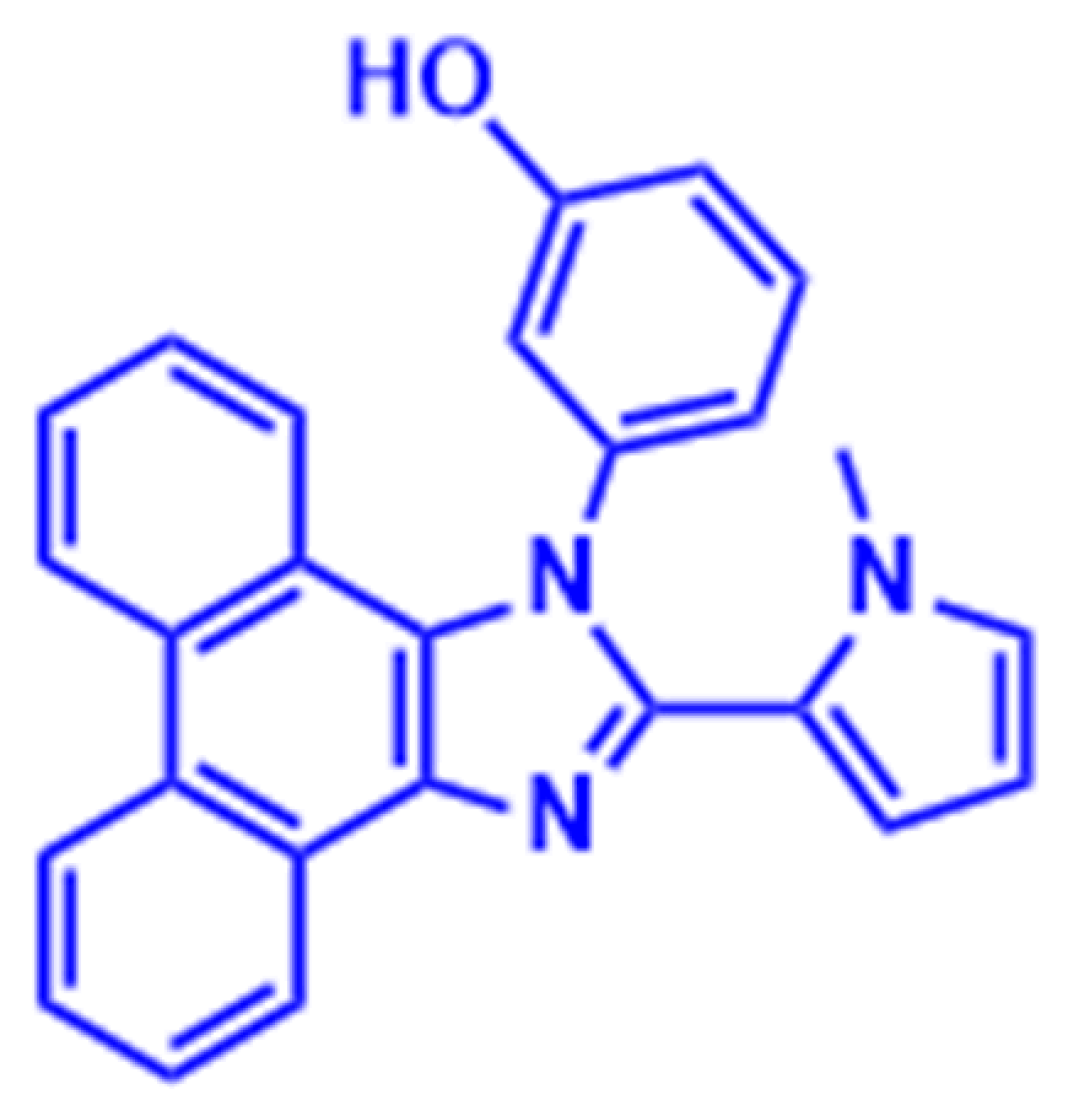


Brown solid; yield: 64%; R_f_ = 0.21; Ethyl acetate: Petroleum ether (1:9). ^1^H NMR (400 MHz, CDCl_3_) δ: 4.03 (s, 3H), 5.48–5.49 (m, 1H), 5.71–5.73 (m, 1H), 6.62 (s, 1H), 6.96–7.00 (m, 3H), 7.14–7.15 (d, *J* = 4.0 Hz, 1H), 7.21–7.22 (t, *J* = 8.0 Hz, 2H), 7.36–7.38 (m, 2H), 7.52–7.56 (m, 1H), 8.16–8.24 (m, 2H), 8.32 (d, *J* = 8.0 Hz, 1H); ^13^C NMR (100 MHz, CDCl_3_) δ: 36.30, 108.36, 112.34, 118.01, 119.98, 120.88, 121.01, 122.11, 122.27, 122.49, 123.73, 124.25, 124.90, 125.19, 125.42, 126.02, 126.13, 127.56, 128.25, 129.26, 131.44, 136.90, 145.34, 154.75; FTIR (cm^−1^): 2354, 1594, 1266; HRMS (ESI + ve): 390.14 [M+H]^+^.

### 4.2. In Vitro Cytotoxicity Studies

The breast cancer cell lines MCF7 (ER+, PR+ and HER2−), MDA-MB-231 (ER−, PR− and HER2−) and MDA-MB-468 (ER−, PR− and HER2−) were procured from the National Center for Cell Science (NCCS) (Pune, Maharashtra, India). The human keratinocyte cell line (HaCaT) was generously provided by Dr. Gopinath, M.S. Principal Scientist, Department of Molecular Nutrition, CSIR-CFTRI, Mysore, Karnataka, India. Dr. Annapoorni Rangarajan, a professor of molecular reproduction, development and genetics at the Indian Institute of Science in Bangalore, India, provided the BT-474 (ER+, PR+ and HER2+) cell line. The mouse fibroblast cell line L-929 was purchased from the ATCC, USA. Dulbecco’s modified Eagle’s medium (DMEM; high glucose: 4.5 g/L), fetal bovine serum (FBS), Glutamax, trypsin, Dulbecco’s phosphate-buffered saline (DPBS) and antimicrobial agents (penicillin and streptomycin) were procured from HiMedia Laboratories (Mumbai, Maharashtra, India). Sulforhodamine B (SRB) was procured from Sigma Aldrich (St. Louis, MO, USA). Cell culture plasticware, T25, T75, 15 mL and 50 mL conical tubes, disposable pipettes, micropipettes tips were procured from Techno Plastic Products (TPP) India, (Pvt) Ltd. (Bengaluru, Karnataka, India). Dimethyl sulfoxide (DMSO) was purchased from Cayman Chemicals (Ann Arbor, Michigan, USA). Anticancer activity of compounds was determined according to the method described by Madhunapantula et al. [40]. Briefly, 1 × 10^4^ cells per well (MDA-MB-231, MDA-MB-468, MCF7, BT-474 and HaCaT) were seeded in a 96-well plate in 100 µL of DMEM supplemented with 10% FBS, 2.6 μM Glutamax and 100 U/mL of Pen-Strep and allowed to grow for 36 h in an incubator (Forma 371 Steri Cycle CO_2_ Incubator, Thermo Fisher Scientific, Waltham, MA, USA) set to 37 °C and 5% CO_2_. Exponentially growing cells were exposed to 1, 10 and 100 µM of the compounds for 48 h. The antiproliferative activity was measured using the SRB assay as described by Skehan et al. [41]. Experimentally, the cells were fixed with 50% TCA (50 µL) by incubating them for 1 h at 4 °C. Later the plates were washed with distilled water and dried. After complete drying, 100s µL of 0.4% SRB was added and incubated for 20 min at room temperature. The excess unbound SRB was washed with 0.1% acetic acid (100 µL× times) and air-dried. The bound SRB was solubilized by adding 100 µL of 100 mM Tris base and mixed well for 5 min. The pink color was read at 540 nm using the Thermo Multiskan GO plate reader.

Percentage inhibition was calculated by using the formula:

% Inhibition = ((OD of untreated cells-OD of treated cells)/OD of untreated cells) × 100

#### 4.2.1. Cell Cycle Analysis

Cell cycle analysis was performed using the NucleoCounter^®^ NC-3000 system (Allerød, Denmark), following the protocol described by Beberok et al. [42]. Briefly, 1×10^6^ cells in 3.0 mL of DMEM supplemented with 10% FBS were seeded into 6-well plates and treated for 48 h with increasing concentrations of compounds **6b** and **6i**. The treatment concentrations were selected based on their respective IC_50_ values. Post-treatment, the cells were trypsinized, washed twice with DPBS, centrifuged at 500× *g* for 10 min, and the supernatant was discarded. Cells were then resuspended in 500 µL of Solution 3 (containing 1.0 µg/mL DAPI and 0.1% Triton X-100 in PBS) and incubated for 5 min at room temperature. A 30 µL aliquot was loaded into the NC-Slide A2 and analyzed using the Cell Cycle-DAPI assay program of the NC-3000 system. The results were interpreted based on the number and intensity of DAPI-stained cells. Histograms were generated, and the percentage of cells in each phase of the cell cycle was determined. Cisplatin (50.0 µM) was used as a positive control.

#### 4.2.2. Determination of Cell Death by Staining with AO and EtBr

Cell death analysis was carried out by staining the untreated and treated cells with ethidium bromide (EtBr) and acridine orange (AO) as per the protocol [43]. Briefly, the cells (0.5 × 10^6^) were centrifuged using a plate rotor at 3000 rpm for 5 min at 4 °C. The medium was removed, and the cells were washed thrice with 100 µL of DPBS. Next, the cells were incubated in 20 μL of DPBS containing 10.0 μg/mL ethidium bromide and 10.0 μg/mL acridine orange for 10 min at room temperature. The dye was removed, and the stained cells were imaged using a laser scanning confocal microscope (Leica Stellaris 5, Wetzlar, Hessen, Germany) using FITC and TRITC channels. The individual images from each filter were merged to obtain a combined image showing green (live) and red (dead) cells [44]. Percentage of dead cell was calculated using the formula below.

% Dead cells = (No. of red cells/Total number of cells) × 100

#### 4.2.3. Determination of Intracellular ROS by H2DCFDA Method

Levels of ROS were estimated according to Anantharaju et al. with minor modifications [45]. MDA-MB-231 and MCF-7 cells (0.5 × 10^4^) were plated in 96-well plates and incubated in a carbon dioxide (maintained at 5%) incubator at 37 °C for 36 h [45]. Next, the growing cells were treated with increasing concentrations of **6b** and **6i** for 48 h. Cells were washed with PBS to remove the traces of medium and incubated with 10 μM 2′,7′-dichlorodihydrouorescein diacetate (H2DCFDA; prepared in PBS). The plates were wrapped with sterile aluminum foil and incubated at 37 °C in the CO_2_ incubator for 30 min. The amount of fluorescence was measured using a multimode plate reader operating at excitation of 485 nm and emission of 535 nm.

### 4.3. In Vivo Studies

**Figure 12:**
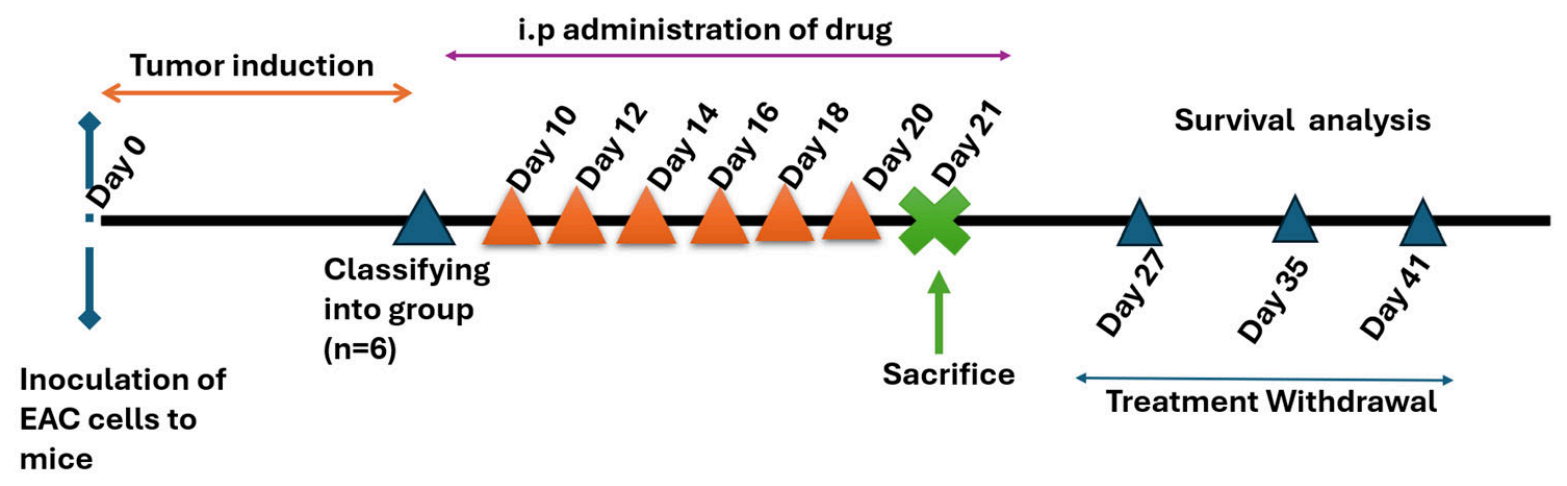
The schematic diagram shows in vivo tumor reduction studies in BALB/c.

BALB/c mice, aged 8–9 weeks and weighing 25 ± 2 g, from an inbred colony were utilized for the in vivo studies. The schematic diagram in Figure 12 shows in vivo tumor reduction studies. The mice were housed in well-ventilated cages with unrestricted access to standard diet and water. The temperature was maintained at 25 ± 2 °C, humidity at 50 ± 5%, and the light–dark cycle was set to 12 h. All animal protocols were thoroughly reviewed and approved by the Institutional Animal Ethics Committee (IAEC) and the Committee for the Purpose of Control and Supervision of Experiments on Animals (CPCSEA) at the Regional Cancer Centre in Trivandrum (Registration No: 657/GO/Re/S/02/CPCSEA).

#### 4.3.1. Induction of Tumor

The murine lymphoid cancer cell line Ehrlich Ascites Carcinoma (EAC) was procured from the Amala Cancer Research Centre, Thrissur, India. EAC cells were maintained by intraperitoneal transplantation at a concentration of 1 × 10^6^ cells/mouse in female BALB/c mice. EAC cells collected from the donor mouse were suspended briefly in sterile isotonic saline, and viable cells were counted (Trypan blue assay) and adjusted to a concentration of about 1 × 10^6^ cells in 100 μL of PBS per site, ensuring that each animal received equal number of EAC cells [46].

#### 4.3.2. Antitumor Activity Against EAC-Induced Solid Tumor

The tumor reduction potency of compound **6i** was tested using the EAC solid tumor model. The experiment involved injecting EAC cells (1 × 10^6^ cells/mouse) subcutaneously into the hind limb of mice (*n* = 6/group) to generate solid tumors. The vehicle control PBS and compounds 5FU (20 mg/kg) and **6i** (10 mg/kg and 30 mg/kg) were administered via i.p. injections every other day for 11 consecutive days (6 doses), beginning from day 9 after the inoculation of EAC cells. At this stage, the mean tumor volume varied from 704.2 mm^3^ (in the positive control group) to 733.9 mm^3^ (in the control group), 1521.0 mm^3^ (in the 10 mg/Kg **6i** treatment group) and 1198.08 mm^3^ (in the 30 mg/Kg **6i** treatment group). Tumor volume (in mm^3^; calculated using the formula 0.52 × Length × (Width)^2^) and overall survival of all mice were monitored. The tumor radii were measured using Vernier calipers at the time of drug administration [47]. The treatment was halted after 11 days, but the measurement of tumor volume continued till day 32. Mice were sacrificed at the end of the study to harvest the tumors and vital organs [47].

#### 4.3.3. Determination of Hematological and Biochemical Parameters

The body weight of mice was investigated throughout the study period. On the day of dissection, blood was collected in EDTA vacutainers and citrate-coated vacutainers for analyzing their hematological parameters.

#### 4.3.4. Morphological and Histological Examination

Liver, spleen, kidney, heart, and tumor specimens from each mouse were preserved in 10% formalin solution [48]. These samples were dehydrated using a carefully graded alcohol series. Following this, they were delicately treated with xylene and then embedded in paraffin blocks. Subsequently, sections measuring five microns in thickness were precisely cut and stained with hematoxylin and eosin (H&E). These stained sections were then subjected to thorough examination and investigation as detailed in the following sections. The liver specimens were assessed for the severity of degeneration and necrosis by the following grading system: 0 for no hepatocyte necrosis or degeneration, 1 for focal necrosis or degeneration (mild), 2 for multifocal necrosis or degeneration of hepatocytes (moderate), and 3 for locally extensive or diffuse necrosis or degeneration of hepatocytes (severe). The excised tumors were each halved along the longitudinal mid-line, which is the longest dimension of the tumor, and the interior sections were photographed using an optical microscope. Some tumor tissue samples were sent to the Origin Laboratory Coimbatore for H&E staining and slide preparation. The stained slides were then digitally scanned, and the images were enhanced using computer software for histological examination. For the H&E-stained slides of tumor specimens, the assessment included: grading the severity and percentage of necrosis based on previous reports and calculating the H score accordingly, counting mitotic tumor cells in 10 randomly selected microscopic fields (equivalent to at least 1000 tumor cells) under high magnification (400×), and counting apoptotic tumor cells in 10 randomly selected microscopic fields under the same magnification. The mitotic index (MI) and apoptotic index (AI) were expressed as percentages by dividing the total number of mitotic cells and apoptotic bodies by the total number of intact tumor cells.

### 4.4. In Silico Studies

#### 4.4.1. Molecular Docking

Molecular docking was conducted using Autodock Vina 1.2.5. Protein structures were prepared for docking through the Dock Prep module in UCSF Chimera (version 1.17.3), which involved the addition of polar hydrogens, completion of incomplete side chains utilizing the Dunbrack 2010 rotamer library, and assignment of Gasteiger charges. Analysis and visualization of predicted protein–ligand interactions were carried out using BIOVIA Discovery Studio Visualizer [49].

#### 4.4.2. Density Functional Theory Analysis

Drug molecule structures initially present in the MDL Molfile format were converted into the Cartesian (XYZ) file format using Open Babel 3.1.1. Geometry optimization was performed using ORCA 5.0.4 with the B3LYP functional and def2-TZVP basis set [50]. Grimme’s D4 dispersion correction was applied to account for dispersive interactions. Single-point energy calculations were conducted on the optimized structures using the same DFT functional and basis set. Finally, the energies and structures of the highest occupied molecular orbital (HOMO) and the lowest unoccupied molecular orbital (LUMO) for each drug molecule were determined using Avogadro 1.2.0.

#### 4.4.3. Molecular Dynamics Simulation

Molecular dynamics simulations were performed on the best-docked pose of the protein–ligand complex using GROMACS to investigate the structural changes within the protein that the ligand may have induced. The topology of the protein was generated using the CHARMM36 all-atom force field, while the topology and parameters of the ligand were generated using the CHARMM General Force Field (CGenFF) [36]. The complex was positioned within a simple cubic box, ensuring that the protein maintained a minimum distance of 1 nm from the box’s edge. The complex was subsequently solvated with TIP3P water molecules, incorporating additional Na+ ions to neutralize the net negative charge of the protein. Additional Na^+^ and Cl^−^ ions were incorporated to establish a 0.1 M concentration of NaCl within the system. According to the GROMACS protocol, the solvated system underwent minimization utilizing the CHARMM force field. The system was subsequently simulated under an NVT ensemble for 200 ps to ensure temperature stabilization at 310 K, utilizing the V-Rescale thermostat while applying restraints to the heavy atoms of the system. A subsequent 200 ps simulation was conducted under the NPT ensemble to stabilize the pressure at 1 bar, employing the C-Rescale barostat while maintaining restraints on the heavy atoms of the system. Subsequently, the constraints on the heavy atoms were lifted, and a production run of the molecular dynamic simulation was conducted for 200 ns. The LINCS algorithm was employed to restrict bonds to hydrogen atoms. The Parrinello–Rahman barostat was employed to sustain pressure at one bar, while the V-Rescale thermostat was utilized to regulate the temperature at 310 K. Coordinates and energies were recorded every 200 ps, accompanied by an update to the log file. The analysis of hydrogen-bonding interactions was performed utilizing the VMD plugin, and H Bonds version 1.2. VMD was utilized for the visualization of the trajectory and for the creation of movies. The plots were created utilizing XMGrace.

### 4.5. Statistical Evaluation

The results are presented as means ± standard errors of the mean. Multiple comparisons were made using the post hoc parametric test following a significant analysis of variance (ANOVA), utilizing the computer-based fitting program GraphPad Prism 8.4.2. A *p*-value < 0.05 was considered statistically significant.

## 5. Conclusions

The imidazole–pyrrole hybrid synthesis method for novel anticancer drugs was successful, and the results are promising. The synthesized compounds exhibited varying levels of antiproliferative activity against the tested breast cancer cell lines, with compounds **5d**, **5k**, **6b**, and **6i** showing comparatively higher activity within their respective series. The phenyl–imidazole–pyrrole derivatives with the longest aliphatic chain, **5d** and **5k**, exhibited good antitumor activity in the phenyl–imidazole–pyrrole series. Among the phenanthryl–imidazole–pyrrole derivatives, compounds **6b** and **6i** exhibited good-to-moderate results, and these compounds exhibited the lowest toxicity towards normal cells (HaCaT cell line). Compounds **6b** and **6i** were tested in apoptosis and cell cycle arrest studies. Compared with compound **6b**, **6i** showed a greater number of dead cells in both the MDA-MB-231 and MCF-7 cell lines at its higher concentration. In summary, we conclude that apoptosis can be induced by substituting the imidazole nitrogen with an *o*-phenolic group as well as an aliphatic chain. The cells were unable to reach the M phase as both compounds inhibited cell development at the G0/G1 stage. Compounds **6b** and **6i** exhibited moderate effect on apoptosis and cell cycle arrest at their lower concentration. The in-silico studies provided useful insights into the potential molecular interactions of the compounds with the Akt1 target. The in vivo findings showed that compound **6i** reduced tumor growth in the EAC solid tumor mouse model and increased the survival of tumor-bearing mice compared with the respective control groups. These findings support the potential of **6i** for further investigation and structural optimization.

## Figures and Tables

**Table 1:** The various catalysts used to synthesize compounds **5a** and **6a**.

Code/Structure		Catalyst Used [Time (h)/Yield (%)]		
		FeCl_3_ (5 mol%)	I_2_ (5 mol%)	ZrOCl_2_·8H_2_O (10 mol%)
**5a**	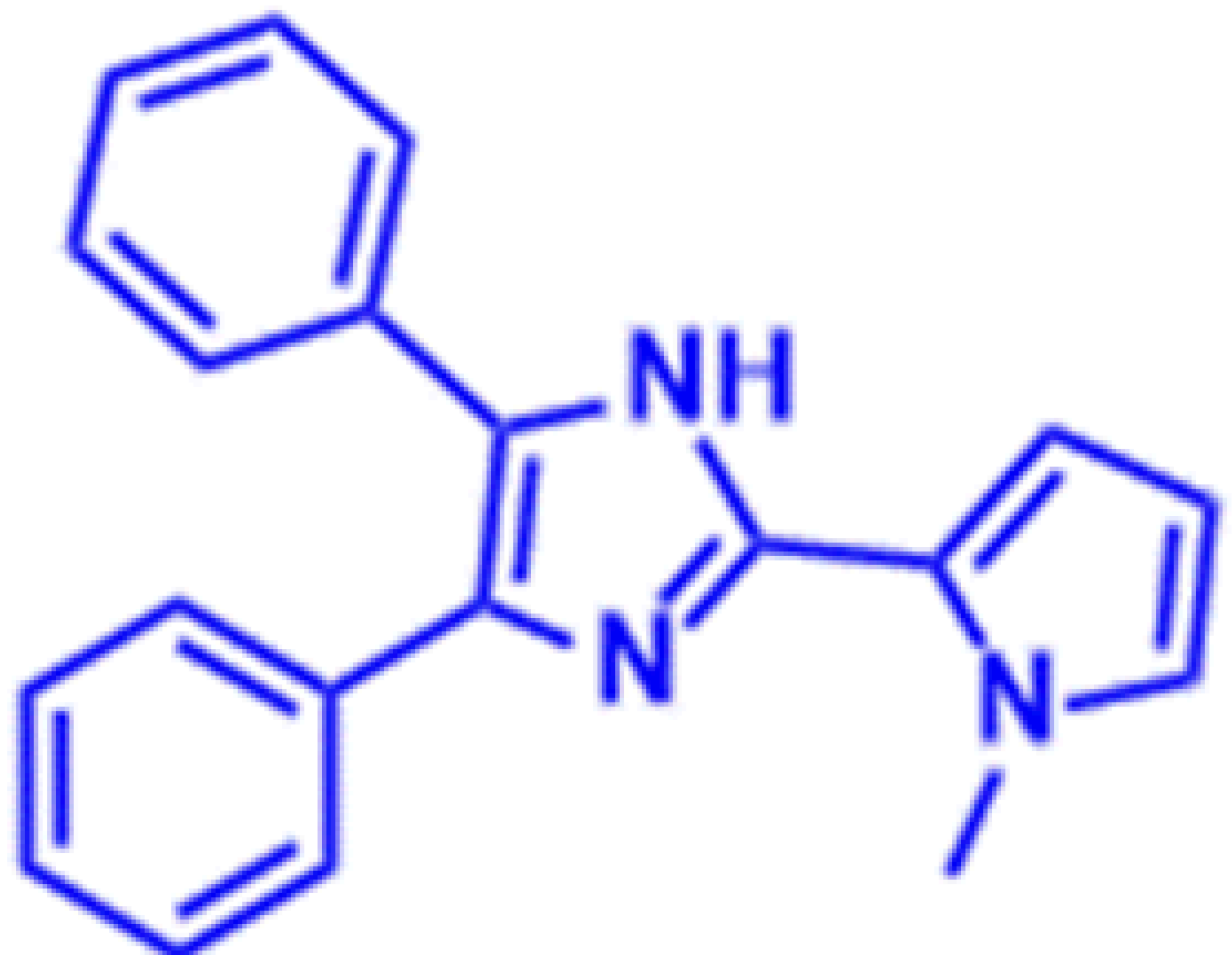	8/42	5.5/62	9.5/35
**6a**	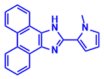	10.5/41	6.5/60	12/23

**Table 2:** In vitro cytotoxicity of **5a**–**5k** against various BC cell lines at 24 h.

Compound	IC_50_ (µM) after 24 h				
	Breast Cancer Cell Lines				Normal Cell Line
	MDA-MB-468	BT-474	T-47D	MCF-7	L929
**5a**	>100	96.18 ± 1.46	>100	>100	8.59 ± 1.69
**5b**	55.2 ± 2.31	72.6 ± 1.69	>100	>100	6.35 ± 1.22
**5c**	74.18 ± 1.69	68.58 ± 1.25	>100	>100	36.4 ± 1.01
**5d**	47.3 ± 1.20	32.08 ± 1.45	47.63 ± 1.62	82.56 ± 1.63	30.5 ± 1.31
**5e**	>100	>100	>100	>100	22.22 ± 1.58
**5f**	>100	>100	>100	>100	24.71 ± 1.67
**5g**	>100	>100	>100	>100	6.63 ± 1.81
**5h**	>100	>100	>100	>100	94.33 ± 1.79
**5i**	>100	>100	>100	>100	84.43 ± 1.47
**5j**	>100	>100	>100	>100	96.8 ± 1.44
**5k**	43.46 ± 1.52	25.69 ± 1.09	38.57 ± 1.25	45.39 ± 2.34	32.29 ± 1.05

**Table 3:** IC_50_ values of compounds **6a**–**6i** in MDA-MB-231 and MCF-7 cell lines at 48 h.

Compound	IC_50_ (µM) at 48 h	
	MDA-MB-231	MCF-7
**6a**	>50	>50
**6b**	41.19	>50
**6c**	>50	>50
**6d**	51.62	>50
**6e**	>50	>50
**6f**	>50	28.97
**6g**	>50	>50
**6h**	>50	>50
**6i**	40.37	>50
**Cisplatin**	38	0.65

**Table 4:** Binding energy of all the synthesized compounds against the 7NH5 protein.

Compound	Binding Energy (Kcal/mol)	Compound	Binding Energy (Kcal/mol)
**5a**	−10.3	**5k**	−9.7
**5b**	−10.2	**6a**	−11.6
**5c**	−10.0	**6b**	−11.9
**5d**	−9.5	**6c**	−11.5
**5e**	−12.1	**6d**	−11.2
**5f**	−11.2	**6e**	−11.1
**5g**	−11.6	**6f**	−10.8
**5h**	−11.5	**6g**	−10.8
**5i**	−11.5	**6h**	−13.8
**5j**	−10.3	**6i**	−13.7

## Data Availability

Data are contained within the article and Supporting Information.

## Supplementary Materials

The following supporting information can be downloaded at: https://www.mdpi.com/article/10.3390/molecules31203722/s1, Scheme S1: Iodine catalysed reaction mechanism of (A) 3 component reaction; (B) 4 component reaction; Table S1. Physical parameters of **5g**–**6i**; Table S2: Tumor and haematological characteristics in mice injected with EAC followed by **6i** administration for 14th days and dissected on 16th day; Table S3: Effect of **6i** extract on Balb/c mice’s biochemical parameters; Table S4: Binding energy of molecule **5d** against various breast cancer proteins; Table S5: DFT calculation for activity molecules; Table S6: In silico analysis of the physicochemical and pharmacokinetic properties of imidazole conjugates (**5a**–**6i**); Figure S1: Photographic representation of BALB/c mice with solid tumour bearing and administrated compounds i.p (A,E) control group (B,F) Positive control group (5 FU), (C,G) 6i1 (10mg/kg) and (D,H) 6i2 (30mg/kg); Figure S2: Organs such as Heart, Liver, spleen and kidneys of (A) Control, (B) PC, (C) 6i1 and (D) 6i2 treated in Balb/c mice with solid EAC tumour bearing models; Figure S3: 2D (a,c,e,g) and 3D (b,d,f,h) structures of Compound **5d**, **5k**, **6b**, **6i** and **6h** with 7NH5 receptors.
