# Supplementary material for: Rationally Designed Phenyl- and Phenanthryl-Based Imidazole Hybrids for the Treatment of Breast Cancer—A Structure–Activity Relationship Study
Source: Molecules. 2026 Oct 16;31(20):3722. doi: 10.3390/molecules31203722 (PMC13707853; doi:10.3390/molecules31203722)
Supplement: Supplementary file 1 [file molecules-31-03722-s001.zip › molecules-4562601-supplementary.pdf]

Supporting Information

Rationally Designed Phenyl- and Phenanthryl-Based Imidazole Hybrids for the Treatment of Breast Cancer—A Structure–Activity Relationship Study

Bharathi Hassan Ganesh [1,2,†], Preethi G. Anantharaju [3,4,†], Amritha Sukumaran [1,2], Carmelo Drago [5], Sarthak Mohanty [1], Himabindu Padinjarathil [1,2], SubbaRao V. Madhunapantula [3,*] and Prasanna Ramani [1,2,*]

[1] Dhanvanthri Laboratory, Department of Chemistry, Amrita School of Physical Sciences, Amrita Vishwa Vidyapeetham, Coimbatore 641112, India; hg_bharathi@cb.students.amrita.edu (B.H.G.); s_amritha@cb.students.amrita.edu (A.S.); sarthakmohanty997@gmail.com (S.M.); p_himabindu@cb.students.amrita.edu (H.P.)

[2] Center of Excellence in Advanced Materials and Green Technologies (CoE–AMGT), Amrita School of Engineering, Amrita Vishwa Vidyapeetham, Coimbatore 641112, India

[3] Center of Excellence in Molecular Biology and Regenerative Medicine (CEMR), Department of Biochemistry, JSS Medical College, JSS Academy of Higher Education & Research, Mysore-570015, India; preethijinesh@gmail.com

[4] Center of Excellence in Cell Biology & Proteomics, College of Pharmaceutical Sciences, School of Health Science, Dayananda Sagar University, Bangalore South- 562112, India

[5] Institute of Biomolecular Chemistry, CNR, Via Paolo Gaifami 18, Catania-95126, Italy; carmelo.drago@cnr.it

* Correspondence: mvsstsubbarao@jssuni.edu.in (S.V.M.); r_prasanna1@cb.amrita.edu (P.R.)

† These authors contributed equally to this work.

Contents

Characterisation of 5g 3

Characterisation of 5h 6

Characterisation of 5i 9

Characterisation of 5j 12

Characterisation of 5k 15

Characterisation of 6a 18

Characterisation of 6b 21

Characterisation of 6c 24

Characterisation of 6d 27

Characterisation of 6e 30

Characterisation of 6f 33

Characterisation of 6g 36

Characterisation of 6h 39

Characterisation of 6i 42

Reaction mechanism 45

Physical parameters of the synthesized compounds 45

In vivo studies 46

In silico studies 49

Characterisation of 5g

2-(1-Methyl-1*H*-pyrrol-2-yl)-4,5-diphenyl-1-(*p*-tolyl)-*1H*-imidazole

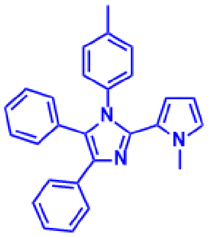

^{1}H NMR

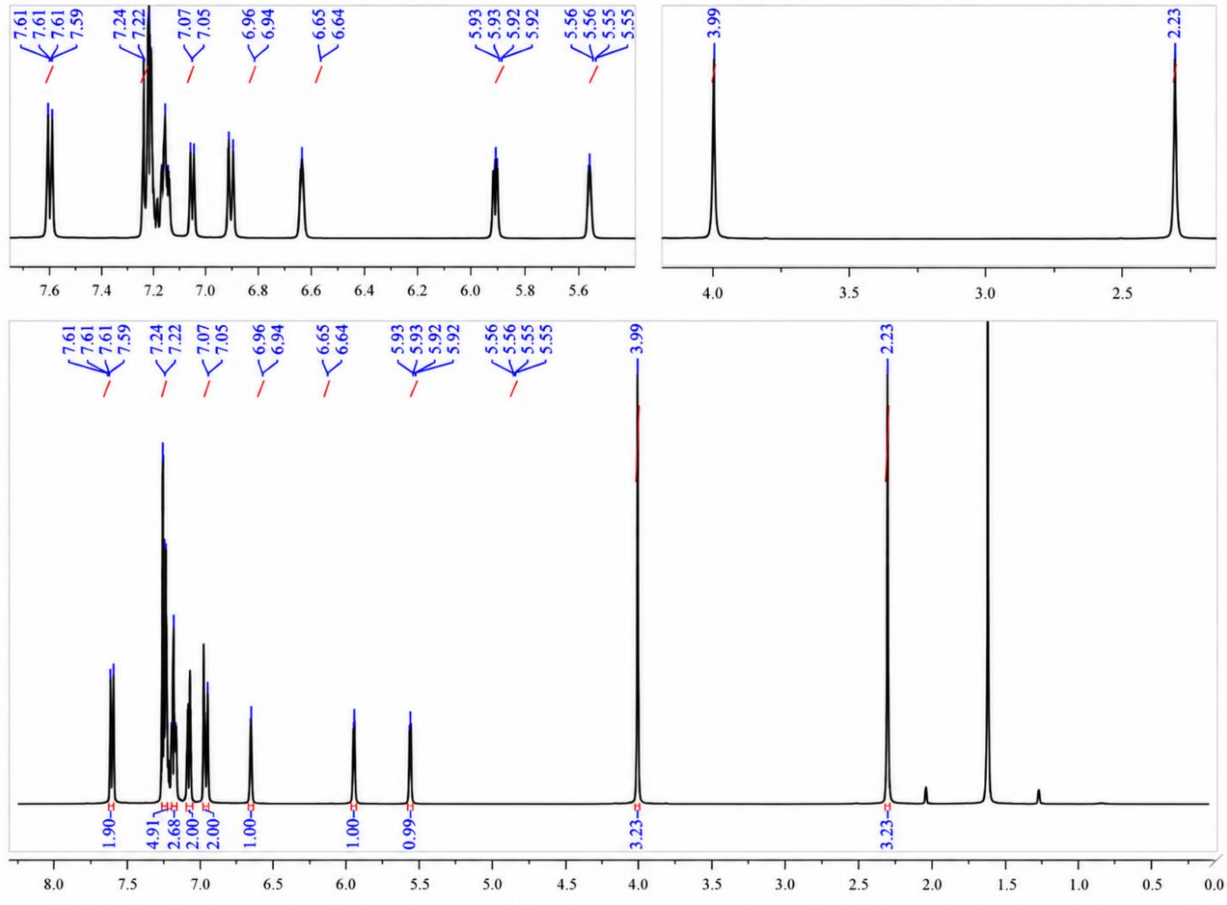

^{13}C NMR

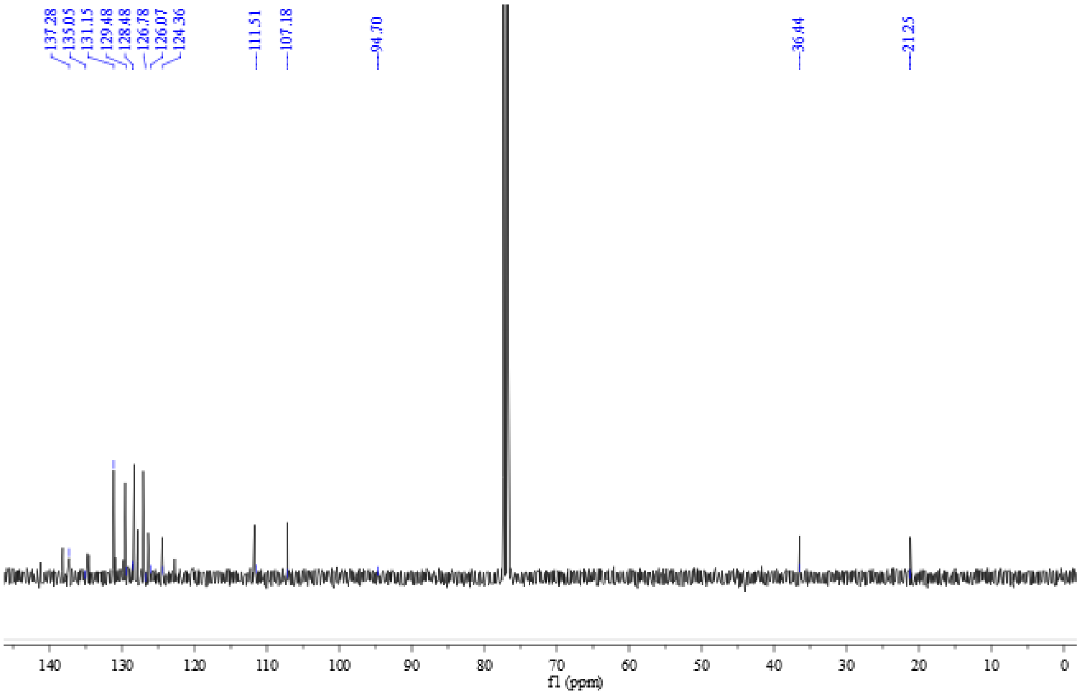
137.28
135.05
131.15
129.48
128.48
126.78
126.07
124.36
111.51
107.18
94.70
36.44
21.25
140
130
120
110
100
90
80
70
60
50
40
30
20
10
0
f1 (ppm)

Mass

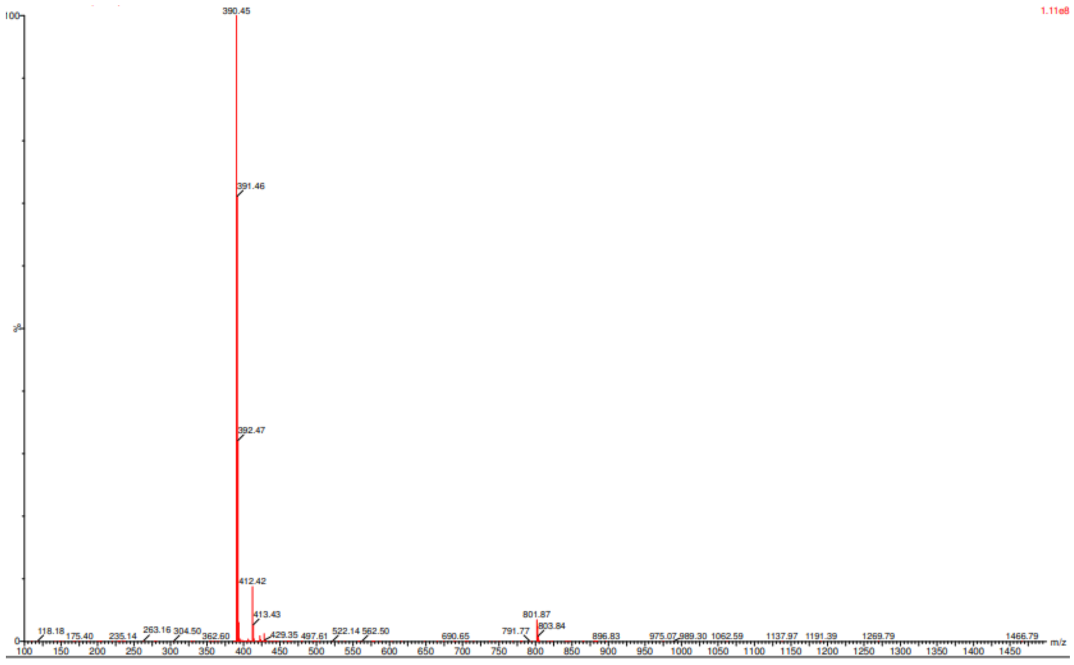
100
%
0
390.45
1.11e8
391.46
392.47
412.42
413.43
801.87
803.84
118.18
175.40
235.14
263.16
304.50
362.60
429.35
497.61
522.14
562.50
690.65
791.77
896.83
975.07
989.30
1062.59
1137.97
1191.39
1269.79
1466.79
m/z

FTIR

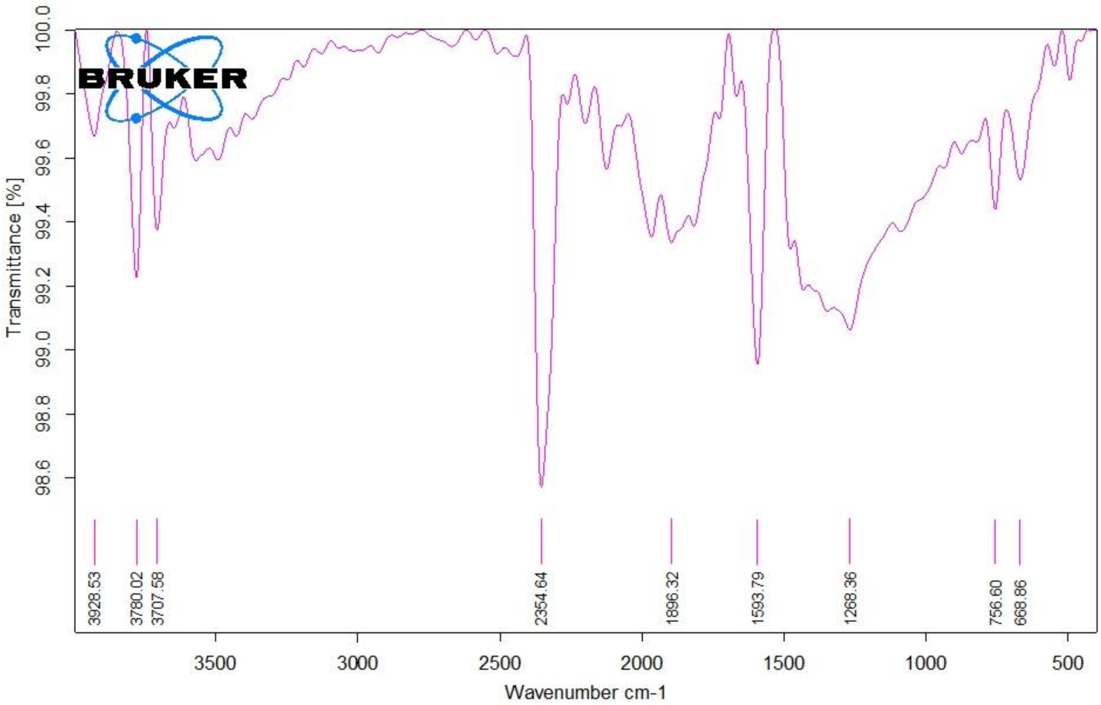
BRUKER
Transmittance [%]
100.0
99.8
99.6
99.4
99.2
99.0
98.8
98.6
3928.53
3780.02
3707.58
2354.64
1896.32
1593.79
1268.36
756.60
668.86
3500
3000
2500
2000
1500
1000
500
Wavenumber cm-1

Characterisation of 5h

1-(4-Chlorophenyl)-2-(1-methyl-1*H*-pyrrol-2-yl)-4,5-diphenyl-1*H*-imidazole

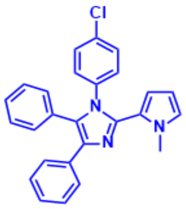

^{1}H NMR

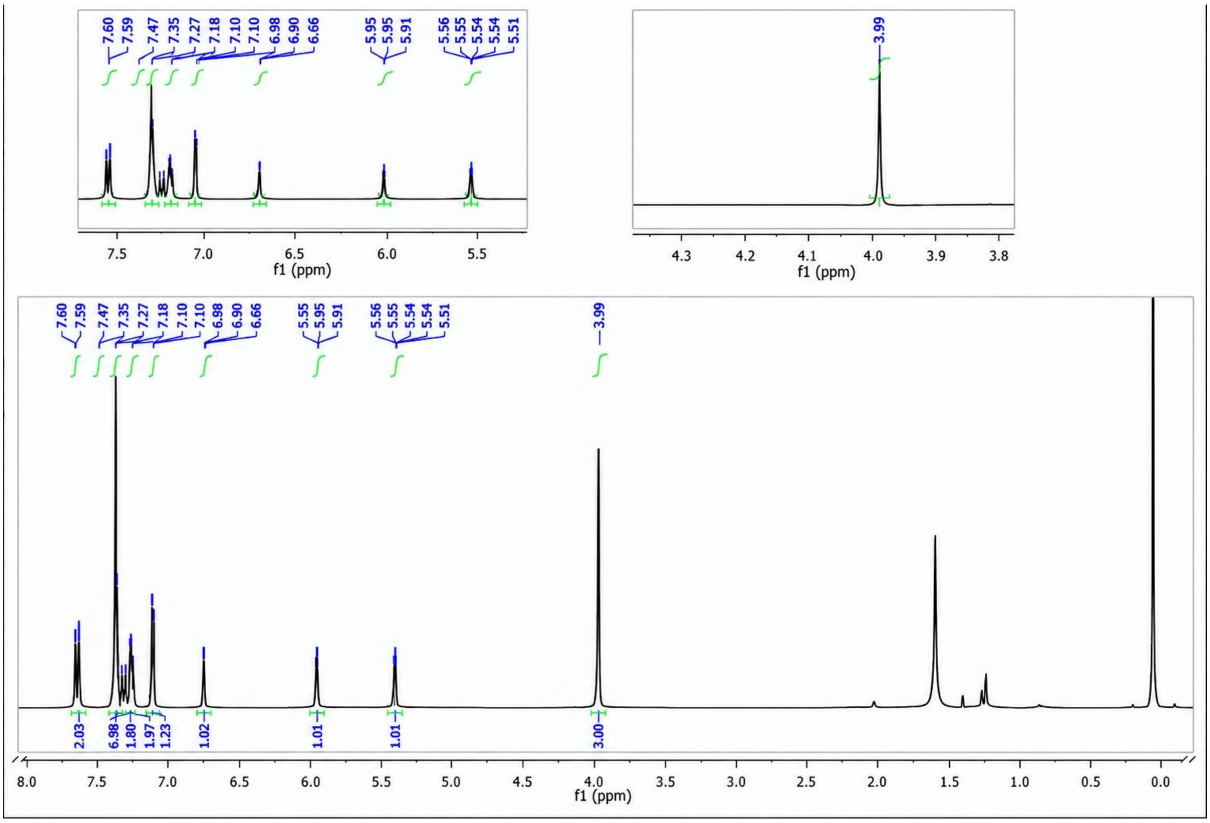

^{13}C NMR

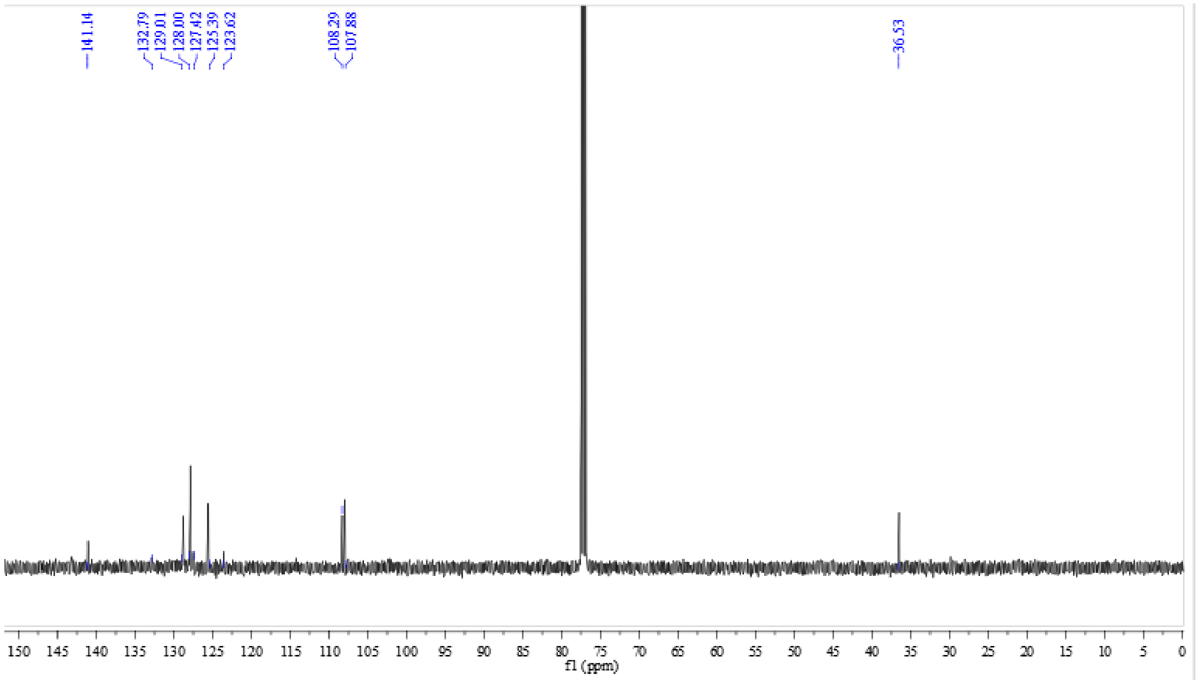

141.14
132.79
129.01
128.00
127.42
125.39
123.62
108.29
107.88
36.53
f1 (ppm)

Mass

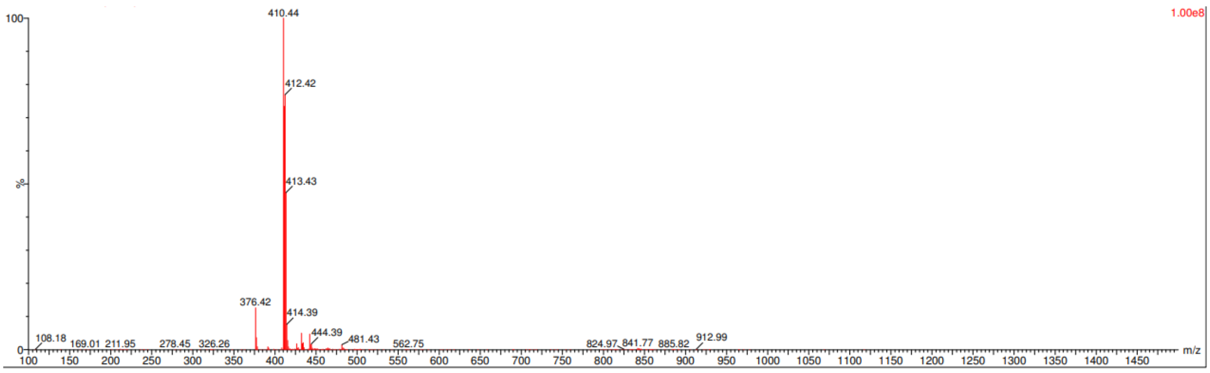

410.44
412.42
413.43
376.42
414.39
444.39
481.43
108.18
169.01
211.95
278.45
326.26
562.75
824.97
841.77
885.82
912.99
1.00e8
m/z
%

FTIR

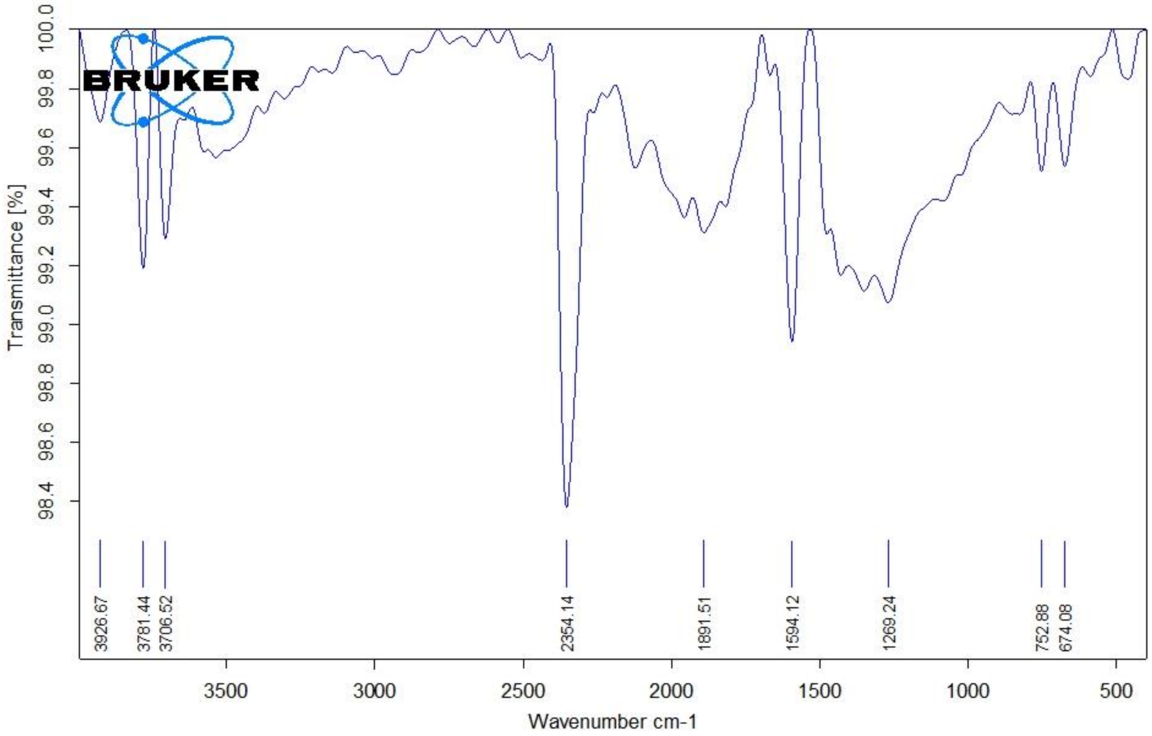
BRUKER
Transmittance [%]
100.0
99.8
99.6
99.4
99.2
99.0
98.8
98.6
98.4
3926.67
3781.44
3706.52
2354.14
1891.51
1594.12
1269.24
752.88
674.08
3500
3000
2500
2000
1500
1000
500
Wavenumber cm-1

Characterisation of 5i

1-(4-Bromophenyl)-2-(1-methyl-1*H*-pyrrol-2-yl)-4,5-diphenyl-1*H*-imidazole

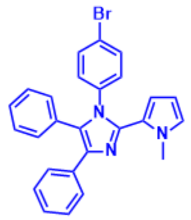

^{1}H NMR

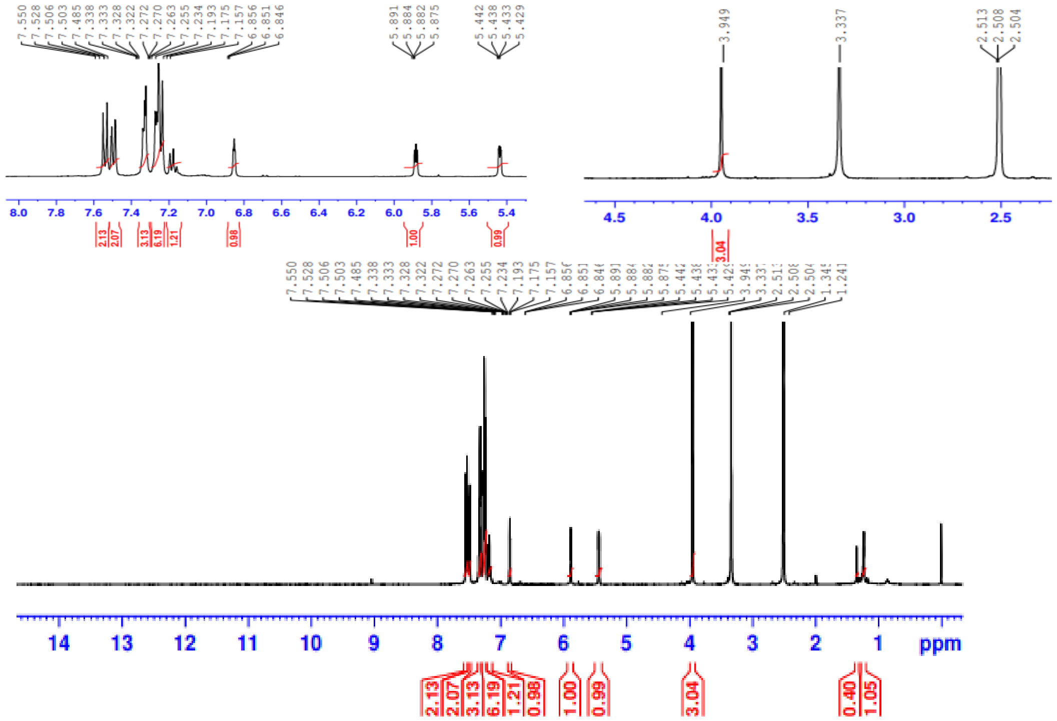

^{13}C NMR

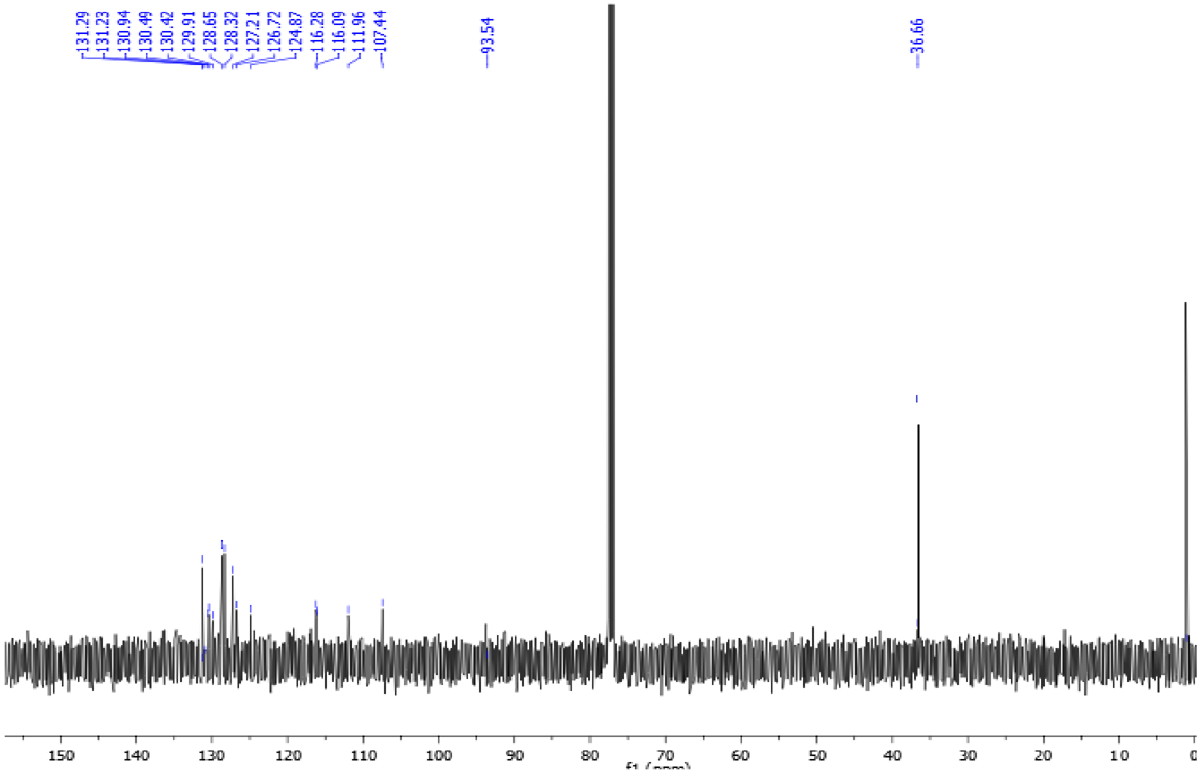
131.29
131.23
130.94
130.49
130.42
129.91
128.65
128.32
127.21
126.72
124.87
116.28
116.09
111.96
107.44
93.54
36.66
150
140
130
120
110
100
90
80
70
60
50
40
30
20
10
0

Mass

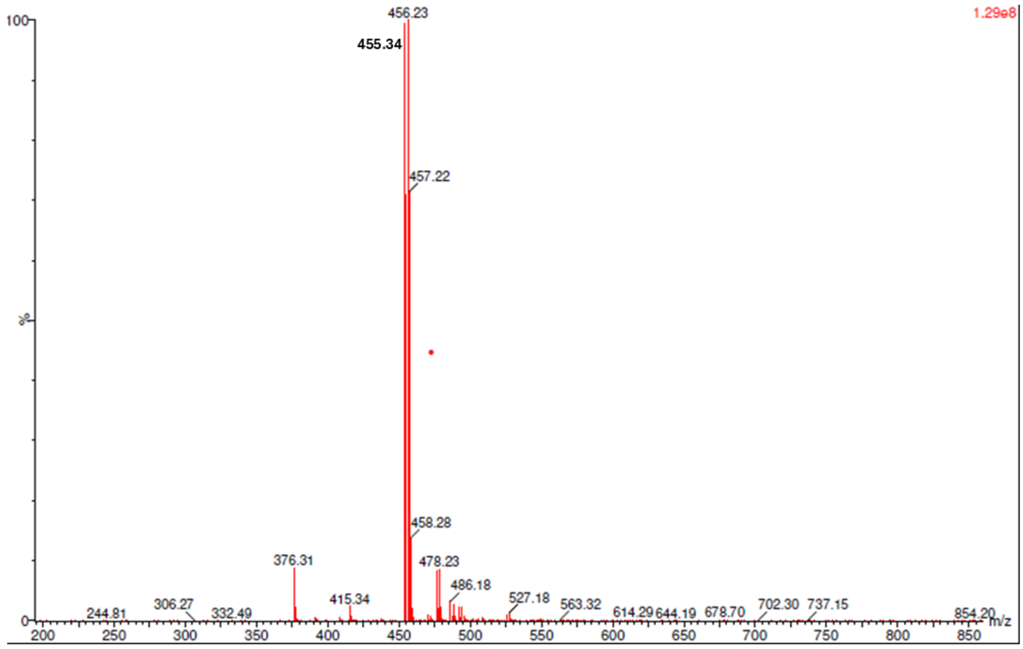
100
%
0
456.23
455.34
457.22
458.28
478.23
486.18
376.31
415.34
527.18
563.32
244.81
306.27
332.49
614.29
644.19
678.70
702.30
737.15
854.20
m/z
1.29e8
200
250
300
350
400
450
500
550
600
650
700
750
800
850

FTIR

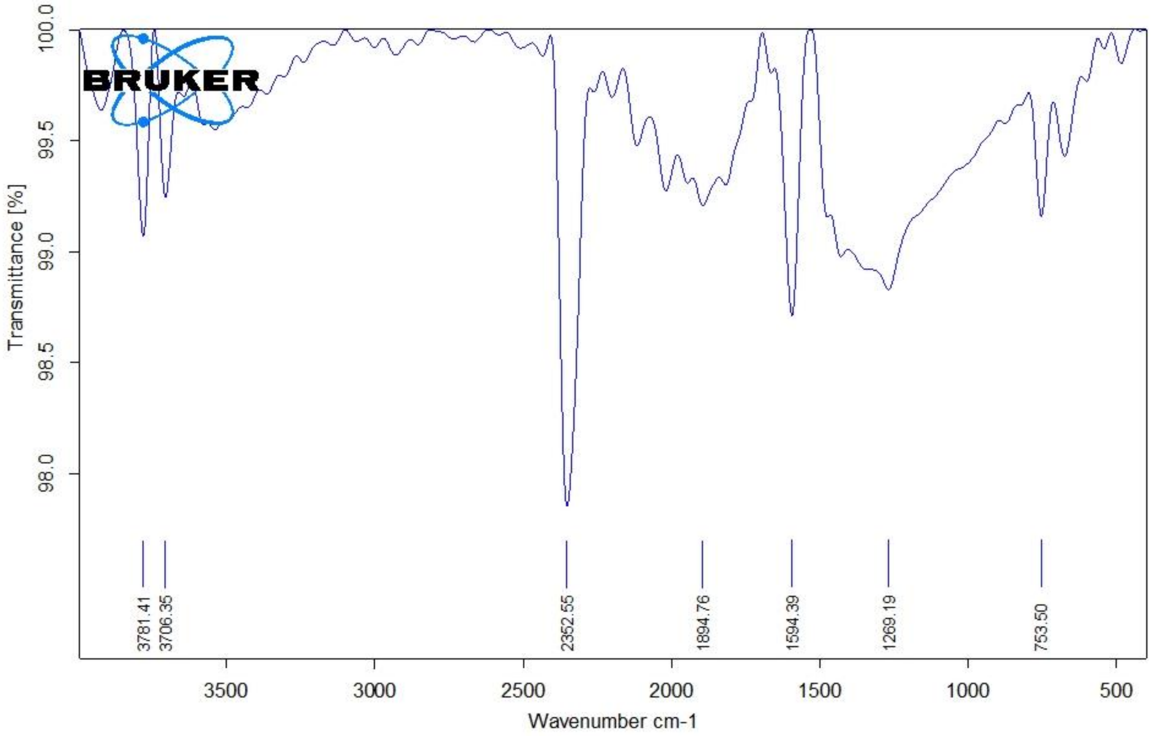

100.0
99.5
99.0
98.5
98.0
Transmittance [%]
BRUKER
3781.41
3706.35
2352.55
1894.76
1594.39
1269.19
753.50
3500
3000
2500
2000
1500
1000
500
Wavenumber cm-1

Characterisation of 5j

4-(2-(2-(1-Methyl-1*H*-pyrrol-2-yl)-4,5-diphenyl-1*H*-imidazol-1-yl) ethyl) morpholine

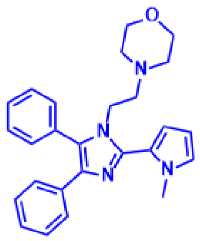

^{1}H NMR

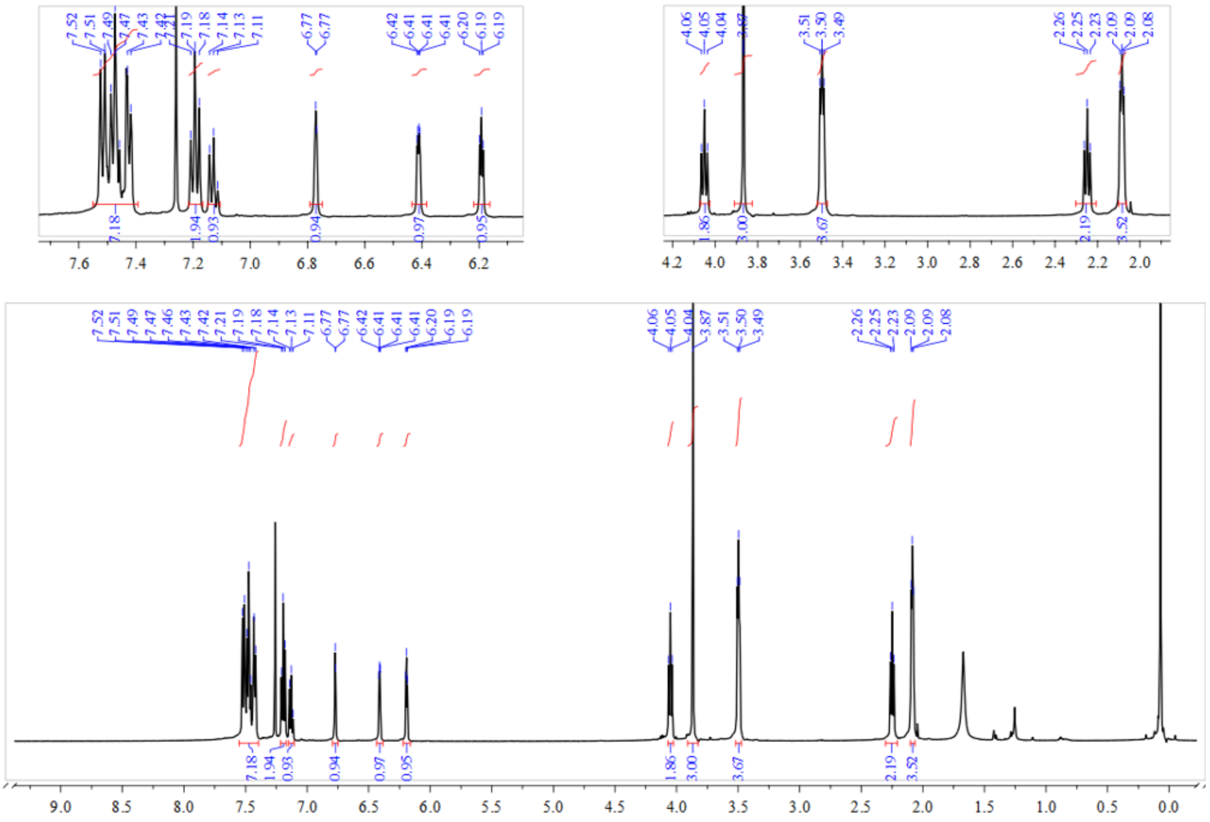

^{13}C NMR

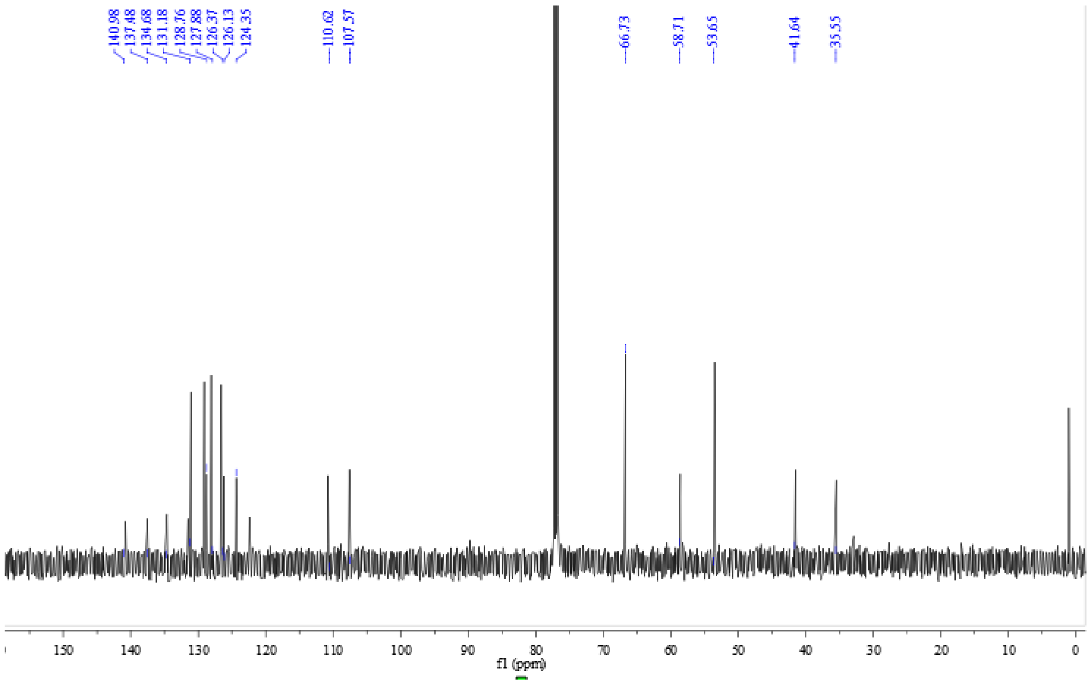

140.98
137.48
134.68
131.18
128.76
127.88
126.37
126.13
124.35
110.62
107.57
66.73
58.71
53.65
41.64
35.55
f1 (ppm)

Mass

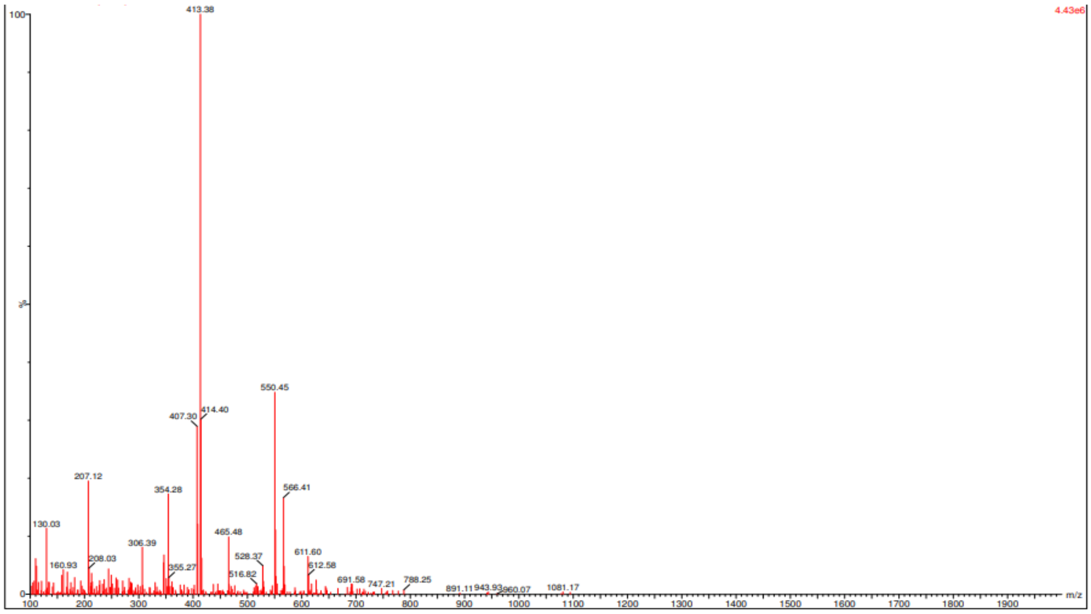

413.38
4.43e6
550.45
407.30
414.40
207.12
354.28
566.41
130.03
465.48
306.39
611.60
528.37
160.93
208.03
355.27
612.58
516.82
691.58
747.21
788.25
891.11
943.93
960.07
1081.17
m/z

FTIR

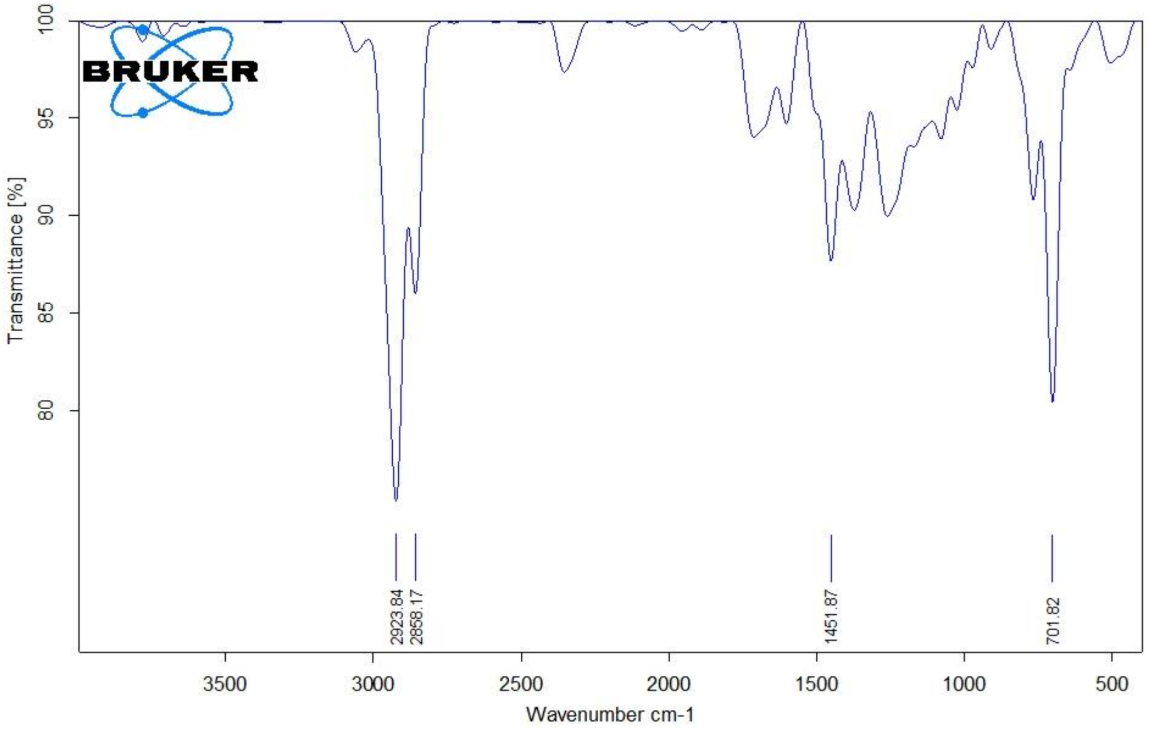

BRUKER
Transmittance [%]
100
95
90
85
80
2923.84
2858.17
1451.87
701.82
3500
3000
2500
2000
1500
1000
500
Wavenumber cm-1

Characterisation of 5k

1-(Heptan-2-yl)-2-(1-methyl-1H-pyrrol-2-yl)-4,5-diphenyl-1H-imidazole

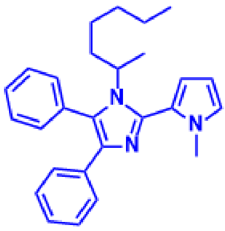

^{1}H NMR

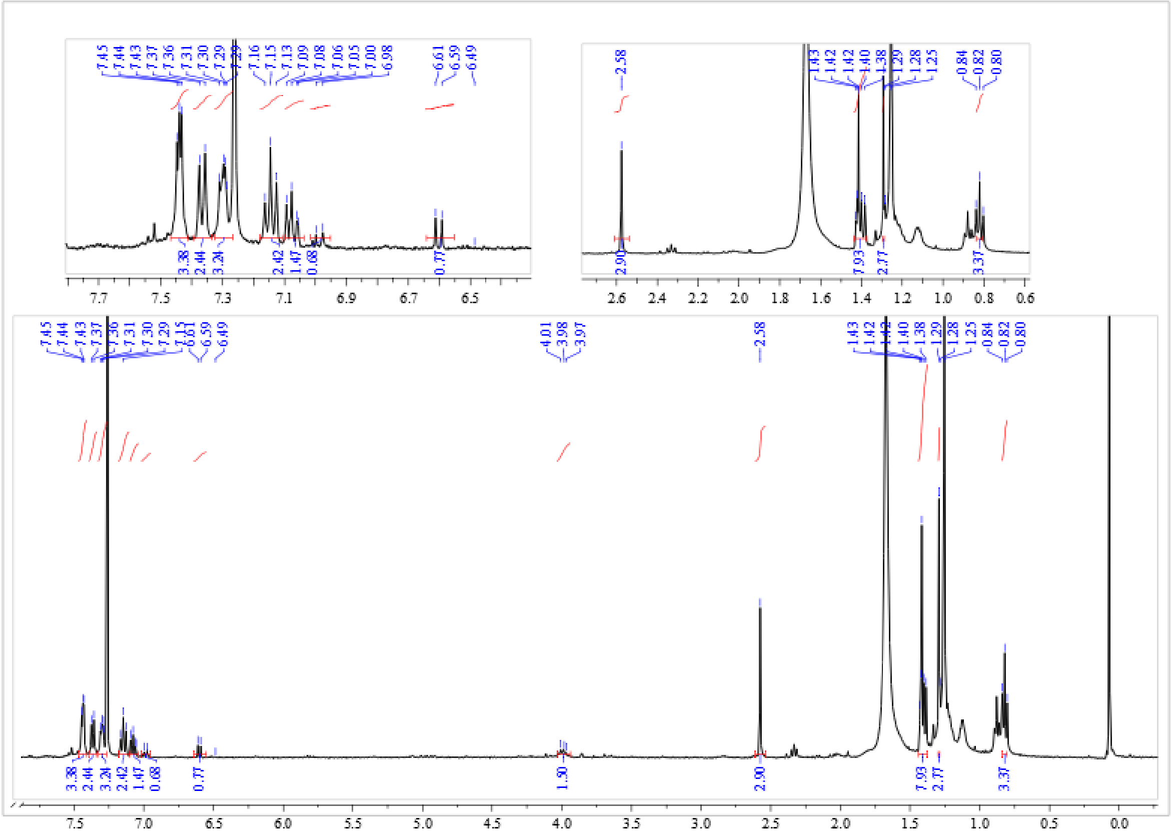

^{13}C NMR

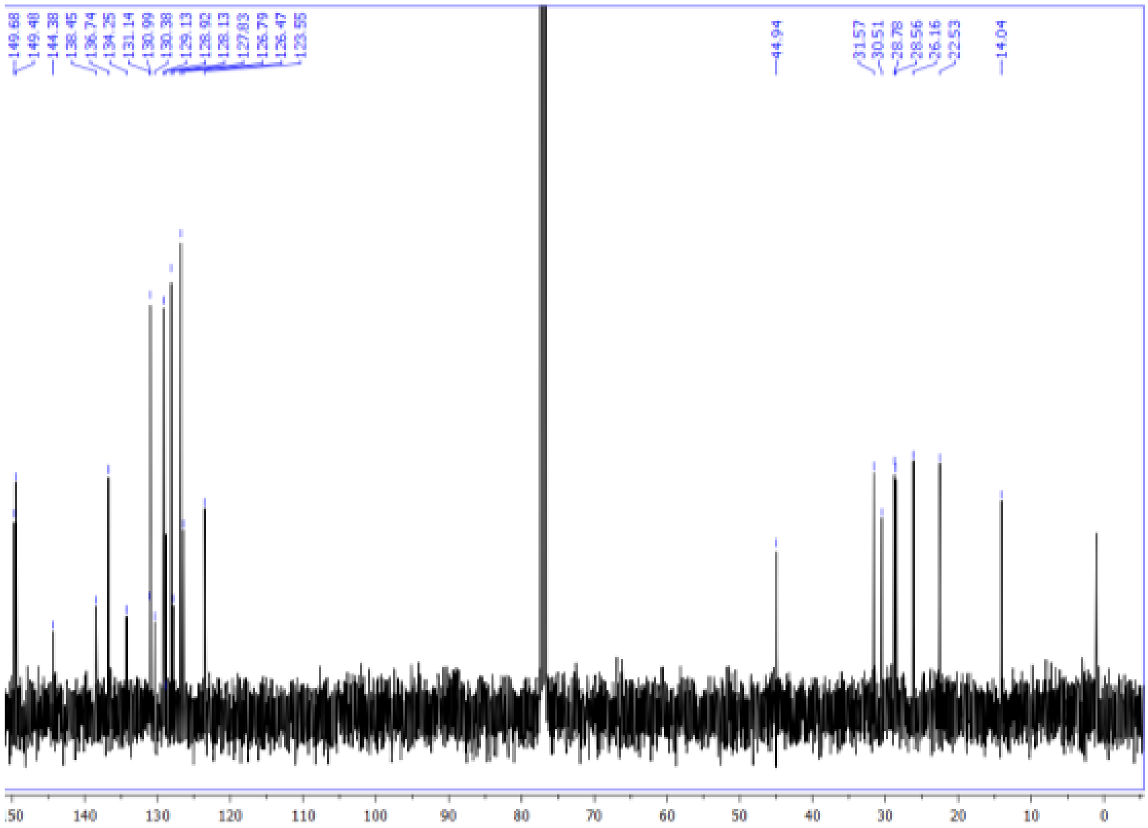

149.68
149.48
144.38
138.45
136.74
134.25
131.14
130.99
130.38
129.13
128.92
128.13
127.83
126.79
126.47
123.55
44.94
31.57
30.51
28.78
28.56
26.16
22.53
14.04

Mass

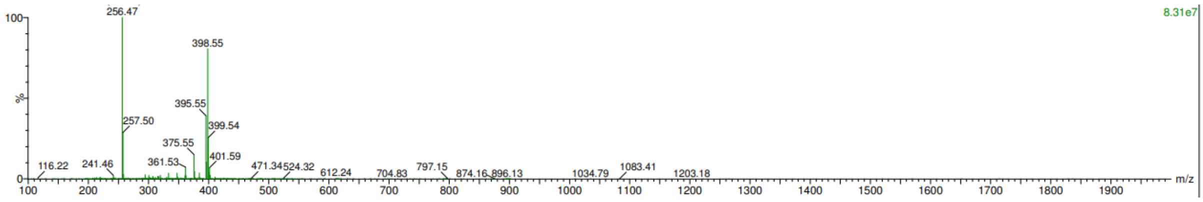

256.47
398.55
395.55
257.50
399.54
375.55
401.59
361.53
116.22
241.46
471.34
524.32
612.24
704.83
797.15
874.16
896.13
1034.79
1083.41
1203.18
8.31e7
m/z
%

FTIR

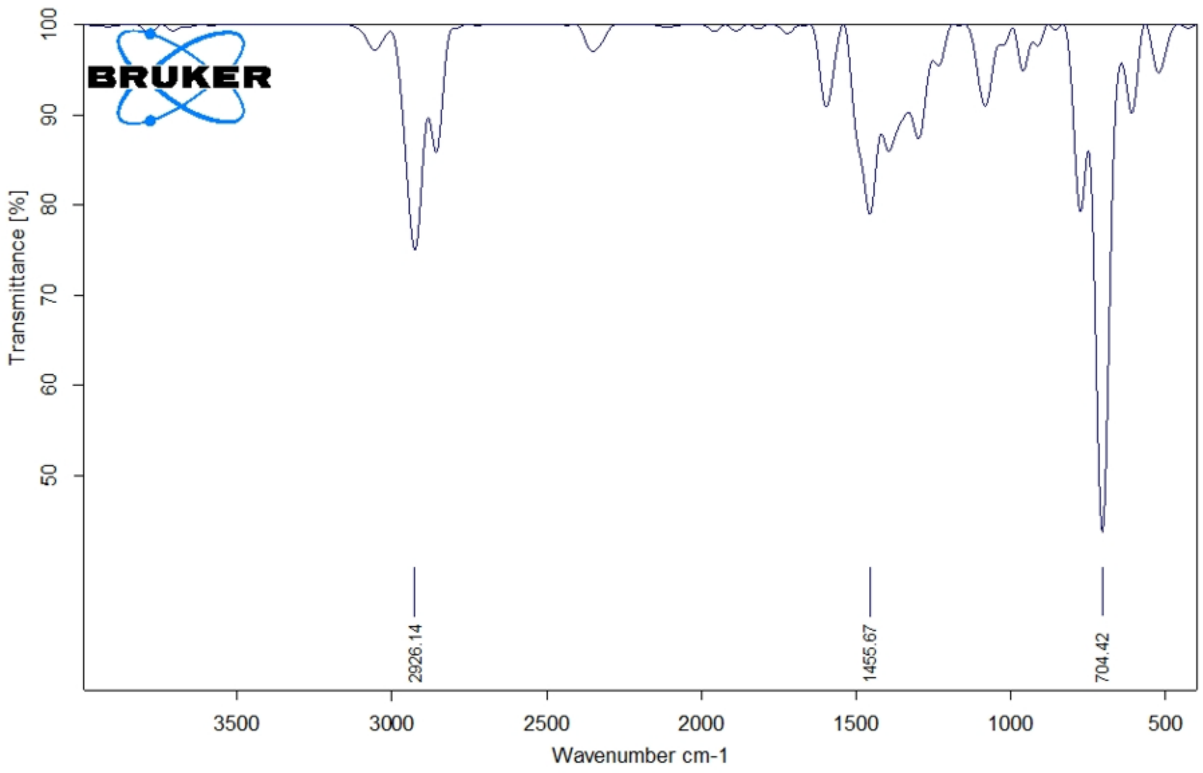

BRUKER
Transmittance [%]
100
90
80
70
60
50
2926.14
1455.67
704.42
3500
3000
2500
2000
1500
1000
500
Wavenumber cm-1

Characterisation of 6a

2-(1-Methyl-1*H*-pyrrol-2-yl)-1*H*-phenanthro[9,10-*d*] imidazole

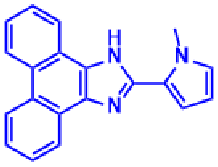

^{1}H NMR

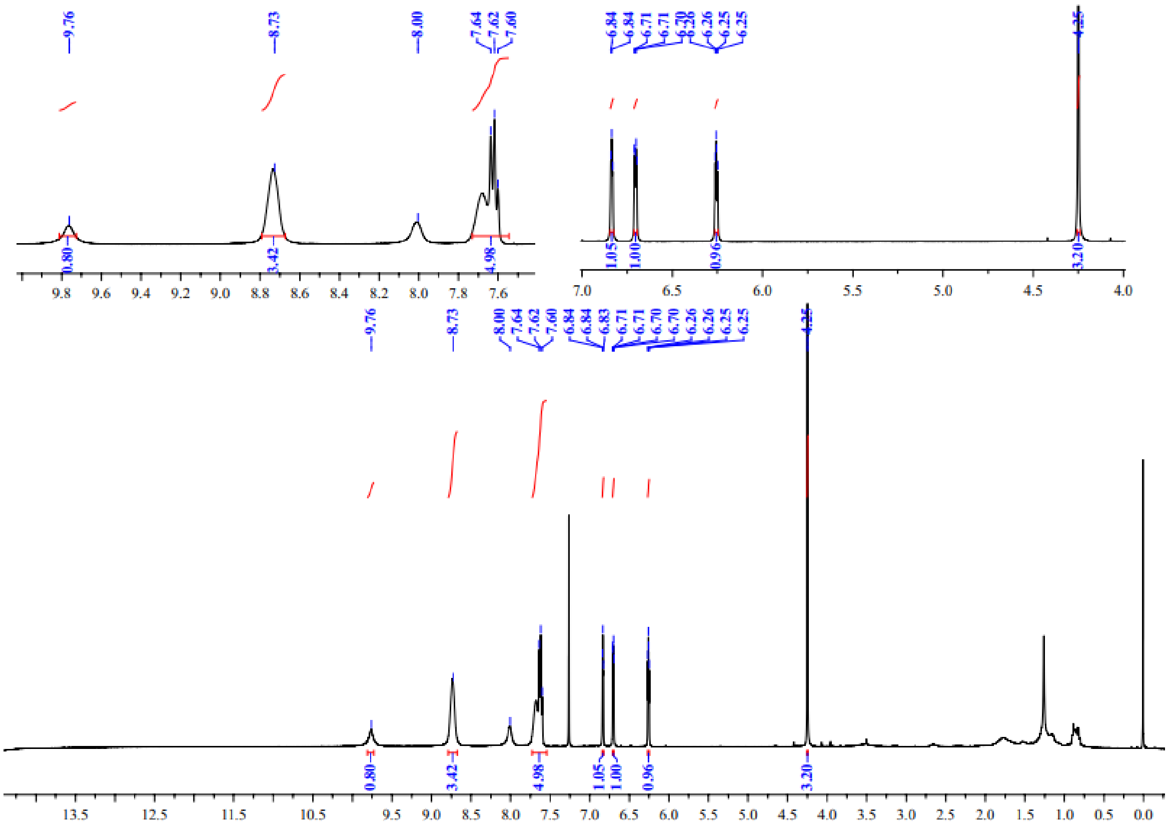

^{13}C NMR

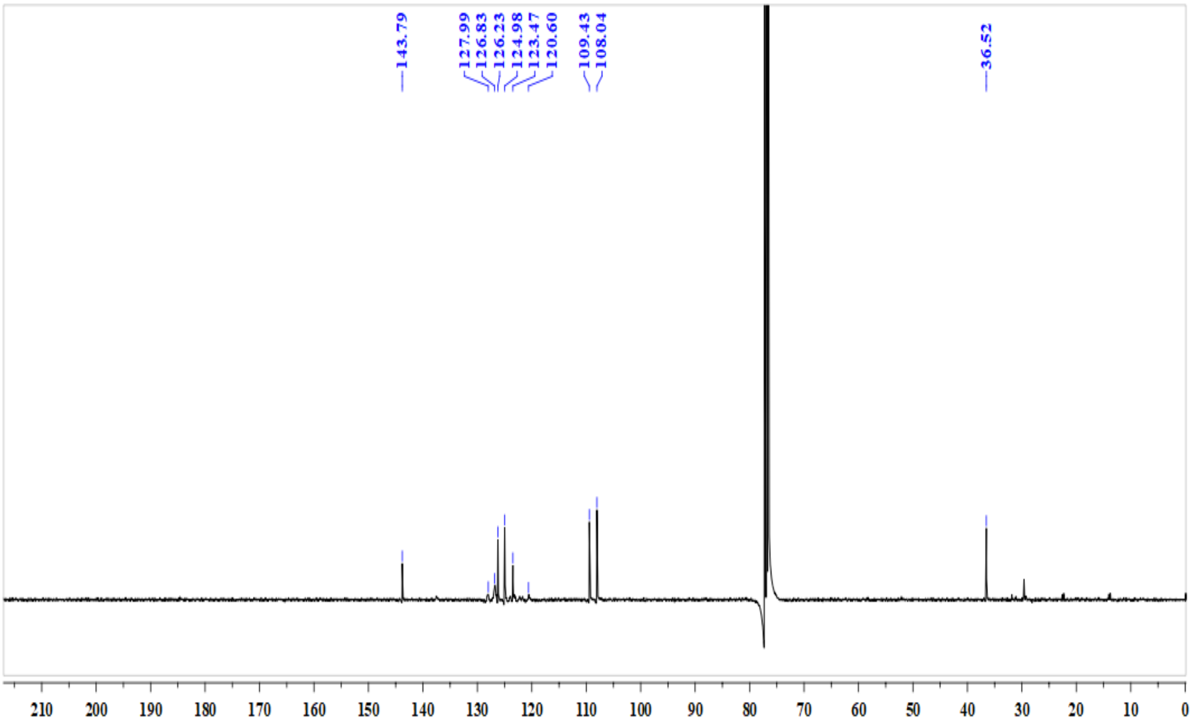

143.79
127.99
126.83
126.23
124.98
123.47
120.60
109.43
108.04
36.52
210
200
190
180
170
160
150
140
130
120
110
100
90
80
70
60
50
40
30
20
10
0

HRMS

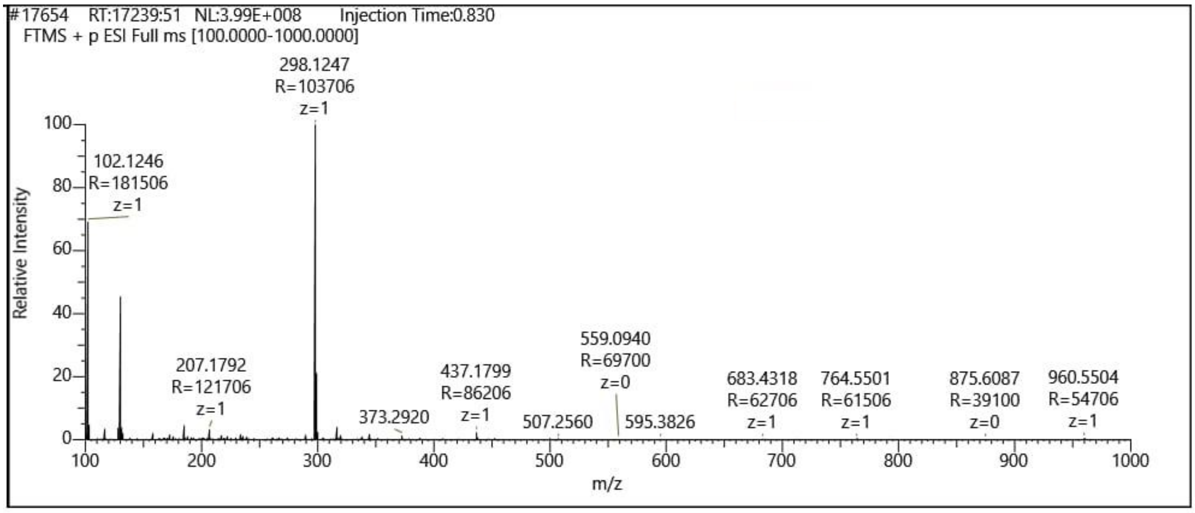

#17654 RT:17239:51 NL:3.99E+008 Injection Time:0.830
FTMS + p ESI Full ms [100.0000-1000.0000]
298.1247
R=103706
z=1
102.1246
R=181506
z=1
207.1792
R=121706
z=1
373.2920
437.1799
R=86206
z=1
507.2560
559.0940
R=69700
z=0
595.3826
683.4318
R=62706
z=1
764.5501
R=61506
z=1
875.6087
R=39100
z=0
960.5504
R=54706
z=1
Relative Intensity
m/z

FTIR

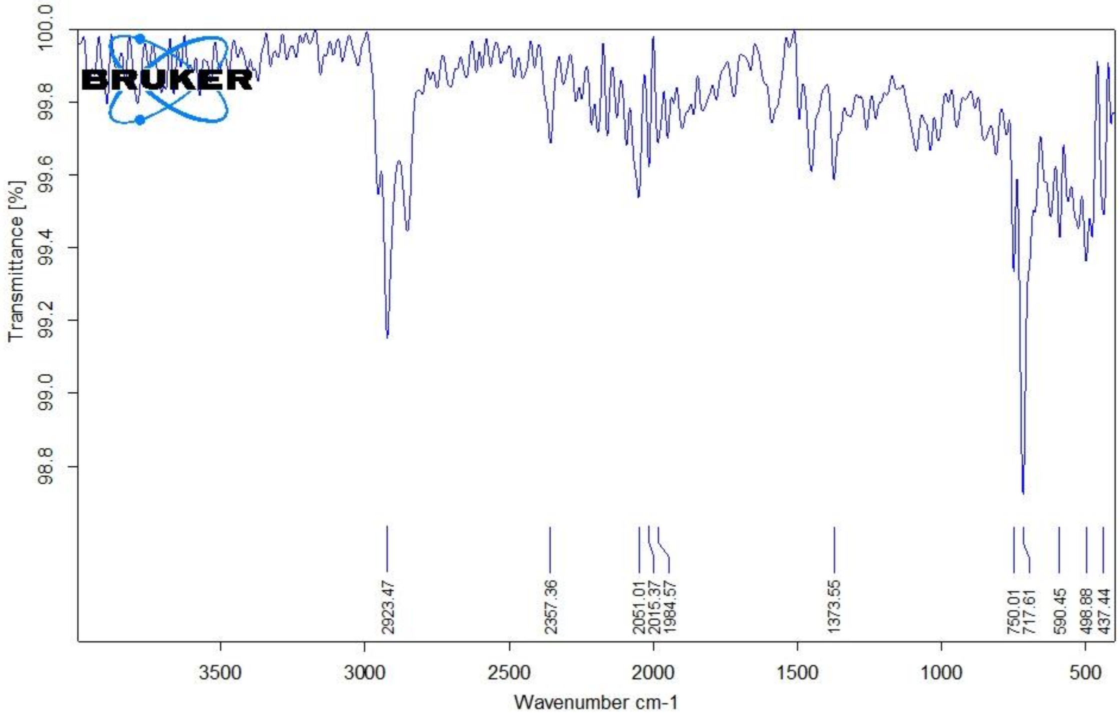

BRUKER
Transmittance [%]
100.0
99.8
99.6
99.4
99.2
99.0
98.8
2923.47
2357.36
2051.01
2015.37
1984.57
1373.55
750.01
717.61
590.45
498.88
437.44
3500
3000
2500
2000
1500
1000
500
Wavenumber cm-1

Characterisation of 6b

1-Ethyl-2-(1-methyl-1*H*-pyrrol-2-yl)-*1H*-phenanthro[9,10-*d*] imidazole

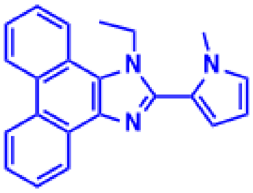

^{1}H NMR

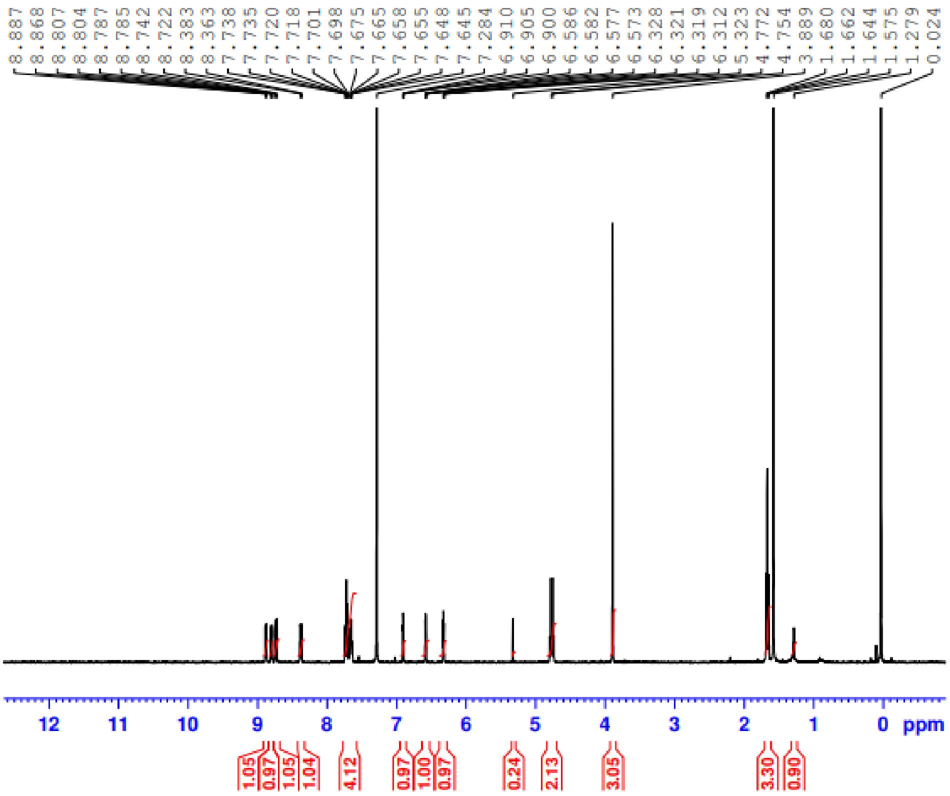

^{13}C NMR

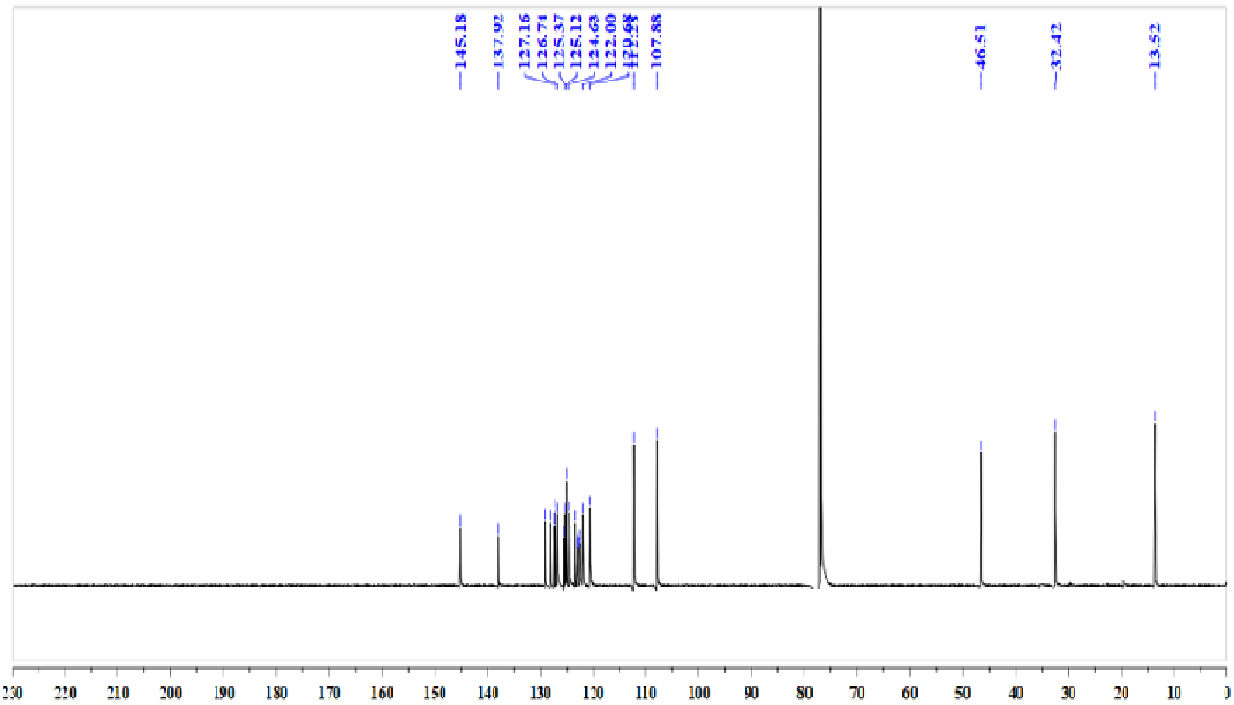
145.18
137.92
127.16
126.71
125.37
125.12
124.63
122.00
107.88
46.51
32.42
13.52
230
220
210
200
190
180
170
160
150
140
130
120
110
100
90
80
70
60
50
40
30
20
10
0

HRMS

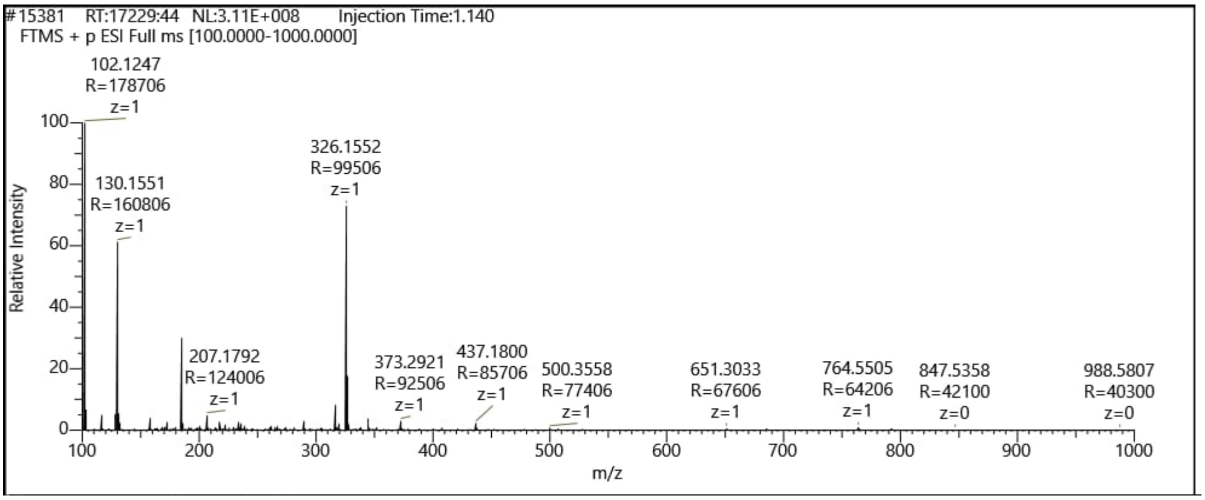
#15381 RT:17229:44 NL:3.11E+008 Injection Time:1.140
FTMS + p ESI Full ms [100.0000-1000.0000]
Relative Intensity
m/z
102.1247 R=178706 z=1
130.1551 R=160806 z=1
207.1792 R=124006 z=1
326.1552 R=99506 z=1
373.2921 R=92506 z=1
437.1800 R=85706 z=1
500.3558 R=77406 z=1
651.3033 R=67606 z=1
764.5505 R=64206 z=1
847.5358 R=42100 z=0
988.5807 R=40300 z=0

FTIR

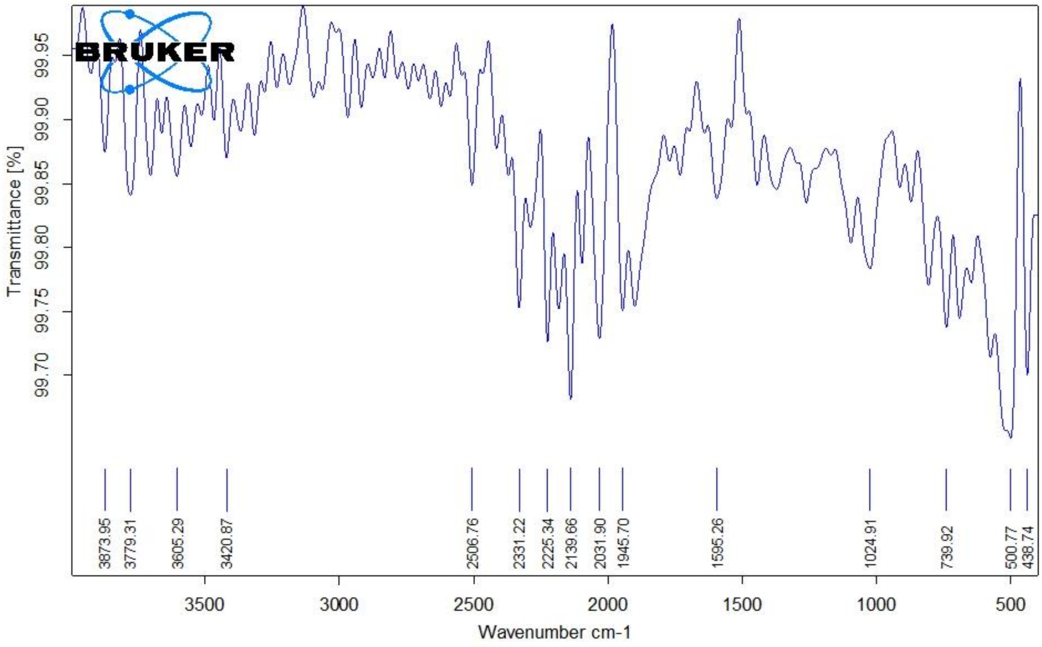

BRUKER
Transmittance [%]
99.70
99.75
99.80
99.85
99.90
99.95
3873.95
3779.31
3605.29
3420.87
2506.76
2331.22
2225.34
2139.66
2031.90
1945.70
1595.26
1024.91
739.92
500.77
438.74
3500
3000
2500
2000
1500
1000
500
Wavenumber cm-1

Characterisation of 6c

1-Butyl-2-(1-methyl-1H-pyrrol-2-yl)-1H-phenanthro[9,10-d] imidazole

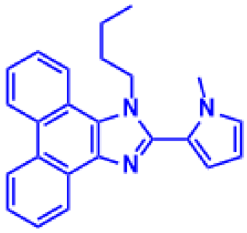

^{1}H NMR

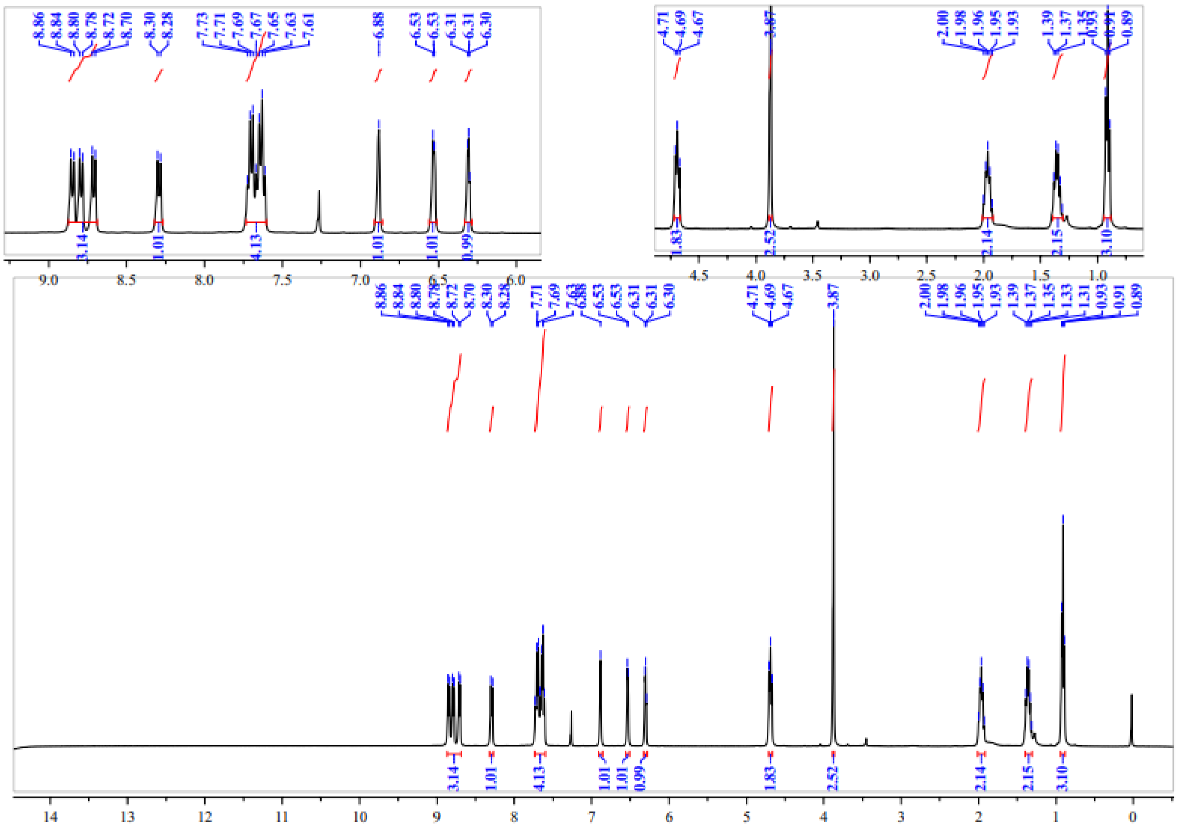

^{13}C NMR

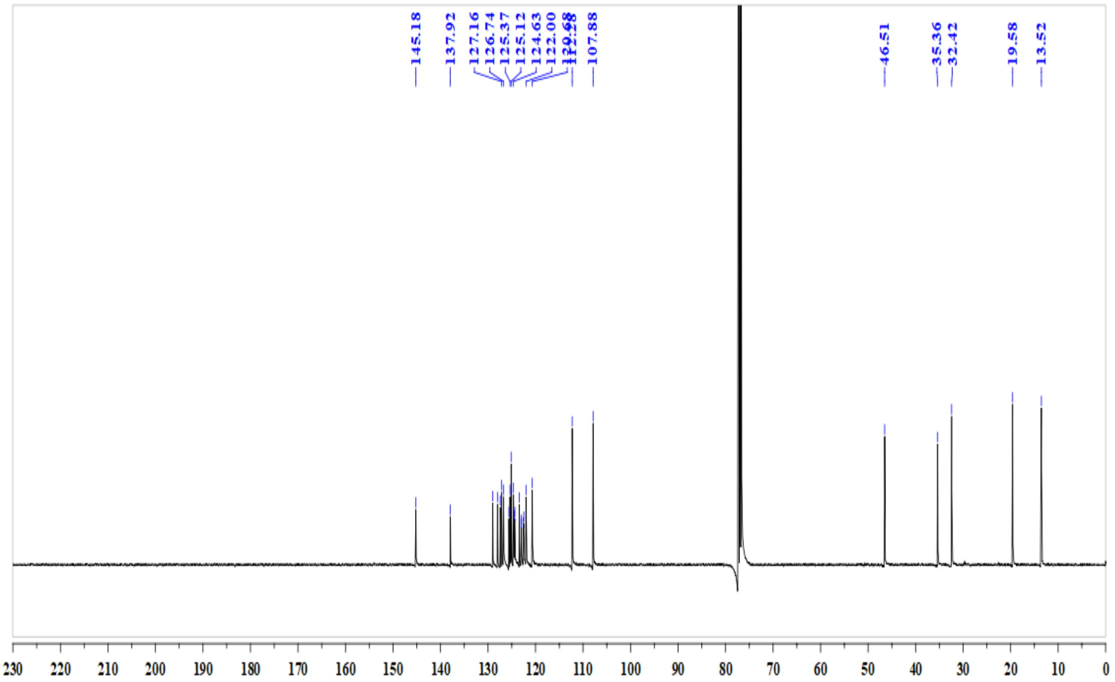
145.18
137.92
127.16
126.74
125.37
125.12
124.63
122.00
107.88
46.51
35.36
32.42
19.58
13.52
230 220 210 200 190 180 170 160 150 140 130 120 110 100 90 80 70 60 50 40 30 20 10 0

HRMS

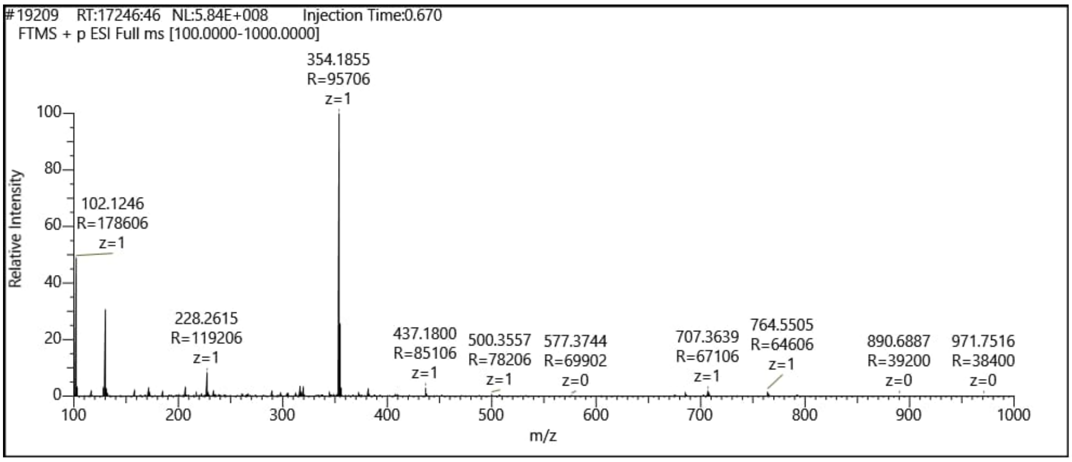
#19209 RT:17246:46 NL:5.84E+008 Injection Time:0.670
FTMS + p ESI Full ms [100.0000-1000.0000]
354.1855
R=95706
z=1
102.1246
R=178606
z=1
228.2615
R=119206
z=1
437.1800
R=85106
z=1
500.3557
R=78206
z=1
577.3744
R=69902
z=0
707.3639
R=67106
z=1
764.5505
R=64606
z=1
890.6887
R=39200
z=0
971.7516
R=38400
z=0
Relative Intensity
m/z

FTIR

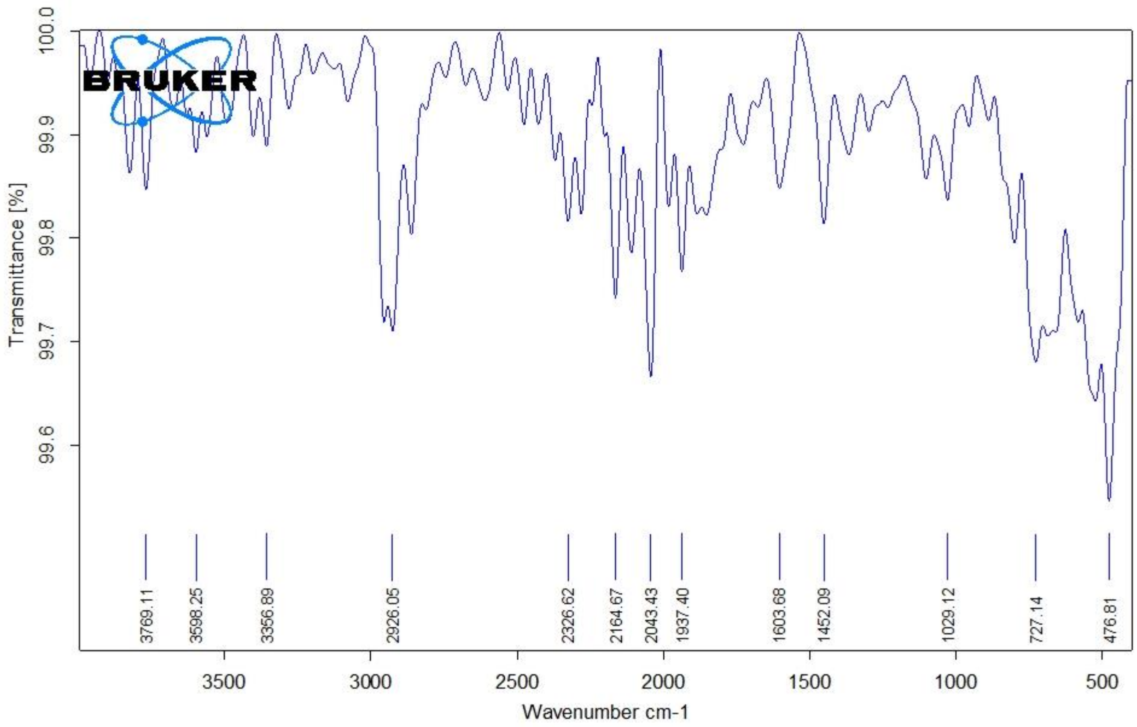

BRUKER
Transmittance [%]
100.0
99.9
99.8
99.7
99.6
3769.11
3598.25
3356.89
2926.05
2326.62
2164.67
2043.43
1937.40
1603.68
1452.09
1029.12
727.14
476.81
3500
3000
2500
2000
1500
1000
500
Wavenumber cm-1

Characterisation of 6d

1-Hexyl-2-(1-methyl-*1H*-pyrrol-2-yl)-1*H*-phenanthro[9,10-*d*]imidazole

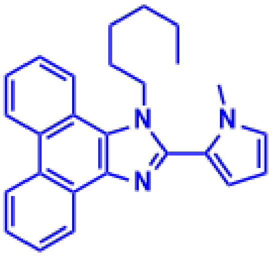

^{1}H NMR

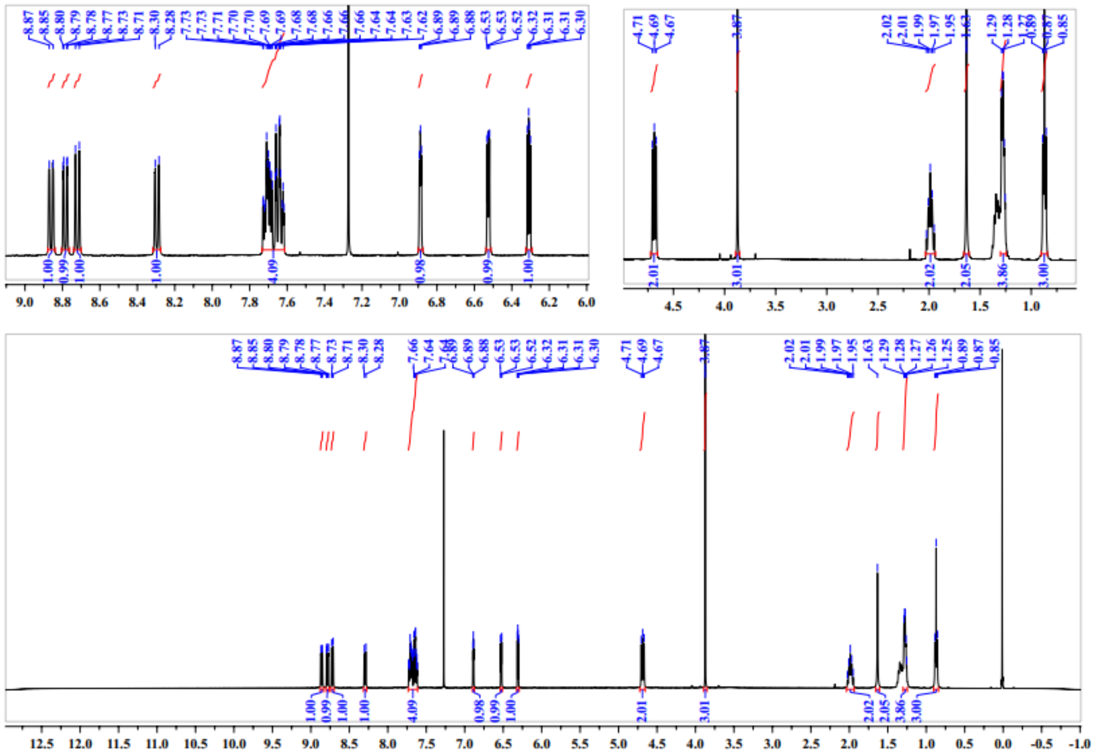

^{13}C NMR

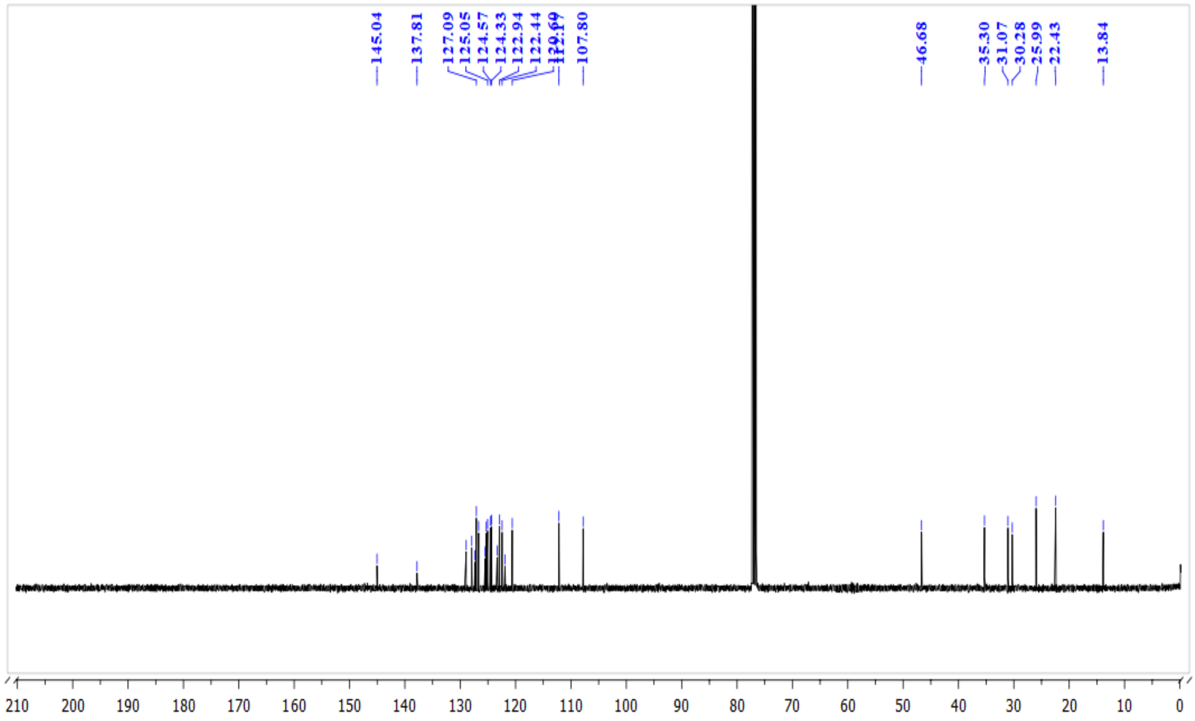

145.04
137.81
127.09
125.05
124.57
124.33
122.94
122.44
120.69
112.17
107.80
46.68
35.30
31.07
30.28
25.99
22.43
13.84
210 200 190 180 170 160 150 140 130 120 110 100 90 80 70 60 50 40 30 20 10 0

HRMS

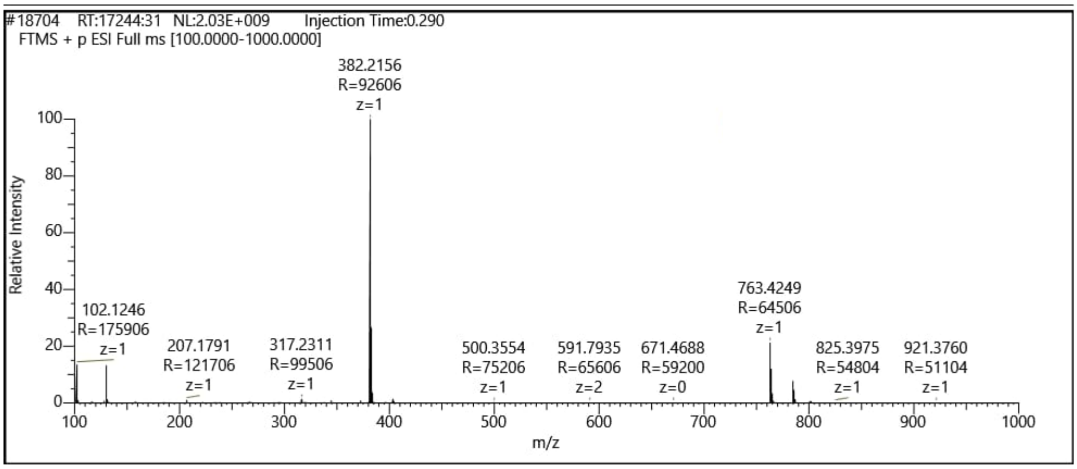

#18704 RT:17244:31 NL:2.03E+009 Injection Time:0.290
FTMS + p ESI Full ms [100.0000-1000.0000]
382.2156
R=92606
z=1
Relative Intensity
102.1246
R=175906
z=1
207.1791
R=121706
z=1
317.2311
R=99506
z=1
500.3554
R=75206
z=1
591.7935
R=65606
z=2
671.4688
R=59200
z=0
763.4249
R=64506
z=1
825.3975
R=54804
z=1
921.3760
R=51104
z=1
m/z

FTIR

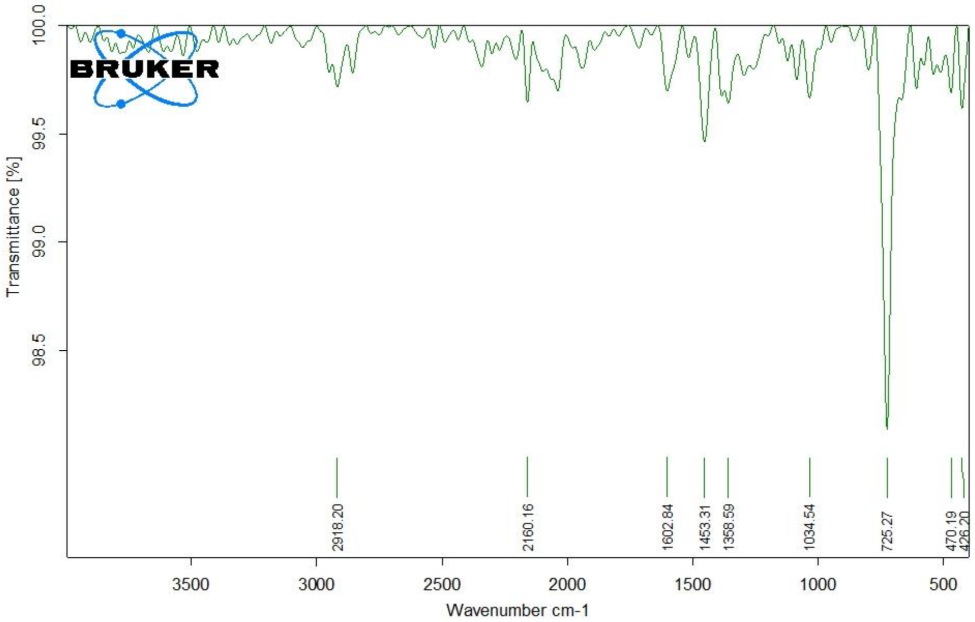

100.0
99.5
99.0
98.5
Transmittance [%]
BRUKER
2918.20
2160.16
1602.84
1453.31
1358.59
1034.54
725.27
470.19
426.20
3500
3000
2500
2000
1500
1000
500
Wavenumber cm-1

Characterisation of 6e

2-(1-Methyl-1*H*-pyrrol-2-yl)-1-octyl-*1H*-phenanthro[9,10-*d*]imidazole

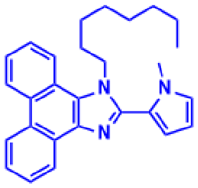

^{1}H NMR

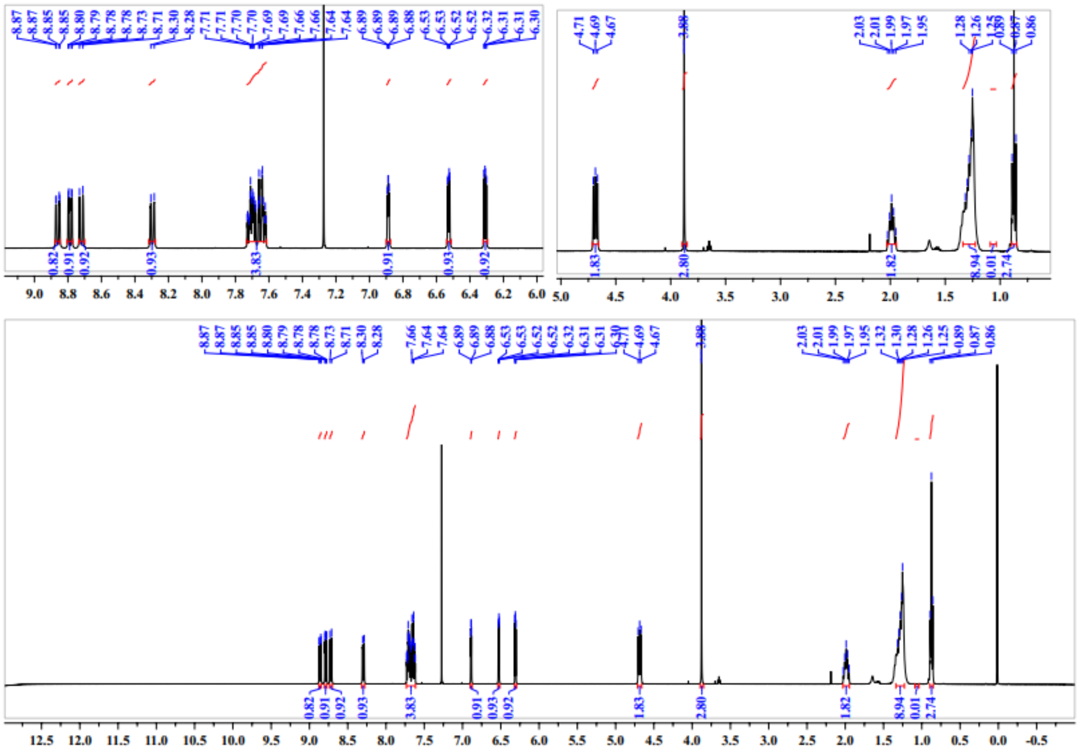

^{13}C NMR

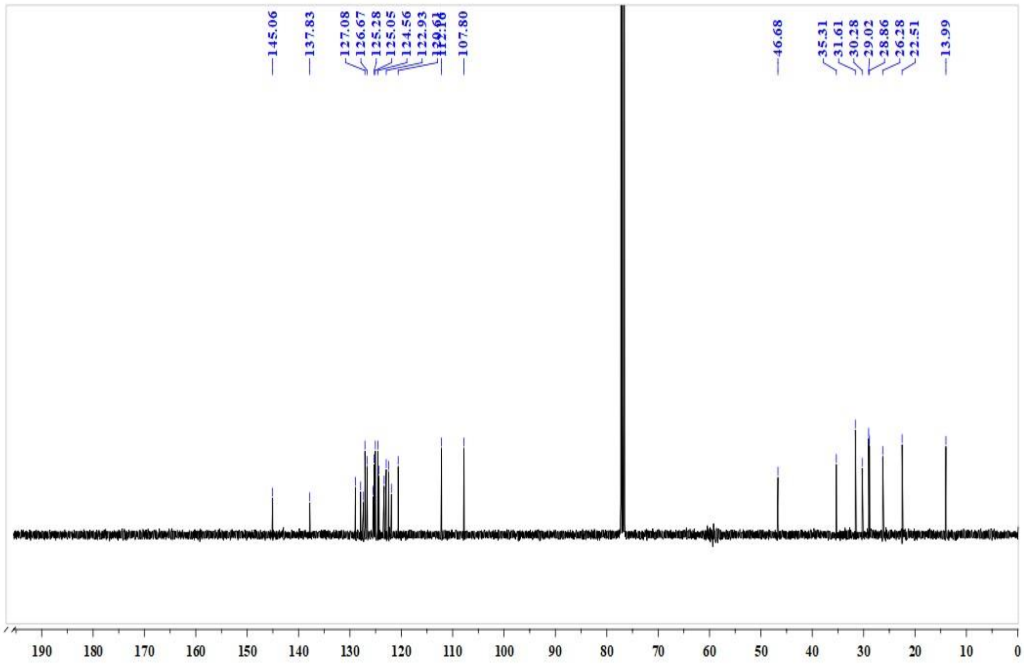
145.06
137.83
127.08
126.67
125.28
125.05
124.56
122.93
107.80
46.68
35.31
31.61
30.28
29.02
28.86
26.28
22.51
13.99
190 180 170 160 150 140 130 120 110 100 90 80 70 60 50 40 30 20 10 0

HRMS

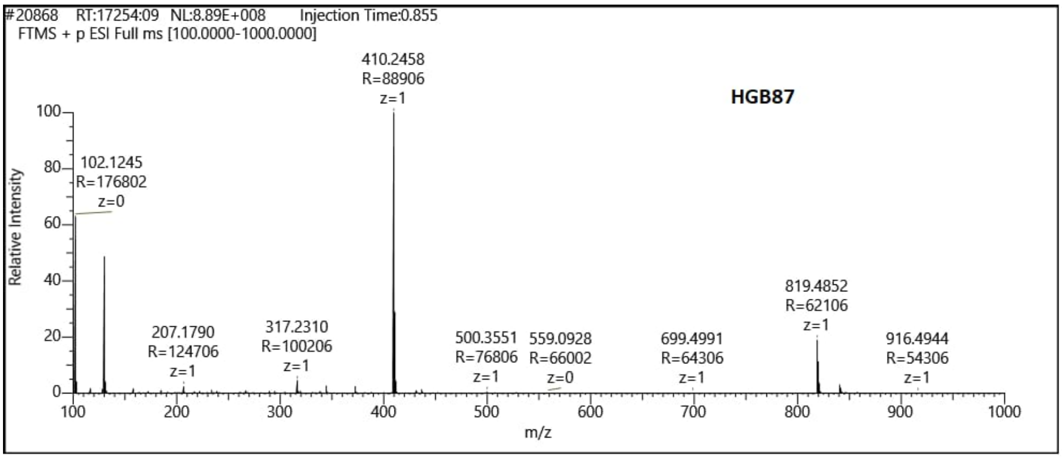
#20868 RT:17254:09 NL:8.89E+008 Injection Time:0.855
FTMS + p ESI Full ms [100.0000-1000.0000]
HGB87
410.2458
R=88906
z=1
102.1245
R=176802
z=0
207.1790
R=124706
z=1
317.2310
R=100206
z=1
500.3551
R=76806
z=1
559.0928
R=66002
z=0
699.4991
R=64306
z=1
819.4852
R=62106
z=1
916.4944
R=54306
z=1
Relative Intensity
m/z

FTIR

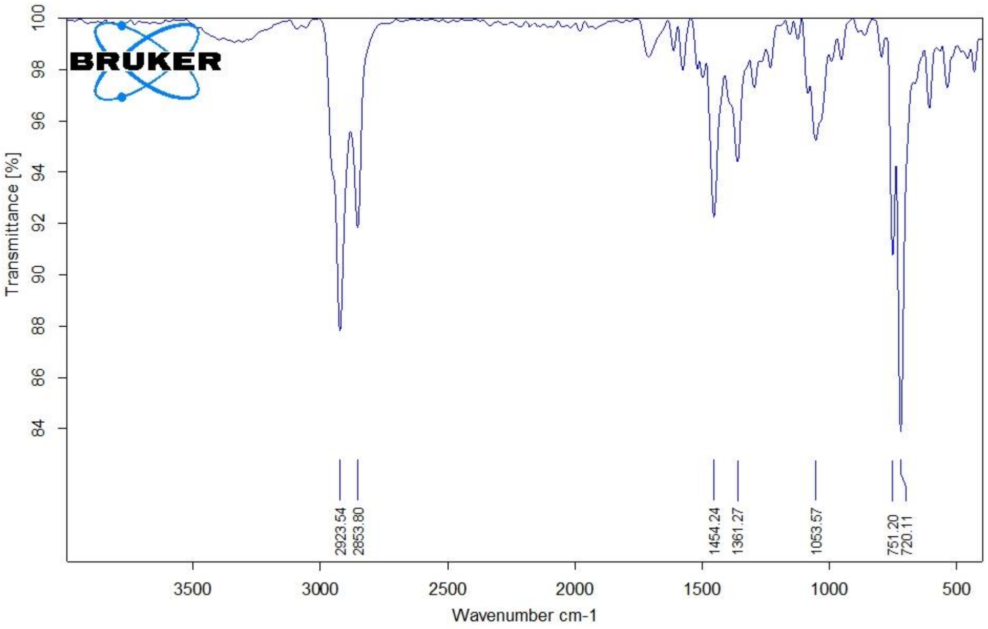
BRUKER
Transmittance [%]
100
98
96
94
92
90
88
86
84
2923.54
2853.80
1454.24
1361.27
1053.57
751.20
720.11
3500
3000
2500
2000
1500
1000
500
Wavenumber cm-1

Characterisation of 6f

1-Decyl-2-(1-methyl-1*H*-pyrrol-2-yl)-*1H*-phenanthro[9,10-*d*]imidazole

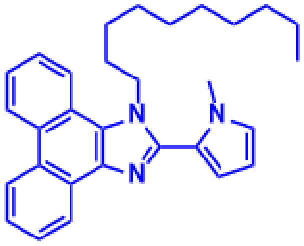

^{1}H NMR

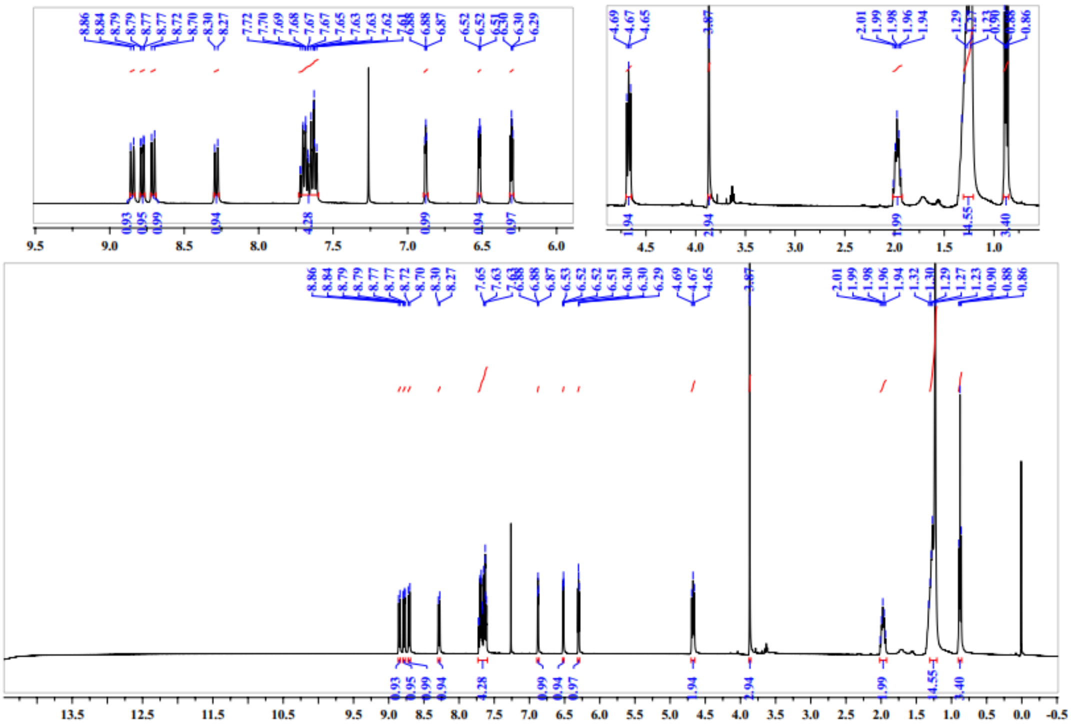

^{13}C NMR

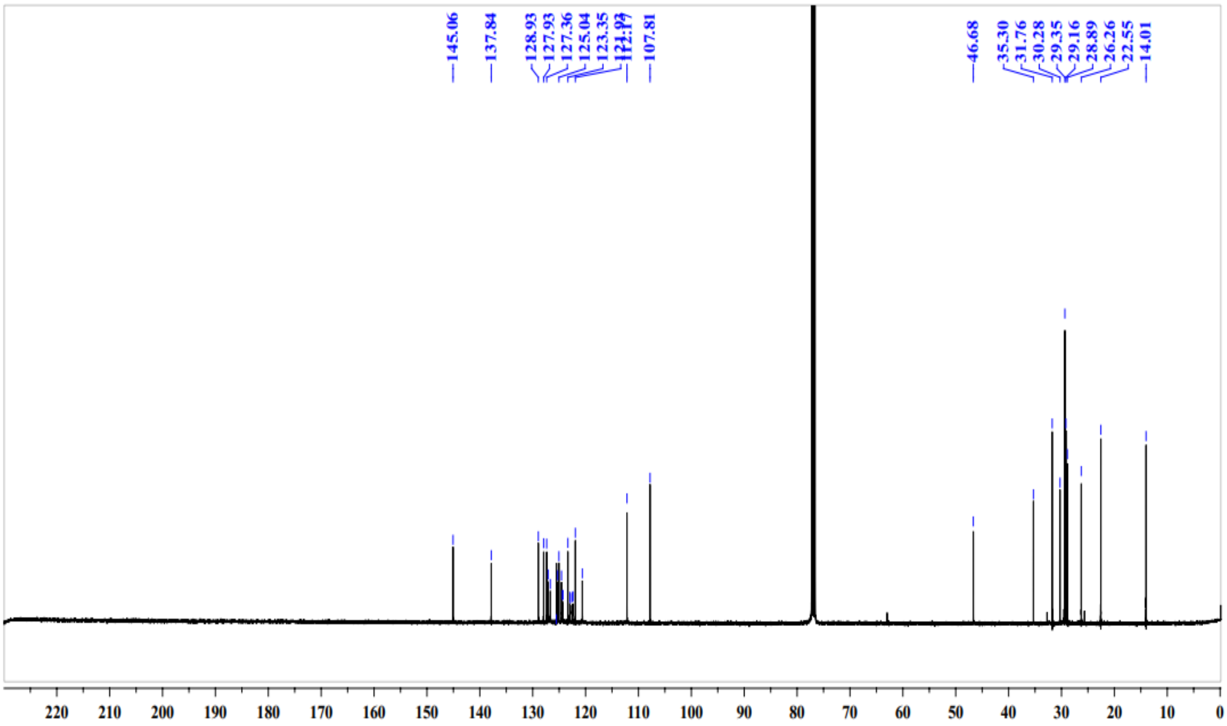
145.06
137.84
128.93
127.93
127.36
125.04
123.35
107.81
46.68
35.30
31.76
30.28
29.35
29.16
28.89
26.26
22.55
14.01
220 210 200 190 180 170 160 150 140 130 120 110 100 90 80 70 60 50 40 30 20 10 0

HRMS

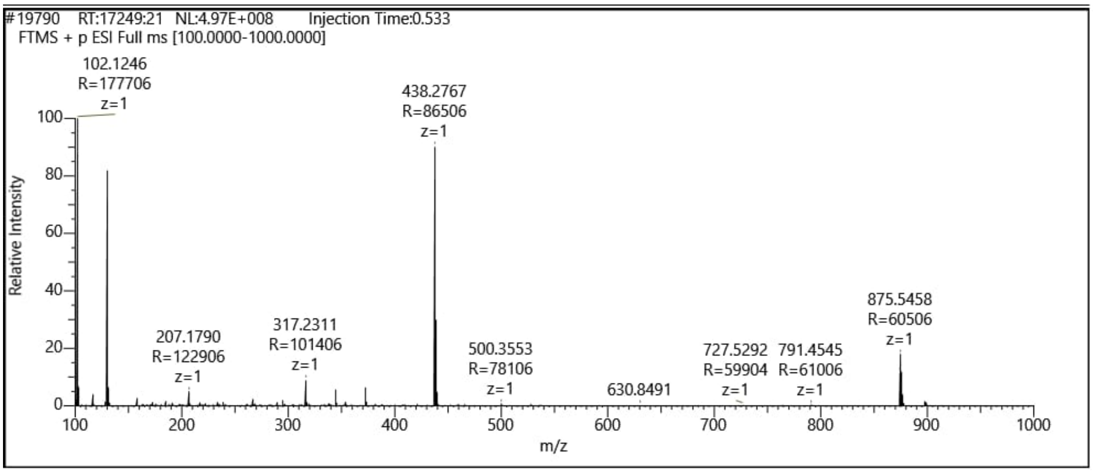
#19790 RT:17249:21 NL:4.97E+008 Injection Time:0.533
FTMS + p ESI Full ms [100.0000-1000.0000]
102.1246 R=177706 z=1
438.2767 R=86506 z=1
207.1790 R=122906 z=1
317.2311 R=101406 z=1
500.3553 R=78106 z=1
630.8491
727.5292 R=59904 z=1
791.4545 R=61006 z=1
875.5458 R=60506 z=1
Relative Intensity
m/z

FTIR

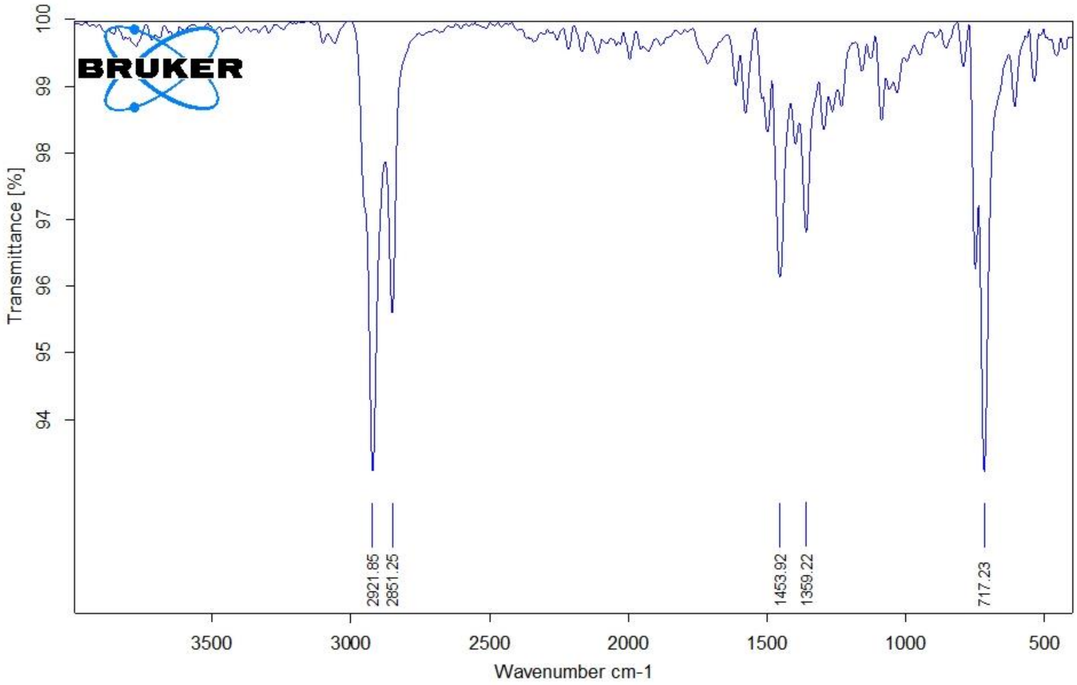

Transmittance [%]
100
99
98
97
96
95
94
BRUKER
2921.85
2851.25
1453.92
1359.22
717.23
3500
3000
2500
2000
1500
1000
500
Wavenumber cm-1

Characterisation of 6g

1-Dodecyl-2-(1-methyl-1*H*-pyrrol-2-yl)-1*H*-phenanthro[9,10-*d*]imidazole

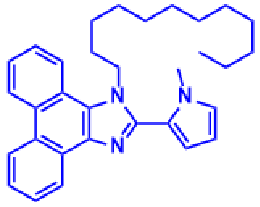

^{1}H NMR

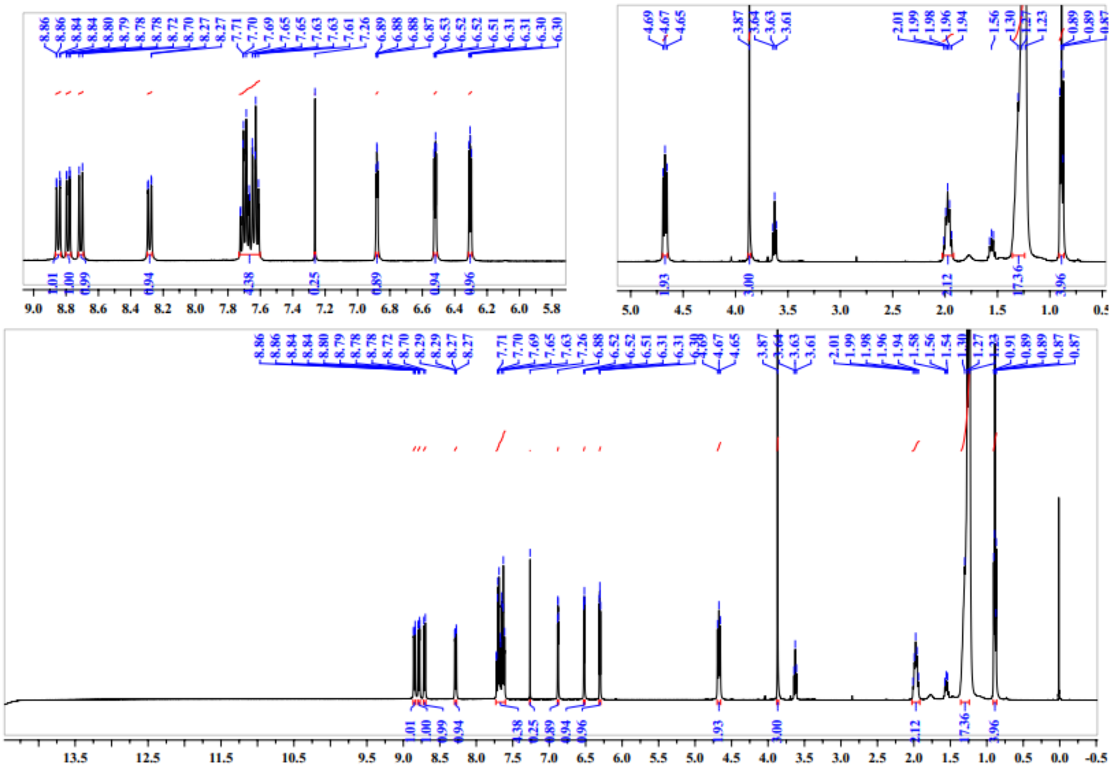

^{13}C NMR

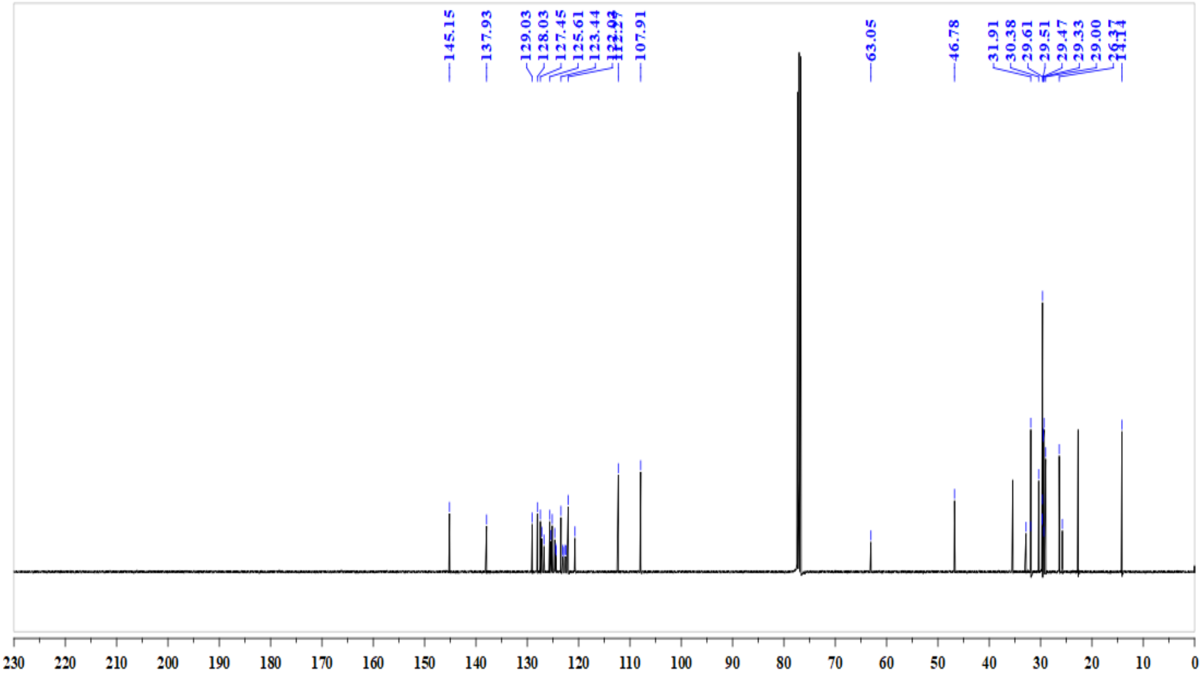
145.15
137.93
129.03
128.03
127.45
125.61
123.44
107.91
63.05
46.78
31.91
30.38
29.61
29.51
29.47
29.33
29.00
230
220
210
200
190
180
170
160
150
140
130
120
110
100
90
80
70
60
50
40
30
20
10
0

HRMS

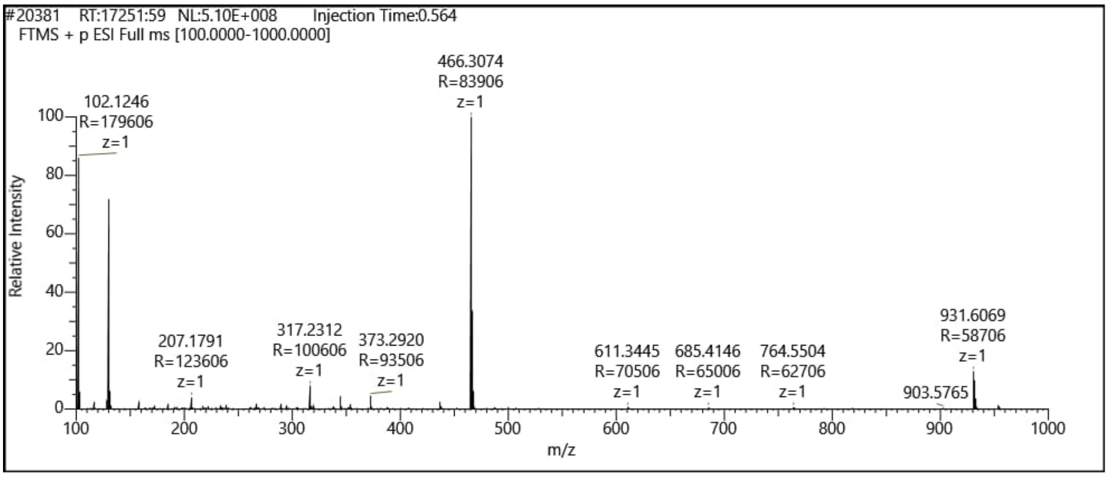
#20381 RT:17251:59 NL:5.10E+008 Injection Time:0.564
FTMS + p ESI Full ms [100.0000-1000.0000]
466.3074
R=83906
z=1
102.1246
R=179606
z=1
207.1791
R=123606
z=1
317.2312
R=100606
z=1
373.2920
R=93506
z=1
611.3445
R=70506
z=1
685.4146
R=65006
z=1
764.5504
R=62706
z=1
931.6069
R=58706
z=1
903.5765
Relative Intensity
m/z

FTIR

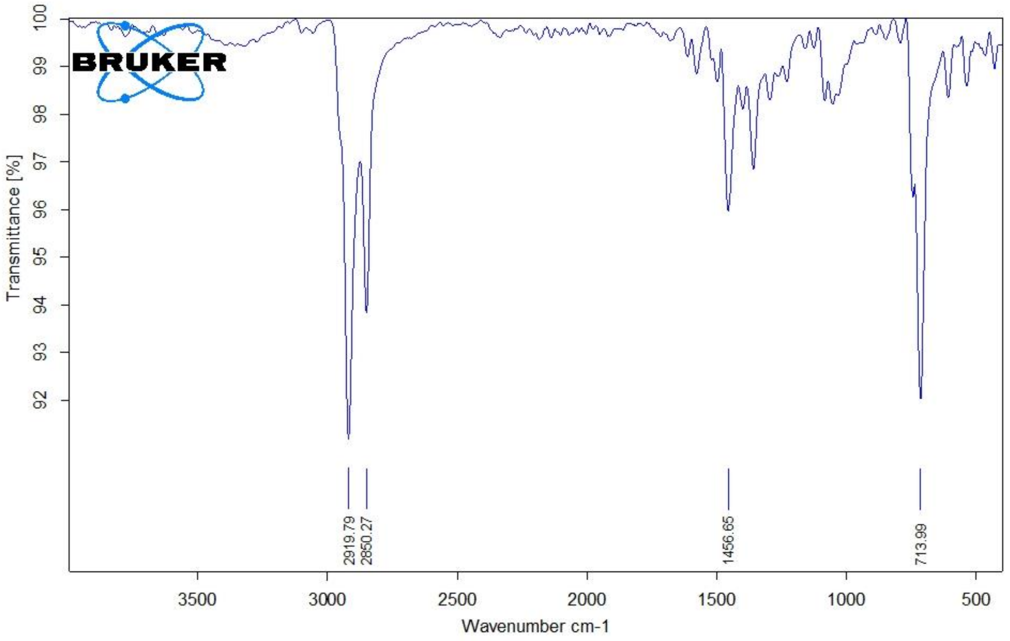

Characterisation of 6h

1-(4-Fluorophenyl)-2-(1-methyl-*1H*-pyrrol-2-yl)-1*H*-phenanthro[9,10-*d*]imidazole

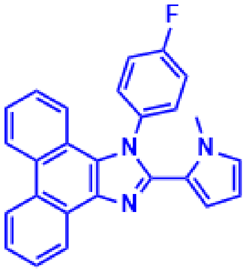

^{1}H NMR

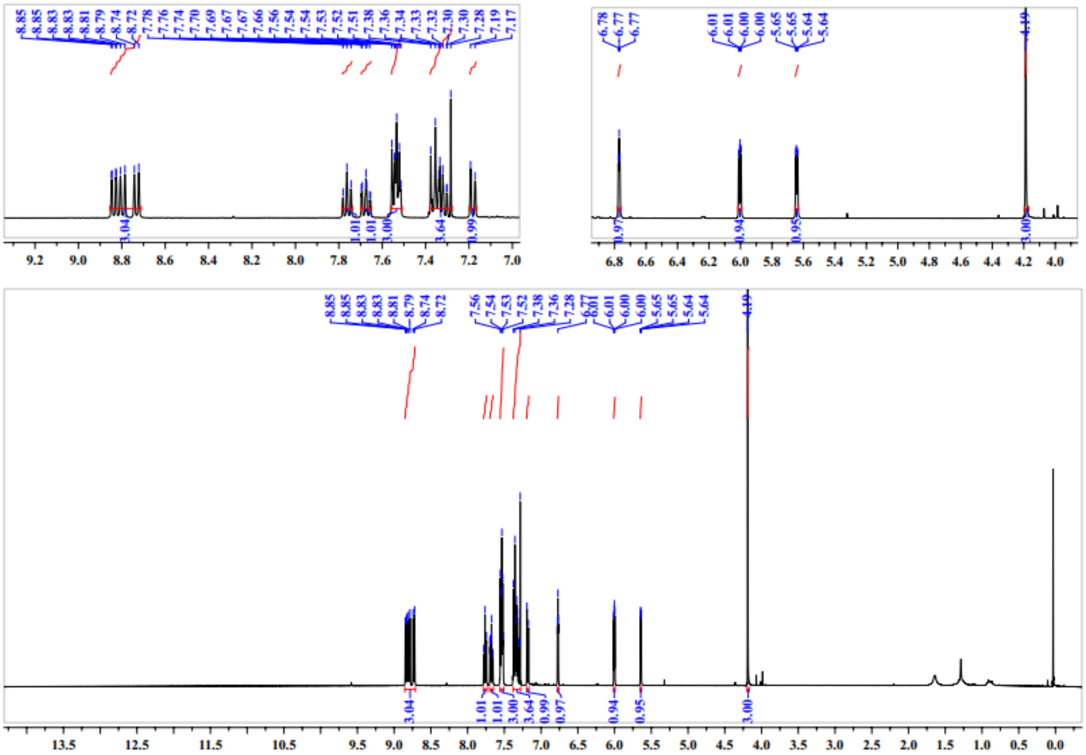

^{13}C NMR

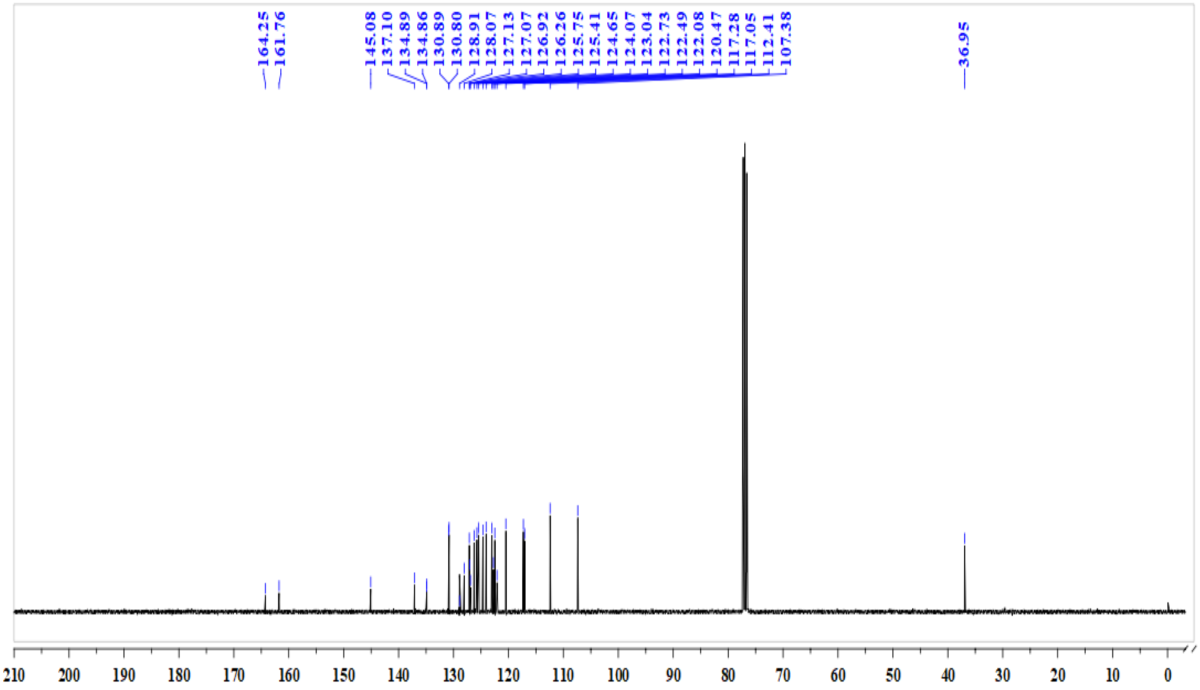
164.25
161.76
145.08
137.10
134.89
134.86
130.89
130.80
128.91
128.07
127.13
127.07
126.92
126.26
125.75
125.41
124.65
124.07
123.04
122.73
122.49
122.08
120.47
117.28
117.05
112.41
107.38
36.95
210 200 190 180 170 160 150 140 130 120 110 100 90 80 70 60 50 40 30 20 10 0

HRMS

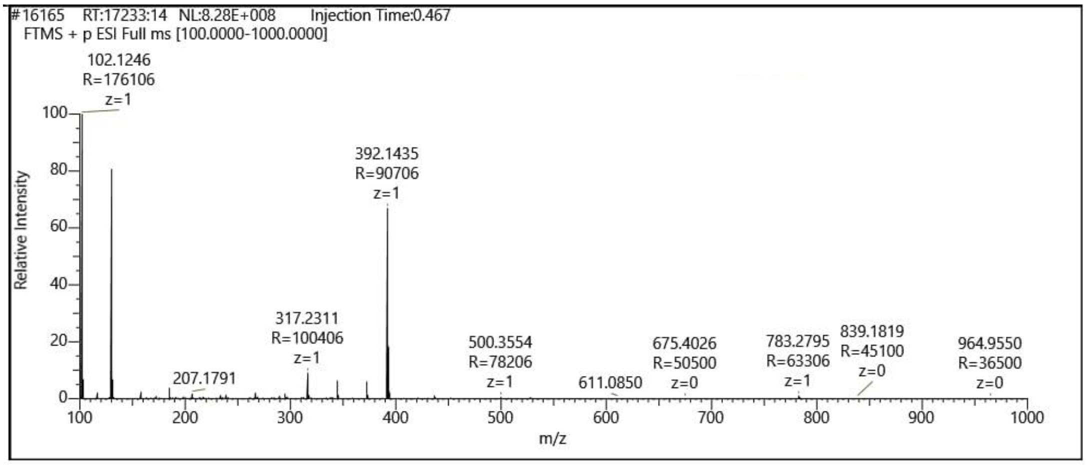
#16165 RT:17233:14 NL:8.28E+008 Injection Time:0.467
FTMS + p ESI Full ms [100.0000-1000.0000]
102.1246
R=176106
z=1
392.1435
R=90706
z=1
317.2311
R=100406
z=1
207.1791
500.3554
R=78206
z=1
611.0850
675.4026
R=50500
z=0
783.2795
R=63306
z=1
839.1819
R=45100
z=0
964.9550
R=36500
z=0
Relative Intensity
m/z

FTIR

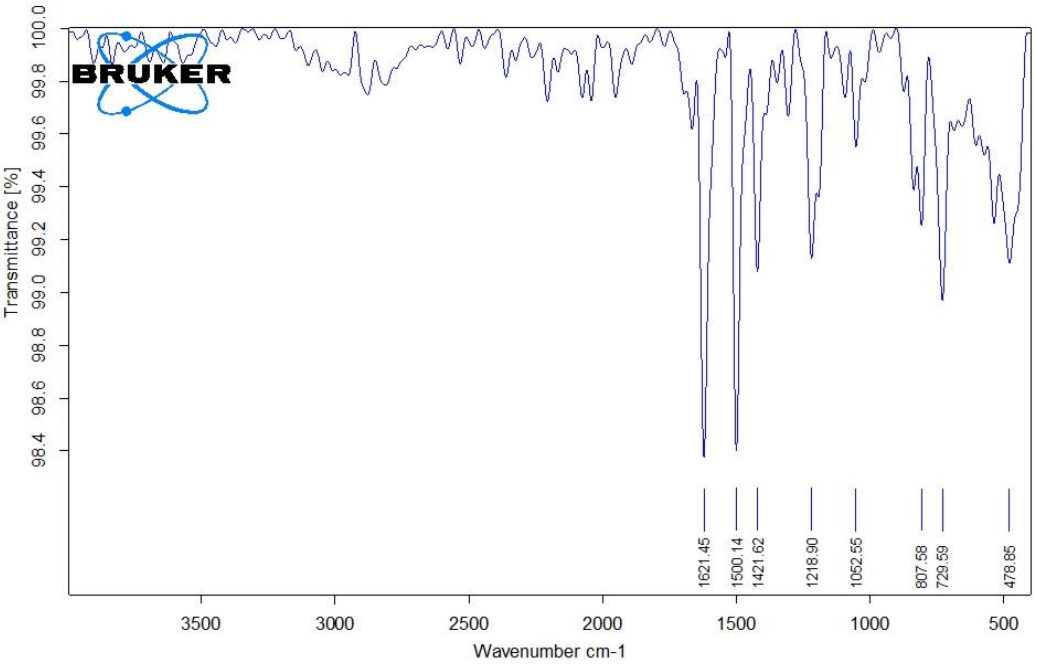

BRUKER
Transmittance [%]
100.0
99.8
99.6
99.4
99.2
99.0
98.8
98.6
98.4
1621.45
1500.14
1421.62
1218.90
1052.55
807.58
729.59
478.85
3500
3000
2500
2000
1500
1000
500
Wavenumber cm-1

Characterisation of 6i

3-(2-(1-Methyl-*1H*-pyrrol-2-yl)-*1H*-phenanthro[9,10-*d*]imidazol-1-yl)phenol

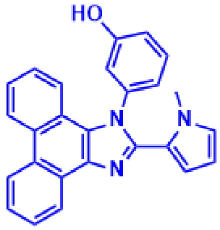

^{1}H NMR

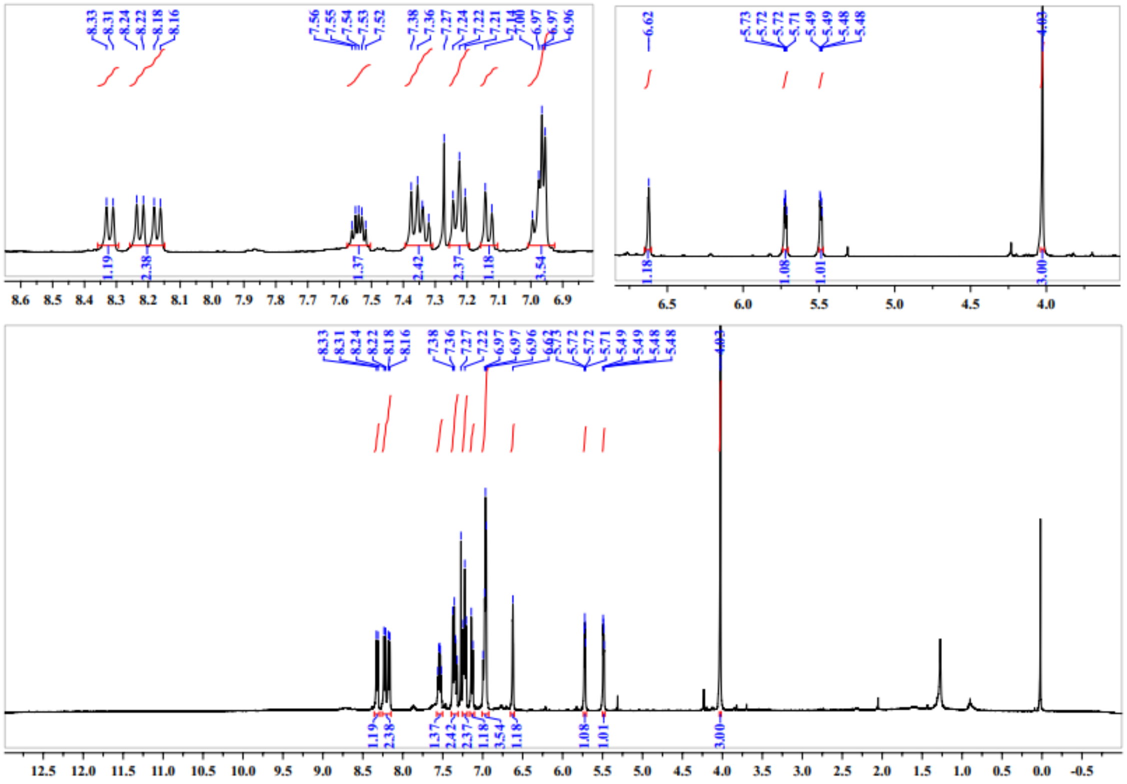

^{13}C NMR

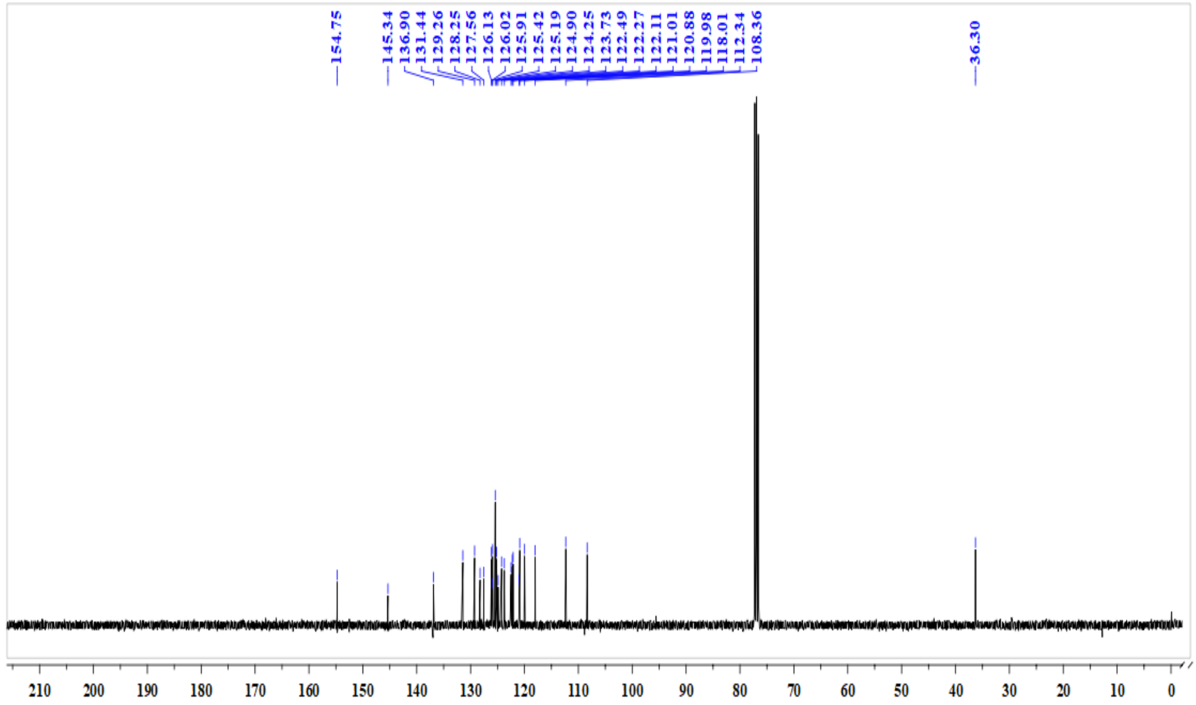
154.75
145.34
136.90
131.44
129.26
128.25
127.56
126.13
126.02
125.91
125.42
125.19
124.90
124.25
123.73
122.49
122.27
122.11
121.01
120.88
119.98
118.01
112.34
108.36
36.30
210 200 190 180 170 160 150 140 130 120 110 100 90 80 70 60 50 40 30 20 10 0

HRMS

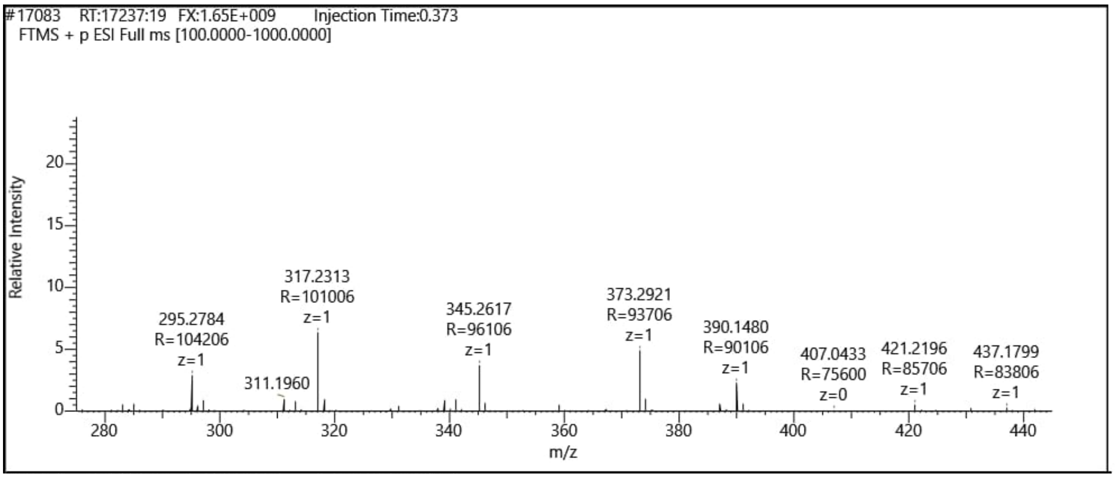
#17083 RT:17237:19 FX:1.65E+009 Injection Time:0.373
FTMS + p ESI Full ms [100.0000-1000.0000]
Relative Intensity
m/z
295.2784 R=104206 z=1
311.1960
317.2313 R=101006 z=1
345.2617 R=96106 z=1
373.2921 R=93706 z=1
390.1480 R=90106 z=1
407.0433 R=75600 z=0
421.2196 R=85706 z=1
437.1799 R=83806 z=1

FTIR

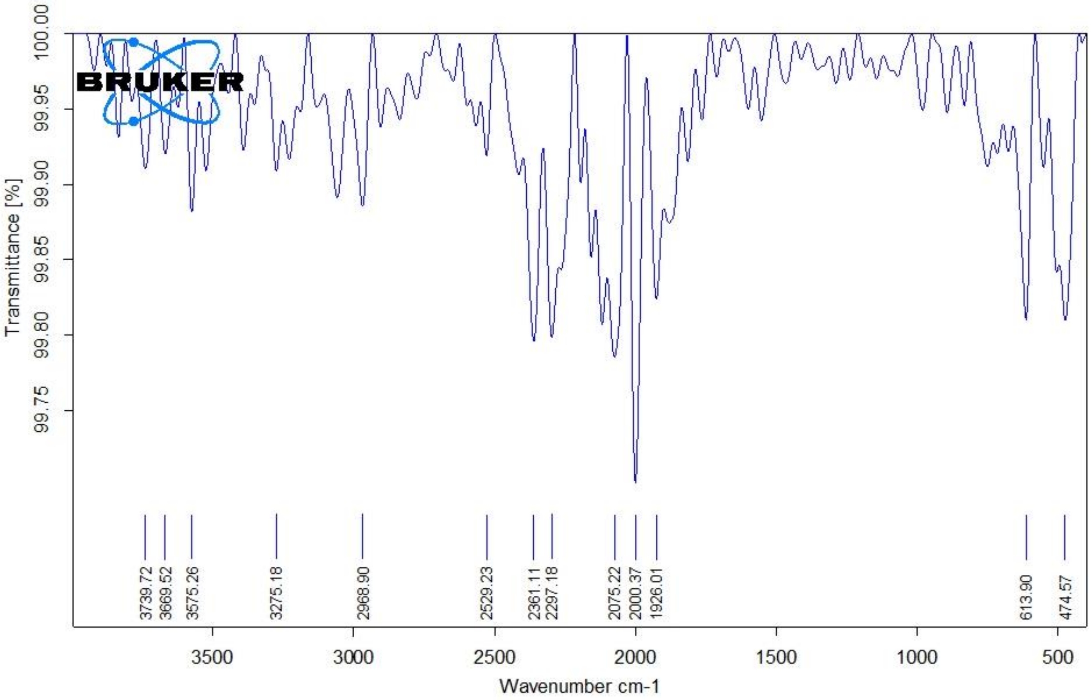

Transmittance [%]
100.00
99.95
99.90
99.85
99.80
99.75
3739.72
3669.52
3575.26
3275.18
2968.90
2529.23
2361.11
2297.18
2075.22
2000.37
1926.01
613.90
474.57
3500
3000
2500
2000
1500
1000
500
Wavenumber cm-1

Reaction mechanism

The iodine catalysed reaction mechanism is illustrated in Scheme S1 for both 3-component and 4-component reaction.

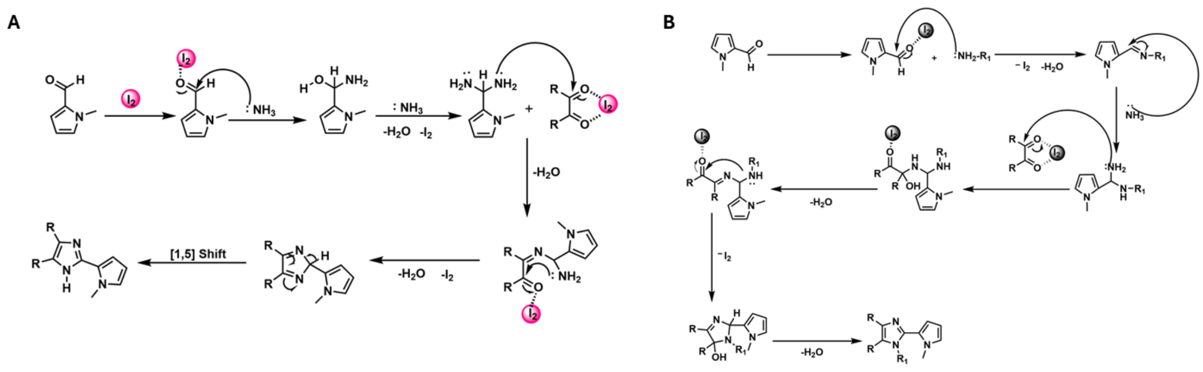

Scheme S1. Iodine catalysed reaction mechanism of A) 3 component reaction; B) 4 component reaction

Physical parameters of the synthesized compounds

Table S1. Physical parameters of 5g-6i

Sample code	Molecular weight (g/mol)	Yield (%)	Melting point (°C)
**5g**	389.49	81	134-140
**5h**	409.91	62	138-144
**5i**	454.36	65	150-154
**5j**	412.53	59	116-122
**5k**	397.56	60	-
**6a**	297.35	51	215-219
**6b**	325.41	53	125-130
**6c**	353.46	93	122-125
**6d**	381.51	52	113-117
**6e**	409.57	81	73-76
**6f**	437.62	88	-
**6g**	465.67	97	66-70
**6h**	391.44	35	161-165
**6i**	389.45	30	205-208

In vivo studies

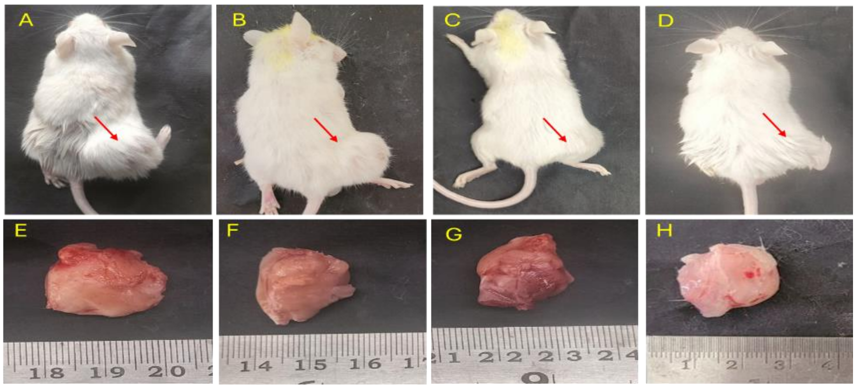

Figure S1: Photographs of BALB/c mice bearing solid tumours: (A, E) control group (B, F) Positive control group (5 FU, 20mg/Kg), (C, G) 6i1 (10mg/kg) and (D, H) 6i2 (30mg/kg)

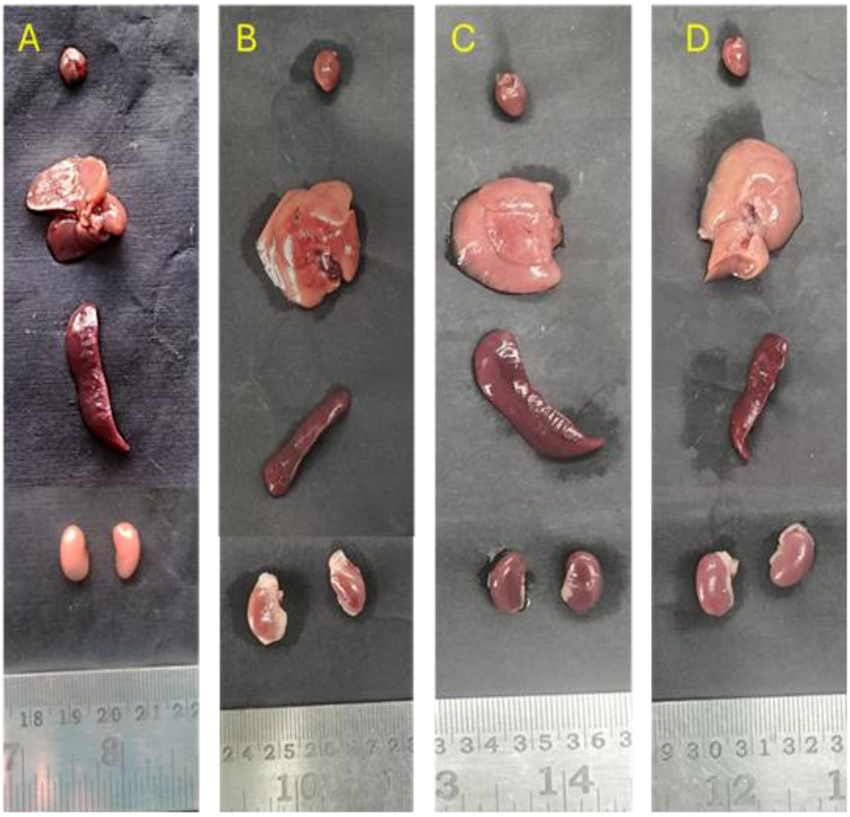

Figure S2: Heart, Liver, Spleen and Kidneys of EAC solid tumour bearing BALB/c mice: A) Control, B) Positive Control (20mg/Kg), C) 6i1 (10mg/Kg) and D) 6i2 (30mg/Kg)

Table S2. Tumor and haematological characteristics in mice injected with EAC followed by 6i administration for 14th days and dissected on 16th day.

Parameter	Reference Values	Units	Control	Positive Control (5-FU, 20 mg/Kg)	6i1 (10 mg/Kg)	6i2 (30 mg/Kg)
Hb	13-18 M<br>12-16 F	gm%	14	9	11	12
TC	5,000 to 10000	/ cmm	13000	9500	7100	14000
Platelets	1.5 to 3.5 lakhs		4.5 lakhs	4.8 lakhs	3.8 lakhs	2.9 lakhs
DC						
Polymorphs	40 -70	%	60	55	63	53
Lymphocytes	20-40	%	40	45	36	46
Eosinophils	2 -6	%	0	0	1	1
Monophills	0-1		nil	nil	nil	nil
Basophils	0-1		nil	nil	nil	nil

M: Male; F: Female; 5-FU: F-Fluorouracil; gm%: Gram percentage; cmm: Cubic millimeter; Hb: Hemoglobin; TC: Total Count; DC: Differential Count

Table S3. Variations in the biochemical parameters of control and treated BALB/c mice.

Parameter	REF Values	Control	Positive Control (5-FU, 20mg/Kg)	6i1 10mg/Kg	6i2 30mg/Kg
Blood Urea (mg%)	5–40	30	38	31	43.7
Serum Creatinine (mg%)	0–1.4	0.4	0.3	0.3	0.4
SGOT (IU/L)	5–40	148	108	97	145
SGPT (IU/L)	5–35	130	107	55	90

SGOT: Serum Glutamate Oxaloacetate Transaminase; SGPT: Serum Glutamate Pyruvate Transaminase; IU: International Unit

In silico studies

Molecular Docking studies

Table S4. Binding energy of molecule 5d against various breast cancer proteins.

Protein	Binding Affinity (kcal/mol)
4KZN	-5.9
5GPG	-9.4
7NH5	-9.5
5W9C	-8.8

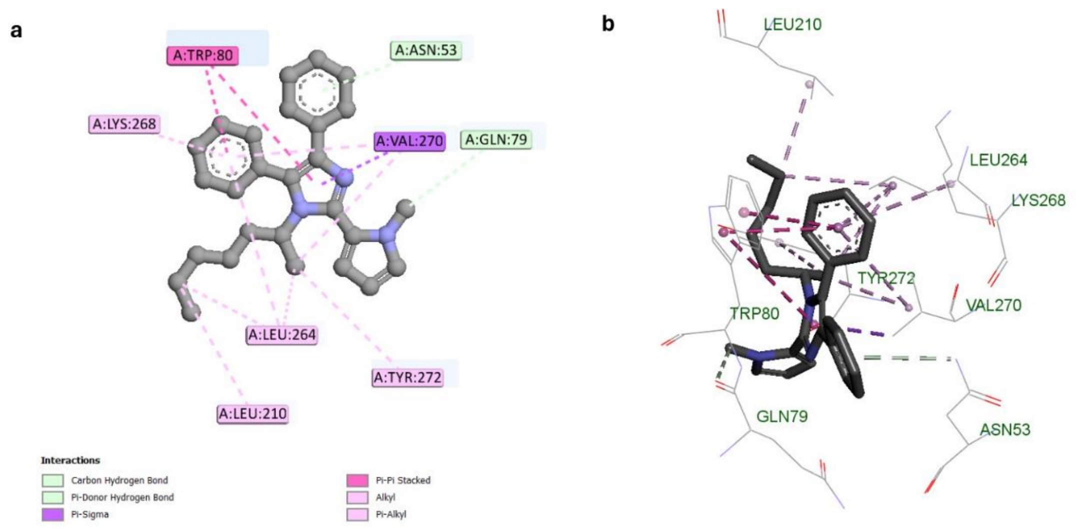

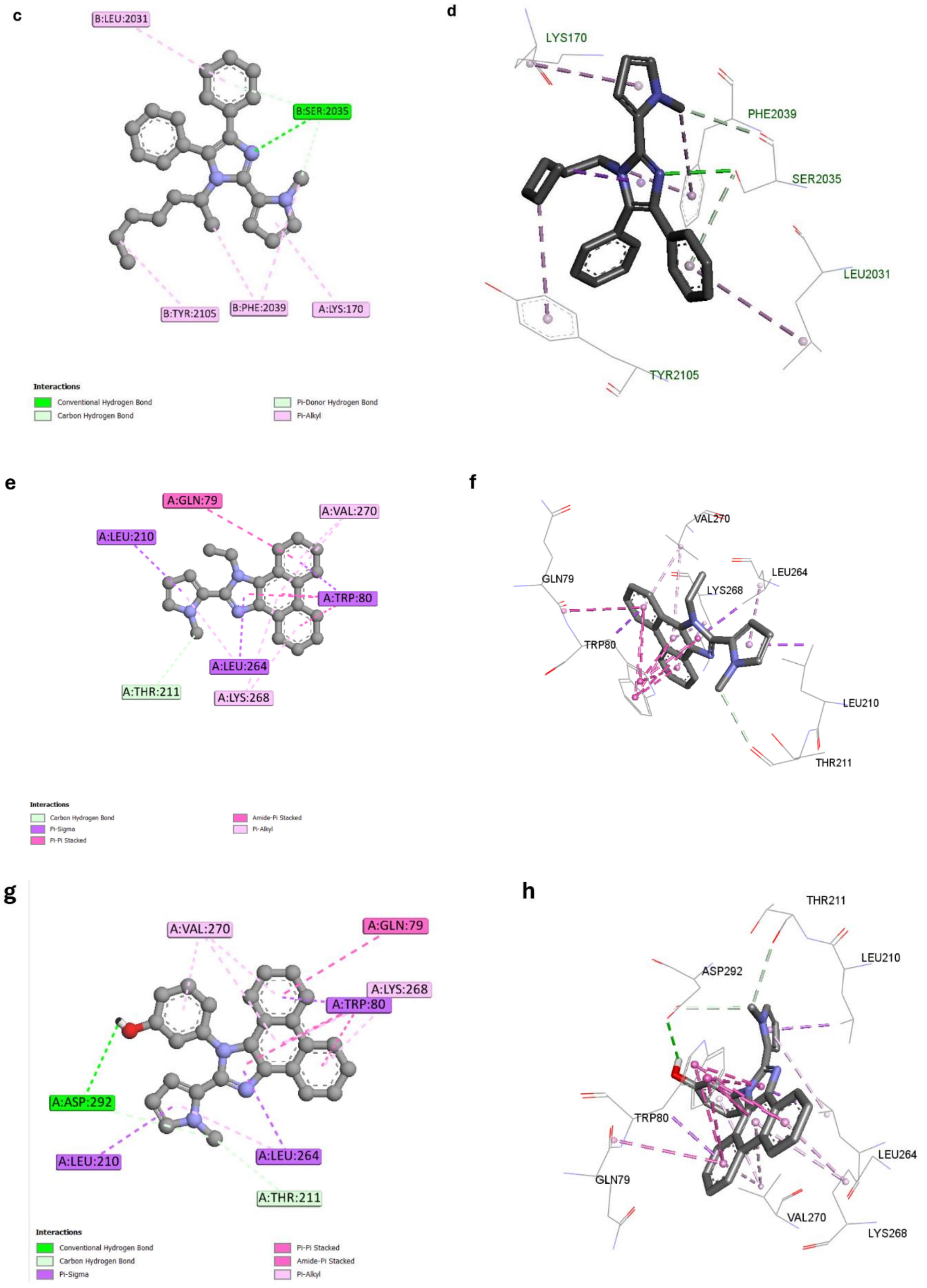
c
B:LEU:2031
B:SER:2035
B:TYR:2105
B:PHE:2039
A:LYS:170
Interactions
Conventional Hydrogen Bond
Carbon Hydrogen Bond
Pi-Donor Hydrogen Bond
Pi-Alkyl
d
LYS170
PHE2039
SER2035
LEU2031
TYR2105
e
A:GLN:79
A:VAL:270
A:LEU:210
A:TRP:80
A:LEU:264
A:THR:211
A:LYS:268
Interactions
Carbon Hydrogen Bond
Pi-Sigma
Pi-Pi Stacked
Amide-Pi Stacked
Pi-Alkyl
f
VAL270
GLN79
LEU264
LYS268
TRP80
LEU210
THR211
g
A:VAL:270
A:GLN:79
A:LYS:268
A:TRP:80
A:ASP:292
A:LEU:210
A:LEU:264
A:THR:211
Interactions
Conventional Hydrogen Bond
Carbon Hydrogen Bond
Pi-Sigma
Pi-Pi Stacked
Amide-Pi Stacked
Pi-Alkyl
h
THR211
LEU210
ASP292
TRP80
LEU264
GLN79
VAL270
LYS268

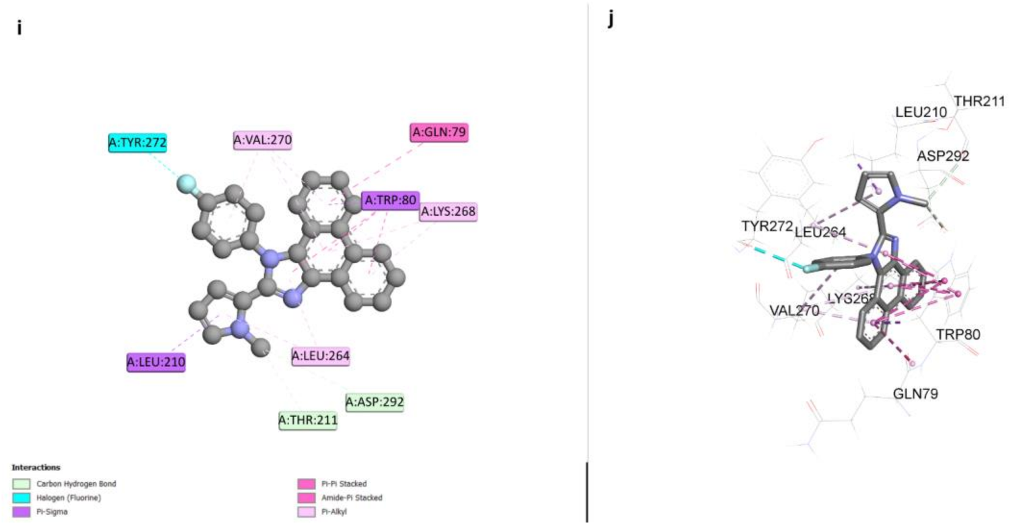

Figure S3: 2D (a, c, e, g) and 3D (b, d, f, h) structures of Compound 5d, 5k, 6b, 6i and 6h with 7NH5 receptors

DFT Calculations

Table S5. DFT calculation for activity molecules

Compound	Total Energy (Ha)	HOMO Energy (eV)	LUMO Energy (eV)	HOMO-LUMO gap (eV)	Dipole Mag (A m^2)
5a	- 936.48	- 5.07	- 1.13	3.93	0.55
5d	- 1250.90	- 5.45	- 0.84	4.60	1.48
5k	- 1211.60	- 5.10	- 0.88	4.22	1.12
6b	-1013.907	-5.163	-1.138	3.998	0.826
6e	-1249.726	-5.142	-1.120	4.022	0.914
6f	-1328.332	-5.140	-1.118	4.022	0.921
6i	-1241.539	-5.053	-1.126	3.927	0.708

Table S6. *In silico* analysis of the physicochemical and pharmacokinetic properties of imidazole conjugates (5a-6i)

Code	Lipinski's Rule				Veber Rule		Pharmacokinetics			Drug likeness
	MW < 500	MLogP ≤ 4.15	nHBA ≤ 10	nHBD ≤ 5	nRB ≤ 10	TPSA ≤ 140 Å²	GI	BBB	LogKp -5.26 cm/s	Bioavailability score
**5a**	299.37	2.88	1	1	3	33.61	high	yes	-5.41	0.55
**5b**	355.48	3.75	1	0	6	22.75	high	no	-4.95	0.55
**5c**	369.50	3.96	1	0	7	22.75	low	no	-4.65	0.55
**5d**	411.58	4.56	1	0	10	22.75	low	no	-3.75	0.55
**5e**	393.46	4.59	2	0	4	22.75	low	no	-4.50	0.55
**5f**	391.46	3.64	2	1	4	42.98	high	yes	-5.08	0.55
**5g**	389.49	4.42	1	0	4	22.75	low	no	-4.56	0.55
**5h**	409.91	4.69	1	0	4	22.75	low	no	-4.50	0.55
**5i**	454.36	4.79	1	0	4	22.75	low	no	-4.73	0.55
**5j**	412.53	2.48	3	0	6	35.22	high	yes	-6.42	0.55
5k	397.56	4.36	1	0	8	22.75	low	no	-4.15	0.55
6a	297.34	3.31	1	1	1	33.61	high	yes	-4.97	0.55
6b	325.41	3.75	1	0	2	22.75	high	yes	-4.96	0.55
6c	353.46	4.18	1	0	4	22.75	high	No	-4.51	0.55
6d	381.51	4.59	1	0	6	22.75	low	no	-3.91	0.55
6e	409.57	4.99	1	0	8	22.75	low	no	-3.31	0.55
6f	437.62	5.38	1	0	10	22.75	low	no	-2.71	0.55
6g	465.67	5.76	1	0	12	22.75	low	no	-2.12	0.55
6h	391.44	5.02	1	0	2	22.75	low	no	-4.33	0.55
6i	389.45	4.07	2	1	2	42.98	high	no	-4.64	0.55
